# Spontaneous space closure after extraction of permanent first molars in children and adolescents: a systematic review and meta-analysis

**DOI:** 10.1093/ejo/cjae054

**Published:** 2024-10-09

**Authors:** Blend Hamza, Spyridon N Papageorgiou, Raphael Patcas, Marc Schätzle

**Affiliations:** Clinic of Orthodontics and Pediatric Dentistry, Center for Dental Medicine, Plattenstrasse 11, 8032 Zürich, Switzerland; Clinic of Orthodontics and Pediatric Dentistry, Center for Dental Medicine, Plattenstrasse 11, 8032 Zürich, Switzerland; Clinic of Orthodontics and Pediatric Dentistry, Center for Dental Medicine, Plattenstrasse 11, 8032 Zürich, Switzerland; Clinic of Orthodontics and Pediatric Dentistry, Center for Dental Medicine, Plattenstrasse 11, 8032 Zürich, Switzerland

**Keywords:** molar incisor hypomineralization, dental caries, first molar, tooth extraction, space closure, orthodontics

## Abstract

**Background:**

Extraction of the permanent first molars is sometimes necessitated in children and adolescents due to deep carious lesions or developmental defects.

**Objective:**

To estimate the prevalence of spontaneous space closure after extraction of permanent first molars and identify factors associated with it.

**Search methods:**

Unrestricted searches in five databases for human studies until February 2024.

**Selection criteria:**

Longitudinal before-and-after (cohort) human studies assessing eruption of the permanent second molars and spontaneous space closure after extraction of the permanent first molar.

**Data collection and analysis:**

Study selection, data extraction, and risk of bias assessment were performed in duplicate. Random-effects meta-analyses of average spontaneous space closure prevalences and odds ratios (OR) with their 95% confidence intervals (CI) were performed, followed by meta-regression/sensitivity/reporting biases’ analyses and evaluation of our confidence in effect estimates.

**Results:**

Sixteen reports pertaining to 15 studies (1 prospective /14 retrospective) were included covering 1159 patients (ages 5.5–15.0 years [mean 10.0 years]; 45% male on average) and 2310 permanent second molars. The prevalence of spontaneous space closure was higher in the maxilla (nine studies; 85.3%; 95% CI = 73.7%–92.3%) than the mandible (11 studies; 48.1%; 95% CI = 34.5%–62.0%) to a significant extent (nine studies; OR = 7.77; 95% CI = 4.99–12.11; *P* < 0.001). For both maxillary/mandibular second molars, Demirjian category E was associated with increased space closure odds than earlier/later stages (*P* < 0.05). Spontaneous space closure in the mandible was seen more often for patients ages 8–10 years (compared with older patients; three studies; OR = 3.32; 95% CI = 1.73–6.36; *P* < 0.001) and when the mandibular permanent third molar was present (four studies; OR = 2.28; 95% CI = 1.67–3.09; *P* = 0.003). Additional analyses failed to find any significant modifying factors.

**Limitations:**

The quality of evidence was very low in all instances due to the inclusion of retrospective studies with methodological issues.

**Conclusions:**

Existing evidence indicates that spontaneous space closure in children and adolescents after extraction of the permanent first molar is seen more often in the maxilla than the mandible. Extraction of the permanent first molar at the Demirjian stage E of the second molar and presence of the lower permanent third molar is associated with increased odds of space closure, but uncertainty persists, due to methodological issues of existing studies.

**Registration:**

PROSPERO (CRD42023395371).

## Introduction

Existing data indicate that compromised (weakened by significant tooth structure loss) first permanent molars are found in 10% of children according to general dentists or 26% of children according to specialist pediatric dentists [1]. Compromised permanent first molars can negatively affect the child’s general health and social well-being, while they confer significant financial burden to the patient and health services [2, 3]. Compromised permanent first molars can interfere with daily functions of the children including school attendance, eating, or sleeping, while they might cause pain and impact the child’s quality of life [4]. Caries and molar incisor hypomineralization [5] are the main etiological reasons for the poor prognosis of these teeth and for such cases the treatment options also include the extraction of these teeth [6].

Although extraction of a compromised permanent first molar seems to be a radical approach, it might actually prove to be cost-effective in comparison to repeated restorations and takes the usually poor long-term prognosis of such restored molars and the possible psychological impact of intensive restorative dental work on the child into consideration [7]. Additional reasons for the extraction of permanent first molars might present extensive caries or restoration, endodontically treated molars prior to completion of root formation, or molars with apical pathology [8, 9]. Regardless of indication, extraction of the permanent first molar should be carefully timed in order to improve the odds of the permanent second molar spontaneously aligning distally to the second premolar—i.e. so that a spontaneous space closure happens [8, 9]. However, even if the permanent second molar aligns spontaneously, this is often accompanied by residual spaces, unfavorable tipping and rotation of the second molar and the premolars, necessitating a subsequent orthodontic treatment [10, 11].

Orthodontic treatment for permanent first molar extraction cases can be time-consuming, as the protraction of the permanent second molar may add about 10 months to the treatment [12, 13]. One of the most important issues is anchorage management, especially in Class II malocclusions, and extraction of mandibular permanent molars might necessitate the use of anchorage reinforcement methods, including extraoral traction, Nance buttons [14], fixed functional appliances, or temporary anchorage devices [15, 16]. Furthermore, extended orthodontic tooth movement of the permanent second molar to close the first molar space might be associated with increased periodontal adverse effects, including increased pocket depths, gingival recession, alveolar bone loss, and root resorption [17]. Finally, after orthodontic space closure placement of a fixed retainer seems prudent to minimize the risk of space re-opening [14]. Therefore, minimization of the need or the extent of orthodontic treatment might be beneficial to the patient.

Previous systematic reviews have reported that spontaneous space closure after extraction of the permanent first molar in the mandible might be expected when the latter is done between 8 and 11.5 years of age [18]. A subsequent systematic review [19] concluded that ideal time for the extraction of the permanent first molar is when the permanent second molar is at the early bifurcation stage (Demirjian stage E) in order to achieve spontaneous space closure. However, these systematic reviews covered literature published up to 2017 and performed limited or no meta-analysis.

### Objective

The aim of this systematic review was to assess the prevalence of spontaneous space closure after extraction of a permanent first molar before the eruption of the permanent second molar in children / adolescents and identify factors associated with spontaneous space closure.

## Methods

### Protocol and registration

The review’s protocol was developed a priori, registered in PROSPERO (CRD42023395371), and all post hoc protocol deviations were transparently reported (Supplementary Appendix 1). This review was guided by the Cochrane Handbook [20] and the Preferred Reporting Items for Systematic reviews and Meta-Analyses (PRISMA) 2020 statement [21] for its conduct and reporting, respectively.

### Eligibility criteria

Eligible to be included in this review were clinical studies on children / adolescents (< 18 years of age) of any sex/ethnicity, where at least one permanent first molar of either jaw was extracted for any reason prior to the emergence of the permanent second molar and followed patients up to the complete eruption of the latter. Included were randomized trials and non-randomized before-and-after (cohort) studies (both prospective and retrospective), while excluded were case series/reports, animal studies, and non-clinical studies. Excluded were studies on adults, patients with systemic diseases, patients with agenesis of the first and/or second permanent molar, and patients receiving orthodontic or pedodontic treatment (except extraction of the first permanent molar). Any clinical setting was included to increase the generalizability of the study’s results.

### Information sources and search

Five databases were searched without restrictions for publication language / year / type from inception up to February 2024 (Supplementary Appendix 2). The reference lists and citation lists through Google Scholar of eligible articles and existing systematic reviews were manually reviewed to identify any potentially relevant studies to include.

### Selection process

Initially, the title or abstract of identified studies was checked to eliminate obviously irrelevant to the review studies. Subsequently, the full text of all remaining studies was checked against the review’s eligibility criteria for potential inclusion. Study selection was performed in duplicate (BH/SNP) and independently, while any disagreements were resolved through discussion with a third author (MS).

### Data collection process and items

Data collection utilized a pre-defined and piloted extraction form, encompassing the following data: (a) study characteristics, including the primary author with the year of publication, study design, and clinical setting (country); (b) patient characteristics, comprising age and sex; (c) sample size in terms of patients and teeth; (d) reasons for extraction of the first permanent molar; (e) follow-up duration; (f) pre-extraction variable collection methods; (g) post-extraction outcome measurement methods; and (h) outcomes assessed. Data extraction was likewise performed independently by two authors (BH/SNP), while any disparities were resolved through discussion with a third author (MS). The corresponding authors of studies that included a large sample and evaluated potential confounders were contacted to request either individual patient data or adjusted for clustering estimates for the effect of confounders (Supplementary Appendix 3).

### Risk of bias of individual studies

As the review aimed to assess the prevalence of spontaneous space closure, the risk of bias of all included studies (irrespective of their design) was assessed with the Joanna Briggs Institute’s tool for prevalence studies [22]. All assessments were performed by two authors independently (BH/SNP), with discrepancies resolved through discussion with a third author (MS).

### Effect measures and data synthesis

A single-group meta-analysis for pooled spontaneous space closure rate (one-group pooling), followed by a pairwise meta-analysis (two-group comparison) with odds ratio (OR) and their corresponding 95% confidence intervals (CI), was undertaken. As the spontaneous space closure prevalence was expected to vary among studies (according to different starting position and inclination of the molar, extraction timing, available space, and bone characteristics) a random-effects model was deemed a priori more appropriate to capture this variability and calculate the average distribution of treatment effects across studies [23]. For one-group meta-analysis of proportions a random intercept logistic regression model was used [24], while for the two-group pairwise meta-analysis a novel restricted maximum likelihood variance estimator was chosen [25] (and the Knapp and Hartung adjustment [26] for meta-analyses with >3 studies). Heterogeneity between studies was assessed through visual inspection of a contour-enhanced forest plot (Supplementary Appendix 1) [27] and through estimation of tau^2^ (absolute heterogeneity) or *I*^2^ (relative inconsistency) with their uncertainty intervals. Ninety-five percent predictions were calculated, which incorporate identified heterogeneity and assist in the interpretation of the meta-analytical estimates by providing a range of expected effects across various future clinical settings [28]. Random-effects meta-regressions were conducted for pooled prevalence of spontaneous space closure of either jaw to investigate the effect of mean age or patient sex (through the % of male patients in the sample). All analyses were conducted in R 4.2.2. (R Foundation for Statistical Computing, Vienna, Austria) by one author (SNP), with open dataset [29], alpha set at 0.05 (0.10 for meta-regressions) and a two-sided *P*-value (Supplementary Appendix 1).

### Reporting bias assessment and certainty assessment

Hints of reporting biases (including the possibility of publication bias) were assessed through contour-enhanced funnel plots and formally with Thompson’s test for meta-analyses with at least 10 studies. The Grades of Recommendations, Assessment, Development, and Evaluation (GRADE) approach was employed [30] to gauge the certainty around the results of pairwise (two-group) meta-analyses and findings were summarized using a revised format [31]. The GRADE approach was not used for the one-group pooled prevalences, since no formal guidance exists.

## Results

### Study selection

**Figure 1.**
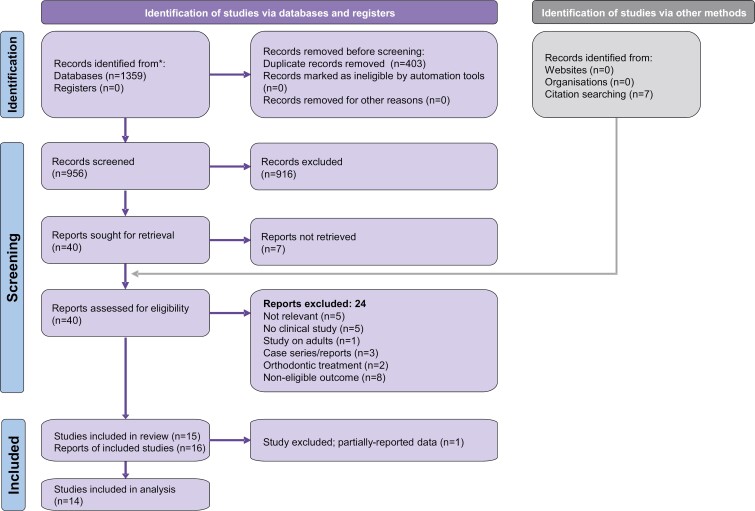
PRISMA flow diagram for the identification and selection of eligible studies.

The initial electronic database search yielded 1359 records and seven additional were identified through manual searching (Fig. 1). After eliminating 403 duplicates, 956 records were left for further evaluation and were assessed against the eligibility criteria (Supplementary Appendix 4). Ultimately, 16 publications, corresponding to 15 distinct clinical studies, were included in the quantitative synthesis. Most (13/16) were published as journal papers, while one was published as conference proceedings [32], one as Master’s thesis [33], and one both as Master’s thesis and as journal paper [34, 35]. The corresponding authors of 6 included studies were contacted, but only one responded (Supplementary Appendix 3) and provided the study’s raw dataset [36].

### Study characteristics

The characteristics of included studies are summarized in Table 1. Among the 15 included studies, the majority (93%; 14/15) were retrospective non-randomized cohort studies and one was a prospective cohort study. These studies were conducted mostly in university clinics or hospitals from eight different reported countries (Belgium, Canada, England, Norway, Palestine, Sweden, Turkey, and the United States of America). In total 1159 patients were included in the 15 studies (median 59 patients / study from the 14 studies reporting this), who were 45% male (484/1067; from the 12 studies reporting sex) and were on average 10.0 years of age (from the 13 studies reporting on age). A total of 2310 second permanent molars were assessed, with a median of 1.74 teeth per patient. The majority of studies (73%; 11/15) included molars of either jaw, while the remaining included only either mandibular (20%; 3/15) or maxillary molars (7%; 1/15). The majority (60%; 9/15) of studies extracted first permanent molars for various reasons and the median follow-up post-extraction was 4.0 years. Pre-extraction data collection was based in orthopantomograms in all instances, which was supplemented by clinical examination (*n* = 4; 27%) or other radiographs (*n* = 3; 20%). Post-extraction data collection was based on radiographs (*n* = 13; 87%), for the most part orthopantomograms, or clinical examination (*n* = 10; 67%). Spontaneous space closure was assessed from all studies, while other measured variables included height/inclination/position of the second permanent molar or the premolars, the development stage of the second permanent molar with either the Demirjian [37] or the Nolla classification [38], and the presence of the third molar.

**Table 1.** Characteristics of included studies.

Study	Design; source; country	Patients (teeth); jaw	Age^†^	Sex	Reason	Follow-up (years)^†^	Pre-extraction data collection	Post-extraction data collection	Outcomes
Aldahool 2024	rNRS; registry; Sweden	155 (203); Max/Mnd	11.5* [6.0, 15.0]	47.0^§^	Various	[4.0–14.0]	OPG or PA or BW	OPG or PA or BW	SC_cat_; M2 hei/ incl; PM2 pos; M1/2 dev
Brusevold 2022	rNRS; Uni; Norway	27 (90); Max/Mnd	8.7 [5.5, 12.1]	NR	MIH	3.5	Clin; OPG	Clin; OPG	SC_bin_
Canpolat 2020	rNRS; Uni; Turkey	39 (50); Mnd	10.2 [7.0, 13.0]	53.8	Various	2.0	OPG; PA	Clin; photographs	SC_cat_; M2/PM2 rot; MLdev
Ciftci 2021	rNRS; Uni; Turkey	133 (177); Mnd	9.4 [6.1, 10.8]	55.6	NR	3.5	OPG	Clin; OPG	SC_bin_; M2 incl; M1 dev; M3 pres
Ertugrul 2022	rNRS; Uni; Turkey	26 (40); Max/Mnd	10.0 [7.0, 13.8]	42.3	Various	5.1	OPG	Clin; OPG	SC_bin_
Gaudreau 2022	pNRS; Hosp; Canada	39 (62); Max/Mnd	8.8 [6.0, 12.0]	41.0	Various	NR	OPG	Clin; OPG	SC_bin_; M2 dev; M3 pres; M2/PM2 incl
Jalevik 2007	rNRS; Hosp / Uni; Sweden	33 (70); Max/Mnd	8.2 [5.6, 12.7]	44.4	MIH	5.7	Clin; OPG; BW	Clin; OPG; BW	SC_bin_
Lenaker 2022; 2023	rNRS; Hosp; United States	134 (182); Mnd	9.1 [6.0, 12.4]	38.1	Various	4.6	OPG	BW	SC_bin_; M2 dev; M2 incl
Mouroutsou 2018	rNRS; Uni; Belgium	37 (109); Max/Mnd	10.7 [7.8, 13.3]	32.4	Various	3.3 [1.8, 5.5]	OPG	Clin; OPG	SC_cat_; M2 rot; M2 incl; M2 dev
Nordeen 2022	rNRS; Uni; United States	162 (306); Max/Mnd	10.7 [6.7, 14.9]	48.0	NR	4.1	OPG	Radiograph (unspecified)	SC_bin_; M2 incl; M2 dev; M3 pres
Patel 2017	rNRS; various; United States	81 (301); Max/Mnd	9.6 [6.0, 14.5]	40.7	Various	4.0 [0.9, 7.5]	OPG	Clin; OPG	SC_bin_; M2 incl; M3 pres
Rahhal 2014	rNRS; Uni; Palestine	NR (52); Max	10.5 [NR]	NR	NR	NR	OPG	OPG	SC_bin_
Serindere 2019	rNRS; Uni; Turkey	55 (83); Max/Mnd	11.0 [8.0, 13.0]	50.9	NR	2.7 [NR]	OPG	OPG	SC_bin_; M2 dev
Teo 2013	rNRS; Hosp; Great Britain	63 (236); Max/Mnd	8.9 [7.0, 13.0]	NR	Various	4.8 [NR]	Clin; OPG	Clin	SC_bin_; M2 dev
Thilander 1970	rNRS; Hosp; Sweden	175 (349); Max/Mnd	NR	41.1	Various	NR	Clin; OPG	Clin; OPG	SC_bin_; M3 pres

bin, binary; BW, bitewing radiograph; cat, categorical; clin, clinical; dev, development; hei, height; Hosp, hospital; incl, inclination; MIH, molar incisor hypomineralization; MLdev, midline deviation; NR, not reported; OPG, orthopantomogram; PA, periapical radiograph; pos, position; Pract, private practice; pres, presence; radio, radiographical; rNRS, retrospective non-randomized study; rot, rotation; Uni, university.

*From the overall study sample, including also patients receiving treatment.

^§^On tooth level.

^†^Reported as mean (single value) and range (values in brackets).

### Risk of bias of included studies

The risk of bias of included studies was assessed with the Joanna Briggs Institute’s tool for prevalence studies (Table 2). The most problematic issue (apart from the retrospective design of almost all studies) was the inclusion of multiple teeth per patient without being analyzed appropriately to account for within-patient clustering, which was done in only three studies [36, 39, 40]. Moreover, few studies (36%; 5/14) accounted statistically for potential confounders that could influence spontaneous space closure [32, 35, 39–41]. Problematic were also the small response rates (36%; 5/14) and the inclusion of small samples (47%; 7/15).

**Table 2.** Internal validity assessment of included studies with the Joanna Briggs tool for prevalence studies.

Study	Prosp-ective?	Patients sampled appropriately?	Sample adequate (>100 teeth)?	Patients/setting described in detail?	Patient sample representative?	Space closure appropriately reported?	Follow-up adequate?	Appropriate data analysis (clustering)?	Appropriate data analysis (confounding)?	Response rate adequate? (>75%)
Aldahool 2024	**No**	**Yes**	**Yes**	**Yes**	**Partially**	**Yes**	**Yes**	**Yes**	**No**	**Yes**
Brusevold 2022	**No**	**Yes**	**No**	**Partially**	**Unclear**	**Yes**	**Yes**	**No**	**No**	**Unclear**
Canpolat 2020	**No**	**Yes**	**No**	**Partially**	**Yes**	**Yes**	**Yes**	**No**	**No**	**Yes**
Ciftci 2021	**No**	**Yes**	**Yes**	**Yes**	**Yes**	**Yes**	**Yes**	**No**	**Partially**	**Yes**
Ertugrul 2022	**No**	**Yes**	**No**	**Yes**	**Yes>**	**Yes**	**Yes**	**Yes**	**No**	**Unclear**
Gaudreau 2022	**Yes**	**Yes**	**No**	**Yes**	**Yes**	**Partially**	**Unclear**	**No**	**Yes**	**No**
Jalevik 2007	**No**	**Yes**	**No**	**Yes**	**Yes**	**No**	**Yes**	**No**	**No**	**Yes**
Lenaker 2022; 2023	**No**	**Yes**	**Yes**	**Yes**	**Partially**	**Yes**	**Yes**	**No**	**Yes**	**No**
Mouroutsou 2018	**No**	**Yes**	**Yes**	**Yes**	**Yes**	**Yes**	**Yes**	**No**	**No**	**No**
Nordeen 2022	**No**	**Yes**	**Yes**	**Yes**	**Yes**	**Yes**	**Yes**	**Yes**	**Yes**	**Yes**
Patel 2017	**No**	**Yes**	**Yes**	**Yes**	**Yes**	**Partially**	**Yes**	**Yes**	**Yes**	**Yes**
Rahhal 2014	**No**	**Unclear**	**No**	**Partially**	**Unclear**	**Yes**	**Unclear**	**No**	**No**	**Unclear**
Serindere 2019	**No**	**Yes**	**No**	**Yes**	**Yes**	**No**	**Yes**	**No**	**No**	**No**
Teo 2013	**No**	**No**	**Yes**	**Partially**	**Unclear**	**Yes**	**Yes**	**No**	**No**	**No**
Thilander 1970	**No**	**Yes**	**Yes**	**Partially**	**Unclear**	**Yes**	**Unclear**	**No**	**No**	**Yes**
Issues identified	93%	7%	47%	33%	13%	27%	0%	73%	73%	33%

### Data synthesis

The results of the raw dataset provided for two included studies are given in Appendices 5-7. Data synthesis is given in terms of one-group meta-analyses of pooled prevalences (Table 3) or pairwise two-group meta-analyses of ORs (Table 4), while the results of the remaining single studies that could not be meta-analyzed are shown in Supplementary Appendix 8.

**Table 3.** Indirect meta-analyses of average proportions among included studies.

Outcome	Studies	Teeth	Prevalence (95% CI)	τ^2^	*I* ^2^ (95% CI)
Spontaneous space closure (overall)	13	1787	61.6% (50.2%, 71.9%)	0.53	91% (86%, 94%)
Spontaneous space closure (maxilla)	9	665	85.3% (73.7%, 92.3%)	0.69	83% (70%, 91%)
Spontaneous space closure (mandible)	11	1095	48.1% (34.5%, 62.0%)	0.63	88% (80%, 93%)
2nd permanent molar rotation (mandible)	2	108	76.9% (15.5%, 98.4%)	0	27% (NC)
1st premolar rotation (mandible)	2	109	37.8% (0.7%, 98.2%)	0.17	83% (29%, 96%)
2nd premolar rotation (mandible)	2	105	60.0% (10.7%, 95.0%)	0	0% (NC)

CI, confidence interval; NC, non-calculable.

**Table 4.** Direct meta-analyses assessing the effect of various factors on spontaneous space closure.

Comparison	Studies	OR (95% CI)	P	τ^2^ (95% CI)	I^2^ (95% CI)	Prediction
*Overall*						
Maxilla versus mandible	9	7.77 (4.99, 12.11)	<0.001	0.08 (0, 1.63)	34% (0%, 69%)	3.39, 17.85
*Maxilla*						
Age 8-10 versus > 10 years	3	>100.00 (0.01, >100.00)	0.26	80.78 (21.41, >100.00)	100% (99%, 100%)	<0.01, >100.00
M2 Demirjian stages A–D versus F–H	3	>100.00 (0.02, >100.00)	0.26	71.33 (18.59, >100.00)	99% (9%, 100%)	<0.01, >100.00
M2 Demirjian stage E versus F-H	3	4.78 (1.56, 14.66)	0.006	0 (0, 11.92)	0% (0%, 90%)	<0.01, >100.00
M2 Demirjian stage F–G versus E–D	3	>100.00 (0.01, >100.00)	0.31	73.75 (19.04, >100.00)	99% (98%, 99%)	<0.01, >100.00
*Mandible*						
Age < 8 years versus > 10 years	2	5.01 (1.45, 17.30)	0.01	0 (NC)	0% (NC)	NC
Age 8-10 versus > 10 years	3	3.32 (1.73, 6.36)	<0.001	0 (0, 10.95)	0% (0%, 90%)	0.05, >100.00
Ages 11–12 versus 8–10 year	2	0.82 (0.35, 1.88)	0.63	0 (NC)	0% (NC)	NC
Ages 11–12 versus 8–10 year	2	0.29 (0.11, 0.81)	0.01	0 (NC)	0% (NC)	NC
Mean age (per year)	3	0.59 (0.20, >100.00)	0.35	0.83 (0.12, 40.37)	86% (59%, 95%)	<0.01, >100.00
Male versus female	2	1.38 (0.37, 5.14)	0.63	0.64 (NC)	69% (NC)	NC
M2 Demirjian stages A–D versus F–H	3	3.11 (1.25, 7.72)	0.01	0 (0, 8.30)	0% (0%, 90%)	<0.01, >100.00
M2 Demirjian stages A–D versus G–H	3	2.75 (0.50, 15.10)	0.24	1.23 (0, 59.78)	55% (0%, 87%)	<0.01, >100.00
M2 Demirjian stage E versus F–H	3	3.81 (1.85, 7.83)	<0.001	0 (0, 19.44)	0% (0%, 90%)	0.04, >100.00
M2 Demirjian stages E–F versus G–H	3	4.44 (0.43, 46.10)	0.21	3.66 (0.62, >100.00)	89% (69%, 96%)	<0.01, >100.00
M2 Demirjian stages F–G versus D–E	4	0.68 (0.28, 1.66)	0.26	0.04 (0, 4.23)	0% (0%, 85%)	0.14, 3.41
M2 distally inclined versus upright	3	1.18 (0.43, 3.21)	0.75	0.09 (0, 61.37)	28% (0%, 93%)	<0.01, >100
M2 mesially inclined versus upright	3	0.68 (0.27, 1.71)	0.41	0.39 (0, 32.90)	57% (0%, 88%)	<0.01, >100
M3 presence versus absence	4	2.28 (1.67, 3.09)	0.003	0 (0, 0.70	0% (0%, 85%)	0.79, 6.53

CI, confidence interval; M2, 2nd permanent molar; M3, 3rd permanent molar; NC, non-calculable; OR, odds ratio.

The provided datasets were used to identify confounders on jaw-differences for spontaneous space closure and estimate an adjusted-for-confounders estimate (Supplementary Appendices 5 and 7) to use in the pairwise meta-analyses. The provided dataset was also used to identify factors associated with spontaneous space closure after extraction of the permanent first molar, stratified by jaw (Supplementary Appendix 6). For the maxilla, data from the Aldahool et al. study [36] indicated that the odds for space closure were reduced with increased patient age (either as continuous variable or using cut-offs of 12 or 14 years of age) and with advanced developmental stage of the permanent second molar at the time of first molar extraction (with best outcomes seen for Demirjian stages A–D [OR over 100], followed by E–F [OR = 8.0] compared to G–H). For the mandible, data from the same study [36] indicated that patient age had a more pronounced effect on spontaneous space closure, with better results seen for the earliest extractions in the period of 8.2–15.8 years. The vertical position of the permanent second molar at the time of the first molar’s extraction was likewise associated with the odds of a subsequent spontaneous space closure, with lower (more apical) positions being associated with increased space closure odds. Finally, as far as developmental stage according to Demirjian are concerned, stages E–F showed the best space closure results (OR = 42.0), followed by stages A–D (OR = 9.3), and then stages G-H (reference).

**Figure 2.**
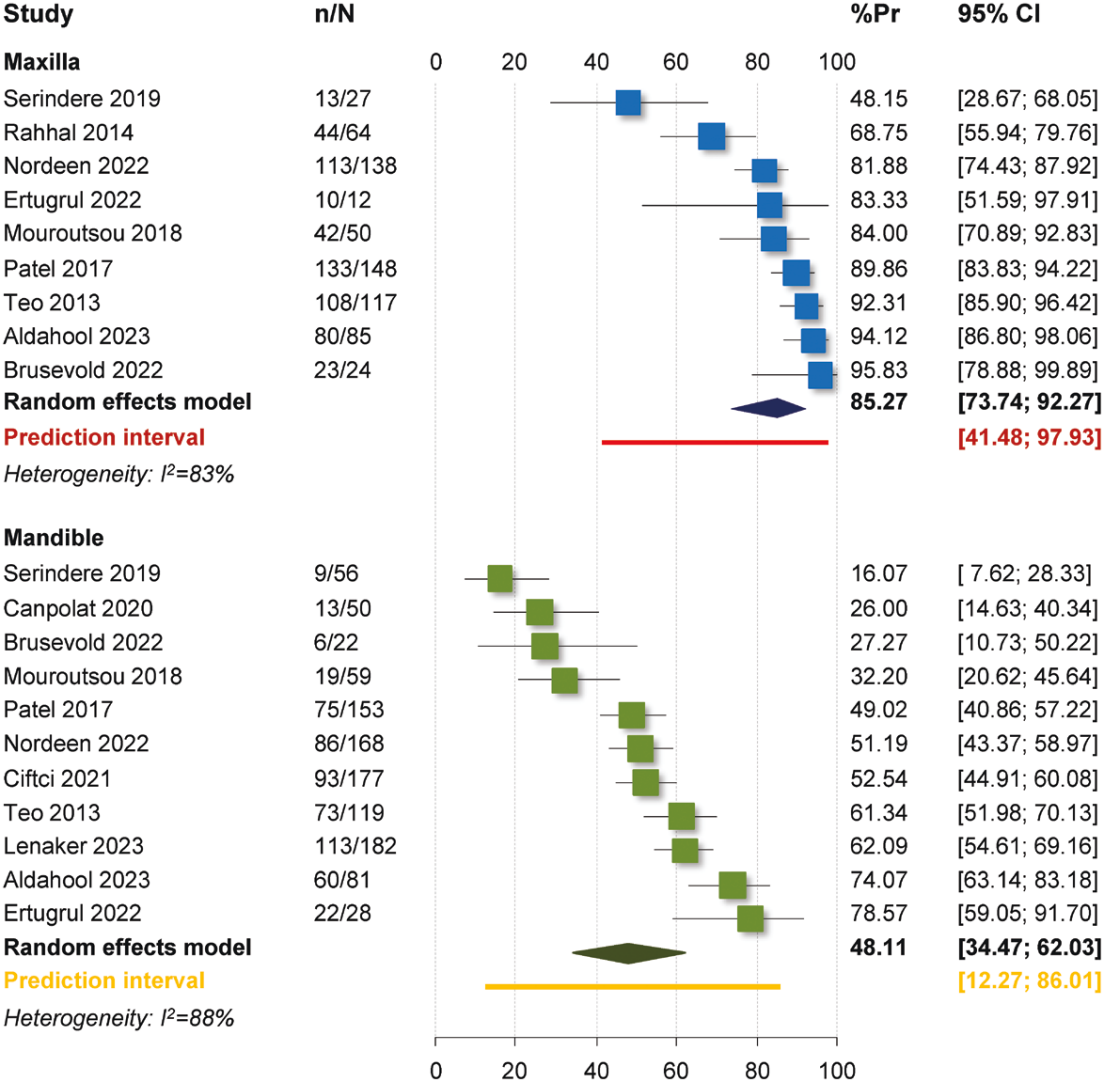
Forest plot for indirect meta-analysis on the average prevalence of spontaneous space closure after extraction of (a) maxillary or (b) mandibular permanent first molars. %Pr, per cent prevalence; CI, confidence interval; n/N, events/sample size.

From the 15 studies included in the qualitative synthesis, 14 were included in the meta-analyses, since one older study only partially reported data and precluded any further synthesis [42]. Single-group meta-analysis of prevalences indicated an overall rate of spontaneous space closure of 61.6% (13 studies; 95% CI 50.2%–71.9%), which differed considerably for the maxilla (pooled average 85.3%; 9 studies; 95% CI 73.7%–92.3%) and the mandible (pooled average 48.1%; 11 studies; 95% CI 34.5%–-62.0%; Table 3; Fig. 2). Additionally, based on the results of two studies, the pooled prevalences for rotations after extraction of mandibular first permanent molars were 76.9% for the mandibular second permanent molar (95% CI 15.5%–98.4%), 60.0% for the mandibular second premolar (95% CI 10.7%–95.0%), and 37.8% for the mandibular first premolar (95% CI 0.7%–-98.2%). However, very high heterogeneity was seen for almost all one-group pooled prevalences (*I*^2^ > 75%), which is to be expected, since several factors affected these outcomes.

**Figure 3.**
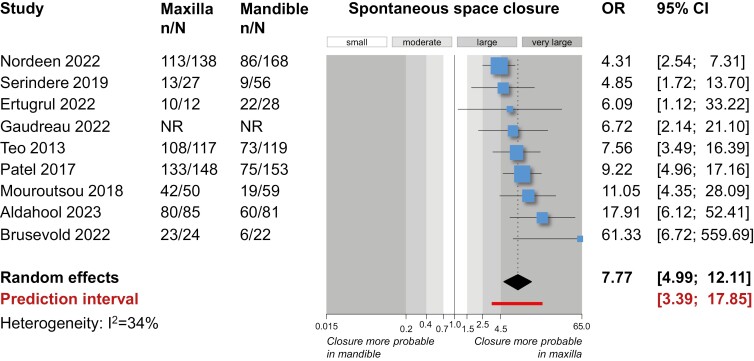
Forest plot for direct meta-analysis on prevalence of spontaneous space closure after extraction of permanent first molars in the maxilla versus the mandible. CI, confidence interval; n/N, events/sample size; NR, not reported; OR, odds ratio.

Pairwise meta-analyses (Table 4) indicated that spontaneous space closure was seen significantly more often in the maxilla than the mandible (nine studies; OR = 7.77; 95% CI = 4.99–12.11; *P* < 0.001; Fig. 3) with low heterogeneity (*I*^2^ = 34%).

For the maxilla (Table 4), Demirjian stage E of the permanent second molar at the time of the first molar’s extraction was associated with improved space closure odds (3 studies; OR = 4.78; 95% CI = 1.56–14.66; *P* = 0.006) compared with later stages (F–H).

**Figure 4.**
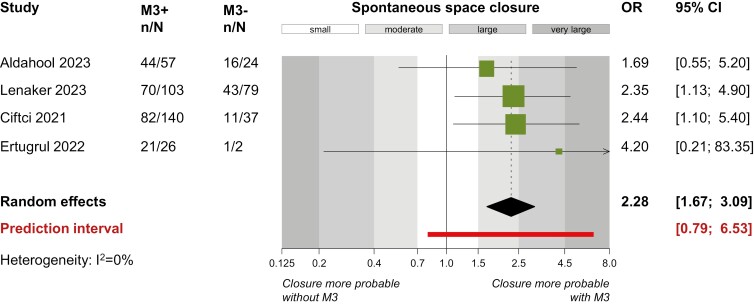
Forest plot for direct meta-analysis on prevalence of spontaneous space closure after extraction of mandibular permanent first molars in the presence versus the absence of third molars. CI, confidence interval; M3+/−; presence/absence of the third molar; n/N, events/sample; OR, odds ratio.

For the mandible (Table 4), earlier patient ages were associated with improved spontaneous space closure outcomes. Compared to patients older than 10 years at the time of first permanent molar extraction, improved spontaneous space closure outcomes were seen both for patients aged 8–10 years (3 studies; OR = 3.32; 95% CI = 1.73–6.36; *P* < 0.001) and patients younger than 8 years (2 studies; OR = 5.01; 95% CI = 1.45–17.30; *P* = 0.01). Permanent second molars being in earlier developmental stages at the time of the permanent first molar’s extraction were associated with increased odds of spontaneous space closure. Compared to later Demirjian stages F–H, the best outcomes were seen for stage E (3 studies; OR = 3.81; 95% CI = 1.85–7.83; *P* < 0.001), followed by stages A–D (three studies; OR = 3.11; 95% CI = 1.25–7.72; *P* = 0.01). Additionally, the presence of the mandibular third molar was associated with increased odds of spontaneous space closure in the mandible (4 studies; OR = 2.28;95% CI = 1.67–3.09; *P* = 0.003; Fig. 4).

Data from individual studies (Supplementary Appendix 8) indicated that spontaneous space closure overall was seen more often when crowding existed compared with spacing (one study; OR = 5.9; 95% CI = 1.3–27.7; *P* = 0.025). For the mandible, Nolla developmental stages 6–7 of the permanent second molar were associated with improved space closure outcomes (1 study; OR = 55.1; 95% CI = 3.0-at least 100.0; *P* = 0.007). Finally, midline deviations of the mandibular arch were seen more often when the permanent first molar was extracted only on one side (one study; OR 11.6; 95% CI = 1.9–70.3; *P* = 0.008).

### Additional analyses

Meta-regressions of pooled prevalence of space closure in either jaw did not find a significant effect of patient age or sex (Supplementary Appendix 9). The certainty of evidence with the GRADE approach (Tables 5–7) was low in all instances due to the inclusion of nonrandomized studies and the existence of methodological limitations that could have introduced bias. No hints of reporting biases were seen in terms of funnel plot asymmetry (Supplementary Appendix 10), which was also confirmed by a nonsignificant Thompson’s test (*P* = 0.31). Sensitivity analysis according to most methodological issues could not be performed, as all identified studies had at least one issue. Sensitivity analysis according to publication type showed no significant hints of inflated effects from including gray literature (Supplementary Appendix 11).

**Table 5.** Summary of findings table according to the GRADE approach according to jaw.

	Anticipated absolute effects (95% CI)				
Outcome Studies (teeth)	Mandible^a^	Maxilla	Difference in experimental	Quality of the evidence (GRADE)^b^	What happens in the maxilla
Spontaneous space closure 9 studies (1349 teeth)	485/1000	880/1000	395 teeth more (340 to 434 more)	⊕⊕◯◯ low^c^ due to bias	More often space closure in the maxilla

Intervention: extraction of the permanent first molar prior to the emergence of the permanent second molar/population: children or adolescents with permanent first molars in extraction need due to deep caries or molar incisor hypomineralization/setting: university clinics and hospitals (Belgium, Canada, England, Norway, Sweden, Turkey, United States of America).

^a^Response in the control group is based on the response of included studies (or random-effects meta-analysis of the control response).

^b^Starts from "high".

^c^Downgraded by two levels for bias due to the inclusion of non-randomized studies with serious risk of bias.

CI, confidence interval; GRADE, Grading of Recommendations Assessment, Development and Evaluation.

**Table 6.** Summary of findings table according to the GRADE approach for the maxilla.

	Anticipated absolute effects (95% CI)				
Outcome Studies (teeth)	*Demirjian stage F-H* ^a^	*Demirjian stage E*	Difference in experimental	Quality of the evidence (GRADE)^b^	What happens with experimental
Spontaneous space closure 3 studies (252 teeth)	906/1000	979/1000	73 teeth more (32 to 87 more)	⊕⊕◯◯low^c^ due to bias	More often maxillary space closure in Demirjian stage E

Intervention: extraction of the permanent first molar prior to the emergence of the permanent second molar/population: children or adolescents with permanent first molars in extraction need due to deep caries or molar incisor hypomineralization/setting: university clinics and hospitals (Sweden, Turkey, United States of America).

^a^Response in the control group is based on the response of included studies (or random-effects meta-analysis of the control response).

^b^Starts from "high"

^c^Downgraded by two levels for bias due to the inclusion of non-randomized studies with serious risk of bias.

CI, confidence interval; GRADE, Grading of Recommendations Assessment, Development and Evaluation.

**Table 7.** Summary of findings table according to the GRADE approach for the mandible.

	Anticipated absolute effects (95% CI)				
Outcome Studies (teeth)	Reference^a^	Experimental	Difference in experimental	Quality of the evidence (GRADE)^b^	What happens with experimental
	Age >10 yrs	Age <8 yrs			
Spontaneous space closure 2 studies (at least 15 teeth)	692/1000	919/1000	226 teeth more (73 to 283 more)	⊕⊕◯◯low^c^ due to bias	More often mandibular space closure in patients aged < 8yrs
	Age >10 yrs	Age 8-10 yrs			
Spontaneous space closure 3 studies (at least 109 teeth)	712/1000	891/1000	179 teeth more (98 to 252 more)	⊕⊕◯◯low^c^ due to bias	More often mandibular space closure in patients aged 8-10 yrs
	*Demirjian stages F–H*	*Demirjian stages A–D*			
Spontaneous space closure 3 studies (95 teeth)	650/1000	852/1000	202 teeth more (49 to 285 more)	⊕⊕◯◯low^c^ due to bias	More often mandibular space closure in Demirjian stage A-D
	*Demirjian stages F–H*	*Demirjian stage E*			
Spontaneous space closure 3 studies (at least 94 teeth)	650/1000	876/1000	226 teeth more (125 to 286 more)	⊕⊕◯◯lowc due to bias	More often mandibular space closure in Demirjian stage E
	*Third molar absent*	*Third molar present*			
Spontaneous space closure 4 studies (467 teeth)	497/1000	692/1000	195 teeth more (126 to 256 more)	⊕⊕◯◯low^c^ due to bias	More often mandibular space closure if third molar present

Intervention: extraction of the permanent first molar prior to the emergence of the permanent second molar/population: children or adolescents with permanent first molars in extraction need due to deep caries or molar incisor hypomineralization/setting: university clinics and hospitals (Sweden, Turkey, United States of America).

^a^Response in the control group is based on the response of included studies (or random-effects meta-analysis of the control response).

^b^Starts from "high".

^c^Downgraded by two levels for bias due to the inclusion of non-randomized studies with serious risk of bias.

CI, confidence interval; GRADE, Grading of Recommendations Assessment, Development and Evaluation; yr, year.

## Discussion

The present systematic review critically appraises and synthesizes existing studies on spontaneous space closure after early extraction of a permanent first molar in children and adolescents and identifies associated factors. Data from 15 studies, 1159 patients, and 2310 extraction quadrants indicated that spontaneous space closure occurs in 61.6% of the cases.

Spontaneous space closure without any additional treatment occurred significantly more often in the maxilla (85.3%) than in the mandible (48.1%). Possible explanations for this include the different eruptive paths of the upper and lower second molars. In the maxilla, the roots’ apices of the permanent second molar are positioned mesially in relation to the crown, which then tilt straight forward into a satisfactory position in the arch. On the other hand, the roots’ apices of the lower permanent second molar are placed distally and hence the crown tends to tip further mesially as it drifts forward [43]. The extraction of the lower permanent first molar prior to eruption of the permanent second molar might prevent this and leads to a bodily drift forward of the second molar through the bone with little tilting [43]. Additionally, the maxillary bone has in general lower mineral density than the mandibular bone [44] and mineral density is believed to be inversely associated with the rate of tooth movement [45–48], even though evidence for this is weak. This observed more favorable outcome in the maxilla also led to the assumption that the timing of the permanent first molar extraction is more critical in the mandible than in the maxilla [49].

Data from small meta-analyses (Table 4) indicated that significantly better spontaneous space closure outcomes in children and adolescents were observed for both the maxilla and the mandible, when the permanent second molars are in Demirjian stage E compared with second molars in other root development stages at the time of extraction. Re-analysis of individual patient data provided by a single study allowed more extensive explorative analysis (Supplementary Appendix 6). In the maxilla, acceptable spontaneous space closure results could be seen for most Demirjian stages and only Demirjian stage H (complete root formation) of the permanent second molar at the time of extraction was associated with worse outcomes. In the mandible, Demirjian stage E of the permanent second molar at the time of the first molar’s extraction was associated with the best spontaneous space closure outcomes and both prior (stage D) and, especially, latter developmental stages (stages F–H) led to reduced rates of spontaneous space closure. It is believed that pre-emergence eruptive movement begins with root formation, propelling the erupting tooth away from the point of root development [50]. It seems that a tooth’s pre-emergence eruption path is not necessarily determined genetically but can be affected by physical obstacles [51], the absence of potential guiding structures [52], and limited or excessive available space [53]. Current consensus related to the permanent first molar’s extraction timing indicates that this should always take place before the permanent second molar’s eruption [54], but this review provides more specific information related to tooth developmental stage.

Limited data from one study [32] indicated that spontaneous space closure might be expected more often when crowding exists, compared to other dental conditions. This is in agreement with earlier notions that favorable outcomes are seen for skeletal Class I cases with crowding [55] and with authors propagating that the decision to extract the permanent first molar should also take into consideration additional issues like the existence of hypodontia or other dental anomalies and crowding [56]. Possible explanations for this include the notion that crowded buccal segments might utilize the space made available from the first molar’s extraction to alleviate the crowding [57].

The presence of the mandibular permanent third molars was associated with considerable better spontaneous space closure outcomes compared to third molar agenesis cases. This is logical to expect, since the presence or absence of a particular mandibular molar might affect the eruption chances of the remaining molars—especially in cases with limited posterior dental arch spaces. Extraction of the permanent first molar will significantly increase the available space for the eruption of the third molars (thereby reducing their impaction risk) and improve their angulation [58]. Extraction of the first permanent molar has been shown to accelerate the development of the third permanent molar and increase its odds for mesial displacement [59, 60]. This indicates that prescribing a panoramic radiograph and the close cooperation between the general dentist, the pediatric dentist, and the orthodontist can be beneficial in improving treatment outcomes, while minimizing the burden for the patient [54]. However, it must be noted that calcification of the mandibular third molar crown start at 7–10 years of age and is usually completed between 12 and 16 years of age [61]. Therefore, the presence or the absence of the mandibular permanent third molar cannot always be confirmed at the time extraction decisions have to be made [9].

### Strengths and limitations

The strengths of the present review include its extensive literature search covering also gray literature [62], adherence to contemporary guidelines, use of improved statistical methods for data synthesis [25, 26], and the inclusion of re-analyzed raw data from one study [36].

However, several limitations of this study warrant consideration. The majority of the included studies were of retrospective design and had internal validity issues (Table 2), and therefore carry a risk of bias and further hamper causal inference [63]. Additionally, the heterogeneity observed across studies may influence the generalizability of findings and necessitates cautious interpretation. Furthermore, only few studies properly addressed statistically within-patient clustering of multiple molars [64] and attempts to procure individual patient data that could enhance data yield and precision [65] were unsuccessful (Supplementary Appendix 3) since the corresponding authors did not respond. One should also keep in mind that the acquisition of the pre-extraction radiographs used in included studies might deviate from the extraction timing of the first permanent molar and therefore, the developmental stage of the second permanent molar used in the analysis might not be completely accurate. Moreover, the average follow-up was only 4.0 years and adding that to the mean baseline patient age gives a mean patient age at outcome evaluation ranging from 12.2 to 15.1 years. This means that the possibility exists that for some cases the second permanent molar might not have fully erupted at the final examination. Finally, many studies included small samples of less than 100 teeth and this could influence the meta-analytical estimates [66]. Future research should focus on employing prospective designs with larger sample sizes (arbitrarily set to at least 100 teeth), standardized outcome measurement (including eruption success of the second permanent molar, residual spaces, rotations, tipping, supereruption of the antagonist teeth and need for further treatment), and appropriate statistical methods to provide more robust evidence.

## Conclusions

Existing evidence indicates that spontaneous space closure after early extraction of the permanent first molar in children and adolescents is seen more often in the maxilla than the mandible. Extraction of the mandibular permanent first molar before 10 years of age and at the Demirjian stage E of the mandibular second molar and the presence of the mandibular lower third molar is associated with increased odds of mandibular spontaneous space closure, while rotations of the mandibular molar and premolars are often seen. However, great uncertainty persists, due to the increased risk of bias of existing studies.

## Supplementary Material

cjae054_suppl_Supplementary_Material

## Data Availability

The study’s dataset is openly available through Zenodo (doi: 10.5281/zenodo.13717571).

## Registration

PROSPERO (CRD42023395371).
