# Supplementary material for: Spontaneous space closure after extraction of permanent first molars in children and adolescents: a systematic review and meta-analysis
Source: Eur J Orthod. 2024 Oct 9;46(6):cjae054. doi: 10.1093/ejo/cjae054 (PMC11461913; doi:10.1093/ejo/cjae054)
Supplement: cjae054_suppl_Supplementary_Material [file cjae054_suppl_supplementary_material.pdf]

Spontaneous space closure after extraction of permanent first molars in children and adolescents: a systematic review and meta-analysis

Supplementary material

Appendix 1. Additional review details and deviations from the protocol.

Additional method details

- Results of pairwise meta-analyses were presented in contour-enhanced forest plots illustrating the magnitude of observed effects [Papageorgiou, 2014]. This helps to assess the precision, heterogeneity, and clinical relevance of observed effects. For odds ratios, effects of 1.5, 2.5, and 4.5 (or inversely, 0.7, 0.4, and 0.2) were used as cut-off points to denote small, moderate, large, and very large effects.
- Individual patient data provided by the corresponding authors of included studies were re-analyzed with generalized linear models for the binomial family using odds ratios and their 95% confidence intervals. Clustering of multiple teeth within a patient was taken into account using robust standard errors. Potential confounders for the effect of jaw were identified using the change-in-estimate method and a 10% cut-off.

Deviations from protocol

- Initially, the risk of bias of included studies was to be assessed using the Cochrane Collaboration's Risk of Bias 2.0 tool and the ROBIN-I tool. As however, included studies are used to calculate the prevalence of spontaneous space closure, the Joanna Briggs Institute's tool for prevalence studies was used.
- Initially, the relative risk was planned to be used for pairwise meta-analyses, but this was ultimately changed to odds ratio due to favorable statistical properties.
- Mean differences were planned to be used for pairwise meta-analyses of continuous outcomes, but no such analyses were ultimately possible.
- Initially, a bootstrapped model was planned to be used for the meta-analysis of proportions, but this was changed to a random intercept logistic regression model, due to improved performance.

- Initially, the Knapp-Hartung adjustment was not planned, but this was incorporated for meta-analysis with >3 studies due to improved performance.
- Initially, the Egger's test was planned to be used to detect funnel plot asymmetry and hints of reporting biases. This was changed to Thompson's test, due to improved performance.
- Further additional analyses (according to ethnicity and stage of tooth development) were planned to be performed but could ultimately not be realized due to limited data.

References for Appendix 1

Papageorgiou SN. Meta-analysis for orthodontists: Part II--Is all that glitters gold? *J Orthod* 2014;**41**:327–36.

Appendix 2. Literature strategies and searches performed in each database.

Database	Search Strategy	Filters	Hits
MEDLINE (through PubMed)	("permanent molar" OR "permanent first molar" OR "first permanent molar" OR FPM) AND (extract* OR "loss")	Humans	387
Scopus	Same as PubMed	Subject area: Dentistry; Keywords: Human; Humans	592
Embase	Same as PubMed	-	102
Web of Science	Same as PubMed	Dentistry Oral Surgery Medicine	186
Virtual Health Library	Same as PubMed		92
**Sum with overlap**			1359
**Sum without overlap**			963
Last search date: 01.02.2024			

Appendix 3. Communications with corresponding authors of included studies to request individual patient data (or adjusted-for-confounders estimates).

Study	Request sent	Response
Aldahool 2024	**Yes**	**Sent full dataset**
Brusevold 2022	**Yes**	**No response**
Canpolat 2020	-	-
Ciftci 2021	-	-
Ertugrul 2022	-	-
Gaudreau 2022	-	-
Jalevik 2007	-	-
Lenaker 2022; 2023	**Yes**	**No response**
Mouroutsou 2018	-	-
Nordeen 2022	**Yes**	**No response**
Patel 2017	**Yes**	**No response**
Rahhal 2014	-	-
Serindere 2019	-	-
Teo 2013	**Yes**	**No response**
Thilander 1970	-	-

Appendix 4. Reports identified from the literature search with their inclusion / exclusion status.

Nr	Paper	Status
1	[No authors]. [Experts' consensus on ectopic eruption of the maxillary permanent first molar]. Zhonghua Kou Qiang Yi Xue Za Zhi. 2022;57(3):213-9.	Excluded by title
2	Abarca J, Zaror C, Monardes H, Hermosilla V, Muñoz C, Cantin M. Morphology of the physiological apical foramen in maxillary and mandibular first molars. Int j morphol. 2014;32(2):671-7.	Excluded by title
3	Åberg CH, Kwamin F, Claesson R, Johansson A, Haubek D. Presence of JP2 and non-JP2 genotypes of aggregatibacter actinomycetemcomitans and attachment loss in adolescents in ghana. Journal of Periodontology. 2012;83(12):1520-8.	Excluded by title
4	Aboujaoude S, Noueiri B, Berbari R, Khairalla A, Sfeir E. Evaluation of a modified Pulpotec endodontic approach on necrotic primary molars: a one-year follow-up. Eur J Paediatr Dent. 2015;16(2):111-4.	Excluded by title
5	Aboushala A, Kugel G, Hurley E. Class II composite resin restorations using glass-ionomer liners: Microleakage studies. Journal of Clinical Pediatric Dentistry. 1996;21(1):67-70.	Excluded by title
6	Abt E. Defective composite restorations--repair or replace? Evid Based Dent. 2014;15(2):52-3.	Excluded by title
7	Acosta-Rangel MC, Barrera-Vera H, Franco-Ornelas S, Aldape-Barrios B, Castro-García F. [Dentigerous cyst in a pediatric patient with acute lymphoblastic leukemia type 1]. Rev Med Inst Mex Seguro Soc. 2014;52(3):338-41.	Excluded by title
8	Adamowicz P, Gieroń J. 3-fluorophenmetrazine – A new psychoactive substance on the polish drug market. Z Zagadnien Nauk Sadowych. 2016;105:418-27.	Excluded by title
9	Adeboye SO, Cole BO, Jepson NJ, Hodgkinson PD. Multidisciplinary management of Blepharo-Cheilo-Dontic Syndrome and the role of overdenture in dental management. European journal of paediatric dentistry : official journal of European Academy of Paediatric Dentistry. 2009;10(4):201-4.	Excluded by title
10	Adewakun AA, Percival TM, Barclay SR, Amaechi BT. Caries status of children in eastern Trinidad, West Indies. Oral Health Prev Dent. 2005;3(4):249-61.	Excluded by title
11	Agarwal R, Chaudhry K, Yeluri R, Singh C, Munshi AK. Alternative approach to management of early loss of second primary molar: a clinical case report. J Calif Dent Assoc. 2014;42(5):327-30.	Excluded by title
12	Aggarwal VP, Mathur A, Mathur A. A 1-year appraisal of pit and fissure sealants following disinfection with and without chlorhexidine solution: An in vivo randomized trial. J Indian Soc Pedod Prev Dent. 2018;36(4):402-6.	Excluded by title
13	Agnihotry A, Fedorowicz Z, Nasser M. Adhesively bonded versus non-bonded amalgam restorations for dental caries. Cochrane Database Syst Rev. 2016;3(3):Cd007517.	Excluded by title
14	Ahovuo-Saloranta A, Forss H, Hiiri A, Nordblad A, Mäkelä M. Pit and fissure sealants versus fluoride varnishes for preventing dental decay in the permanent teeth of children and adolescents. Cochrane Database Syst Rev. 2016;2016(1):Cd003067.	Excluded by title
15	Ahovuo-Saloranta A, Forss H, Walsh T, Hiiri A, Nordblad A, Mäkelä M, et al. Sealants for preventing dental decay in the permanent teeth. Cochrane Database Syst Rev. 2013(3):Cd001830.	Excluded by title
16	Ahovuo-Saloranta A, Forss H, Walsh T, Nordblad A, Mäkelä M, Worthington HV. Pit and fissure sealants for preventing dental decay in permanent teeth. Cochrane Database Syst Rev. 2017;7(7):Cd001830.	Excluded by title
17	Aktan AM, Cebe MA, Ciftci ME, Karaarslan ES. A novel LED-based device for occlusal caries detection. Lasers in Medical Science. 2012;27(6):1157-63.	Excluded by title
18	Al Maaitah EF, Adeyemi AA, Higham SM, Pender N, Harrison JE. Factors affecting demineralization during orthodontic treatment: A post-hoc analysis of RCT recruits. American Journal of Orthodontics and Dentofacial Orthopedics. 2011;139(2):181-91.	Excluded by title
19	Al Mheiri E, Chaudhry J, Abdo S, El Abed R, Khamis AH, Jamal M. Evaluation of root and canal morphology of maxillary permanent first molars in an Emirati population; A cone-beam computed tomography study. BMC Oral Health. 2020;20(1).	Excluded by title
20	Al Shalabi RM, Omer OE, Glennon J, Jennings M, Claffey NM. Root canal anatomy of maxillary first and second permanent molars. International Endodontic Journal. 2000;33(5):405-14.	Excluded by title
21	Alaçam T, Tinaz AC, Genç Ö, Kayaoglu G. Second mesiobuccal canal detection in maxillary first molars using microscopy and ultrasonics. Australian Endodontic Journal. 2008;34(3):106-9.	Excluded by title
22	Albani F, Ballesio I, Campanella V, Marzo G. Pit and fissure sealants: results at five and ten years. European journal of paediatric dentistry. 2005;6(2):61-5.	Excluded by title
23	Alcântara CE, Corrêa-Faria P, Tavano KT, Botelho AM. Fragment reattachment associated with pulpotomy in a posterior tooth with incomplete root formation. Eur J Prosthodont Restor Dent. 2011;19(4):155-9.	Excluded by title
24	Alekhya KLNV, Kadakampally D. Reccurent peripheral giant cell granuloma: A case report. Dental and Medical Problems. 2017;54(1):97-100.	Excluded by title
25	Alexander SA, Askari M, Lewis P. The premature loss of primary first molars: space loss to molar occlusal relationships and facial patterns. Angle Orthod. 2015;85(2):218-23.	Excluded by title
26	Alharbi F, Almuzian M, Bearn D. Anchorage effectiveness of orthodontic miniscrews compared to headgear and transpalatal arches: a systematic review and meta-analysis. Acta Odontol Scand. 2019;77(2):88-98.	Excluded by title
27	Ali A, Abbas M, Saeed U. A rare cause of stroke in young: Moyamoya disease. Postgraduate Medical Journal. 2020;96(1132):119.	Excluded by title
28	Alkhadra T. Characteristic of Malocclusion among Saudi Special Need Group Children. J Contemp Dent Pract. 2017;18(10):959-63.	Excluded by title
29	Alkilzy M, Tarabaih A, Santamaria RM, Splieth CH. Self-assembling Peptide P11-4 and Fluoride for Regenerating Enamel. Journal of Dental Research. 2018;97(2):148-54.	Excluded by title
30	Alm A, Wendt LK, Koch G. Dental treatment in the primary dentition of 7-12 year-old Swedish schoolchildren. Swedish Dental Journal. 2003;27(2):77-82.	Excluded by title
31	Al-Manei KK, Owaiwid AB, Dhafiri RA, Al-Manei K, Harran SA, Alsulaimani RS. Shear bond strength of E. Max ceramic restoration to hydraulic calcium silicate based cement (Biodentine): An in vitro study. European Endodontic Journal. 2020;5(3):288-94.	Excluded by title
32	Almuammar MF, Schulman A, Salama FS. Shear bond strength of six restorative materials. Journal of Clinical Pediatric Dentistry. 2001;25(3):221-5.	Excluded by title
33	Alnahwi HH, Donly KJ, Contreras CI. Space loss following premature loss of primary second molars. Gen Dent. 2015;63(6):e1-4.	Excluded by title
34	Alovisi M, Cemenasco A, Mancini L, Paolino D, Scotti N, Bianchi CC, et al. Micro-CT evaluation of several glide path techniques and ProTaper Next shaping outcomes in maxillary first molar curved canals. International Endodontic Journal. 2017;50(4):387-97.	Excluded by title
35	Alqaderi HE, Al-Mutawa SA, Qudeimat MA. MTA pulpotomy as an alternative to root canal treatment in children's permanent teeth in a dental public health setting. Journal of Dentistry. 2014;42(11):1390-5.	Excluded by title
36	Al-Qudah AA, Awawdeh LA. Root and canal morphology of mandibular first and second molar teeth in a Jordanian population. Int Endod J. 2009;42(9):775-84.	Excluded by title
37	Alsabek L, Al-Nerabieah Z, Bshara N, Comisi JC. Retention and remineralization effect of moisture tolerant resin-based sealant and glass ionomer sealant on non-cavitated pit and fissure caries: Randomized controlled clinical trial. Journal of Dentistry. 2019;86:69-74.	Excluded by title
38	Alsamahi S, Milne TJ, Hussaini HMM, Rich AM, Cooper PR, Friedlander LT. Effects of glycation end-products on the dental pulp in patients with type 2 diabetes. International Endodontic Journal. 2023;56(11):1373-84.	Excluded by title
39	Alsamahi S, Milne TM, Hussaini H, Rich AM, Friedlander LT. Type 2 diabetes and the clinically normal pulp: An in vitro study. Int Endod J. 2022;55(6):660-71.	Excluded by title
40	Al-Sarheed M. Bond strength of 4 sealants using conventional etch and a self-etching primer. Journal of Dentistry for Children. 2006;73(1):37-41.	Excluded by title
41	Al-Sarheed MA. Evaluation of shear bond strength and SEM observation of all-in-one self-etching primer used for bonding of fissure sealants. Journal of Contemporary Dental Practice. 2006;7(2):9-16.	Excluded by title
42	AlSunbul H, Murriky A. Efficacy of methylene blue and curcumin mediated antimicrobial photodynamic therapy in the treatment of indirect pulp capping in permanent molar teeth. Photodiagnosis Photodyn Ther. 2023;42:103598.	Excluded by title
43	Altinok B, Tanboga I, Peker S, Eren F, Bakkal M, Peker F. The effect of laseractivated Acidulated Phosphate Fluoride on enamel submitted to erosive solution only: An in vitro preliminary evaluation. European Journal of Paediatric Dentistry. 2011;12(1):13-6.	Excluded by title
44	Al-Turaihi BA, Alburgaiba MH, Ali IH. Interceptive orthodontics "space maintainers: When and what to use". Latin American Journal of Pharmacy. 2021;40(Special Issue):276-83.	Excluded by title
45	Amaechi BT, Alshareif DO, Azees PAA, Shehata MA, Lima PP, Abdollahi A, et al. Anti-caries evaluation of a nano-hydroxyapatite dental lotion for use after toothbrushing: An in situ study. J Dent. 2021;115:103863.	Excluded by title
46	Amižic IP, Munitic MS. Root resorption of maxillary permanent first molar caused by impacted second molar: A case report. Acta Stomatologica Croatica. 2017;51(4):371.	Excluded by title
47	Anand P, Wilson R, Sheehy EC. Intraligamental analgesia for post-operative pain control in children having dental extractions under general anaesthesia. European journal of paediatric dentistry. 2005;6(1):10-5.	Excluded by title
48	Ananda GK, Nambiar P, Mutalik S, Shanmuhasuntharam P. Anatomical considerations for implant placements in first maxillary molar extracted sites in East Asian patients. Surg Radiol Anat. 2015;37(9):1099-108.	Excluded by title
49	Anderson DL, Popovich F. Dental reductions and dental caries. Am J Phys Anthropol. 1977;47(3):381-5.	Excluded by title
50	Ando M, Eckert GJ, Stookey GK, Zero DT. Effect of imaging geometry on evaluating natural white-spot lesions using quantitative light-induced fluorescence. Caries Research. 2004;38(1):39-44.	Excluded by title
51	Andreani Y, Gad BT, Cocks TC, Harrison J, Keresztes ME, Pomfret JK, et al. Comparison of irrigant activation devices and conventional needle irrigation on smear layer and debris removal in curved canals. (Smear layer removal from irrigant activation using SEM). Australian Endodontic Journal. 2021;47(2):143-9.	Excluded by title
52	Angelopoulou MV, Koletsi D, Vadiakas G, Halazonetis DJ. Induced ankylosis of a primary molar for skeletal anchorage in the mandible as alternative to mini-implants. Prog Orthod. 2015;16:18.	Excluded by title
53	Anilkumar K, Lingeswaran S, Ari G, Thyagarajan R, Logaranjani A. Management of chronic hyperplastic pulpitis in mandibular molars of middle aged adults- a multidisciplinary approach. Journal of Clinical and Diagnostic Research. 2016;10(1):ZD23-ZD5.	Excluded by title
54	Annibali S, Pippi R, Perfetti G. Unusual surgical approach in a bilateral case of mandibular buccal infected cyst. Minerva stomatologica. 2002;51(5):219-24.	Excluded by title
55	Antolic I. [Characteristics of orofacial system in case of Slovene schoolchildren within four countries]. Zobozdrav Vestn. 1989;44(1-2):35-48.	Excluded by title
56	Archila Gallegos MdJ, Santamaría Hernández DA. Conocimientos y prácticas sobre prevención de caries dental asociados a prevalencia de pérdida de primera molar permanente en tres municipios de El Salvador, 2013. 2014. p. 36-.	Excluded by title
57	Ariyaratnam MT, Wilson MA, Blinkhorn AS. An analysis of surface roughness, surface morphology and composite/dentin bond strength of human dentin following the application of the Nd:YAG laser. Dent Mater. 1999;15(4):223-8.	Excluded by title
58	Ariyaratnam MT, Wilson MA, Mackie IC, Blinkhorn AS. A comparison of surface roughness and composite/enamel bond strength of human enamel following the application of the Nd:YAG laser and etching with phosphoric acid. Dent Mater. 1997;13(1):51-5.	Excluded by title
59	Arnold WH, Gaengler P, Saeuberlich E. Distribution and volumetric assessment of initial approximal caries lesions in human premolars and permanent molars using computer-aided three-dimensional reconstruction. Archives of Oral Biology. 2000;45(12):1065-71.	Excluded by title
60	Arora R, Goswami M, Chaudhary S, Chaitra TR, Kishor A, Rallan M. Comparative evaluation of effects of chemo-mechanical and conventional caries removal on dentinal morphology and its bonding characteristics - an SEM study. Eur Arch Paediatr Dent. 2012;13(4):179-84.	Excluded by title
61	Asgary S, Eghbal MJ, Ghoddusi J, Yazdani S. One-year results of vital pulp therapy in permanent molars with irreversible pulpitis: An ongoing multicenter, randomized, non-inferiority clinical trial. Clinical Oral Investigations. 2013;17(2):431-9.	Excluded by title
62	Asgary S, Eghbal MJ, Ghoddusi J. Two-year results of vital pulp therapy in permanent molars with irreversible pulpitis: an ongoing multicenter randomized clinical trial. Clin Oral Investig. 2014;18(2):635-41.	Excluded by title
63	Assaf AV, Meneghim MDC, Zanin L, Cortelazzi KL, Pereira AC, Ambrosano GMB. Effect of different diagnostic thresholds on dental caries calibration. Journal of Public Health Dentistry. 2006;66(1):17-22.	Excluded by title
64	Assunção CM, Falleiros T, Gugisch RC, Fraiz FC, Losso EM. Tetralogia de Fallot e sua repercussão na saúde bucal. Rev paul pediatr. 2008;26(1):93-6.	Excluded by title
65	Atieh MA. Tooth loss among Saudi adolescents: Social and behavioural risk factors. International Dental Journal. 2008;58(2):103-8.	Excluded by title
66	Aytutuldu GK, Ersöz Hüseyinsinoğlu B. The investigation of the relationship between the fall risk and the hand grip strength of individuals with Parkinson's disease: a pilot study. Turkish Journal of Physiotherapy and Rehabilitation. 2019;30(2):S34.	Excluded by title

67	Baart JA, Bosgra JF. [Eruption disturbances of the second molar in the upper jaw by the third molar]. Ned Tijdschr Tandheelkd. 2002;109(9):351-4.	Excluded by title
68	Babacan H, Ay S, Kösger HH. Impacted permanent first molars: two case reports. Int Dent J. 2006;56(1):49-54.	Excluded by title
69	Babaei A, Pakdaman A, Hessari H, Shamshiri AR. Oral health of 6-7 year-old children according to the Caries Assessment Spectrum and Treatment (CAST) index. BMC Oral Health. 2019;19(1).	Excluded by title
70	Bacić M, Sutalo J, Čuković I. Morphological characteristics of inter-radicular space of the first and second lower permanent molars. Acta stomatologica Croatica. 1988;22(4):271-8.	Excluded by title
71	Bacon J, Coates H, Gupta N. The benefits of an integrated eye clinic liaison officer in improving patient outcomes in ophthalmology. Postgraduate Medical Journal. 2018;94:A14-A5.	Excluded by title
72	Bagheri M, Pilecki P, Sauro S, Sherriff M, Watson TF, Hosey MT. An in vitro investigation of pre-treatment effects before fissure sealing. International Journal of Paediatric Dentistry. 2017;27(6):514-22.	Excluded by title
73	Baginska J, Rodakowska E, Milewski R, Kierklo A. Dental caries in primary and permanent molars in 7-8-year-old schoolchildren evaluated with Caries Assessment Spectrum and Treatment (CAST) index. BMC Oral Health. 2014;14(1).	Excluded by title
74	Bagramian RA, Graves RC, Srivastava S. Sealant effectiveness for children receiving a combination of preventive methods in a fluoridated community: two-year results. J Dent Res. 1977;56(12):1511-9.	Excluded by title
75	Bagramian RA, Srivastava S, Graves RC. Pattern of sealant retention in children receiving a combination of caries-preventive methods: three-year results. Journal of the American Dental Association (1939). 1979;98(1):46-50.	Excluded by title
76	Baker KL, Storey E. Tetracycline-induced tooth changes. 3. Incidence in extracted first permanent molar teeth. Med J Aust. 1970;1(3):109-13.	Excluded by title
77	Baker KL. Tetracycline-induced tooth changes. Part 5. Incidence in extracted first permanent molar teeth: a resurvey after four years. Med J Aust. 1975;2(8):301-4.	Excluded by title
78	Bakhshandeh A, Ekstrand KR, Qvist V. Measurement of histological and radiographic depth and width of occlusal caries lesions: A methodological study. Caries Research. 2011;45(6):547-55.	Excluded by title
79	Balevi B. Defective amalgam restorations--repair or replace? Evid Based Dent. 2014;15(2):54-5.	Excluded by title
80	Balmer R, Fayle SA. Enamel defects and ectopic eruption in a child with Usher syndrome and a cochlear implant. Int J Paediatr Dent. 2007;17(1):57-61.	Excluded by title
81	Balmer R, Toumba J, Godson J, Duggal M. The prevalence of molar incisor hypomineralisation in Northern England and its relationship to socioeconomic status and water fluoridation. International Journal of Paediatric Dentistry. 2012;22(4):250-7.	Excluded by title
82	Bandeira Ferreira Santos CdF, Godoy F, Menezes VA, Colares V, Zarzar PM, Ferreira RC, et al. School academic climate and oral health (tooth loss) in adolescents. Plos One. 2020;15(5).	Excluded by title
83	Barbería E, Lucavechi T, Cárdenas D, Maroto M. Free-end space maintainers: design, utilization and advantages. J Clin Pediatr Dent. 2006;31(1):5-8.	Excluded by title
84	Barberia-Leache E, Suarez-Clúa MC, Saavedra-Ontiveros D. Ectopic eruption of the maxillary first permanent molar: characteristics and occurrence in growing children. Angle Orthod. 2005;75(4):610-5.	Excluded by title
85	Barfod KW, Kastoft R, Bencke J, Speedtsberg M, Søndergaard RM, Penny JO. Achilles tendon length, ATRS and functional outcomes 5 years after acute achilles tendon rupture treated conservatively. Arthroscopy - Journal of Arthroscopic and Related Surgery. 2017;33(10):e128-e9.	Excluded by title
86	Barnard E, French W. The silent scream or the menopause. Postgraduate Medical Journal. 2020;96(1132):120.	Excluded by title
87	Barnard E, Tucker A, Mozaffari M, Teshima T. A dry and silent world: Hidden disabilities. Postgraduate Medical Journal. 2020;96(1132):120.	Excluded by title
88	Barnes PJ. Therapy for asthma and COPD. Postgraduate Medical Journal. 2018;94:A2-A3.	Excluded by title
89	Barros SE, Siqueira SP, Janson G, Chiqueto K. Short-term efficacy of vacuum-formed maintainer for deciduous second molar space maintenance in the mixed dentition: A single-centre, randomized controlled clinical trial. Orthod Craniofac Res. 2021;24(4):502-10.	Excluded by title
90	Barry S, Allotey J, Brundler AM, Duggal MS. Case report: cystic hygroma. Eur Arch Paediatr Dent. 2012;13(6):323-5.	Excluded by title
91	Bartle E, Urner T, Rao T, Kowalczyk AP, Mattheyses AL. Imaging nanoscale changes in desmosome protein organization. Journal of Investigative Dermatology. 2018;138(5):S149.	Excluded by title
92	Bataineh AB, Alwarafi MA. Patient's pain perception during mandibular molar extraction with articaine: a comparison study between infiltration and inferior alveolar nerve block. Clin Oral Investig. 2016;20(8):2241-50.	Excluded by title
93	Batista MJ, Rihs LB, de Sousa MLR. Risk indicators for tooth loss in adult workers. Brazilian Oral Research. 2012;26(5):390-6.	Excluded by title
94	Batstone MD, Macleod AWG. Oral and maxillofacial surgical considerations for a case of Hutchinson-Gilford progeria. International Journal of Paediatric Dentistry. 2002;12(6):429-32.	Excluded by title
95	Baysal A, Karadede I, Hekimoglu S, Ucar F, Ozer T, Veli I, et al. Evaluation of root resorption following rapid maxillary expansion using cone-beam computed tomography. Angle Orthodontist. 2012;82(3):488-94.	Excluded by title
96	Beal JF, James PMC. Dental Caries Prevalence in 5-Year-Old Children Following 5 1/2 Years of Water Fluoridation in Birmingham. British Dental Journal. 1971;130(7):284-&.	Excluded by title
97	Benazzi S, Bailey SE, Peresani M, Mannino MA, Romandini M, Richards MP, et al. Middle Paleolithic and Uluzzian human remains from Fumane Cave, Italy. J Hum Evol. 2014;70:61-8.	Excluded by title
98	Benn A, Altini M. Dentigerous cysts of inflammatory origin A clinicopathologic study. Oral Surgery, Oral Medicine, Oral Pathology, Oral Radiology, and Endodontics. 1996;81(2):203-9.	Excluded by title
99	Benson RE, Rodd HD, North S, Loescher AR, Farthing PM, Payne M. Leukaemic infiltration of the mandible in a young girl. Int J Paediatr Dent. 2007;17(2):145-50.	Excluded by title
100	Benteke M, Berntsson L, Broman U, Edfeldt K, Sköld-Larsson K, Twetman S. Population- vs.risk-based applications of fissure sealants in first permanent molars: A 13-year follow-up. Oral Health and Preventive Dentistry. 2006;4(2):151-6.	Excluded by title
101	Berdouses ED, Michalaki M, Tsinidou K, Vlachou A, Pantazis N, Oulis CJ. Effectiveness of fissure sealants on initial caries lesions (ICDAS 1-3) of permanent molars: A 4-year follow-up. European Journal of Paediatric Dentistry. 2021;22(3):180-8.	Excluded by title
102	Bereket C, Çakir-Özkan N, Şener I, Kara I, Aktan AM, Arici N. Retrospective analysis of impacted first and second permanent molars in the turkish population: A multicenter study. Medicina Oral, Patologia Oral y Cirugia Bucal. 2011;16(7):e874-e8.	Excluded by title
103	Bertasso AS, Léon JE, Silva RAB, Silva LAB, de Queiroz AM, Pucinelli CM, et al. Immunophenotypic quantification of M1 and M2 macrophage polarization in radicular cysts of primary and permanent teeth. International Endodontic Journal. 2020;53(5):627-35.	Excluded by title
104	Bhat PK, Navin HK, Idris M, Christopher P, Rai N. Modified distal shoe appliance for premature loss of multiple deciduous molars: A case report. Journal of Clinical and Diagnostic Research. 2014;8(8):ZD43-ZD5.	Excluded by title
105	Bhatnagar VK, Miller MH, Ketcheson JW. Reaction of fertilizer and liquid manure phosphorus with soil aggregates and sediment phosphorus enrichment. Journal of Environmental Quality. 1985;14(2):246-51.	Excluded by title
106	Bial JJ, Mellonig JT. Radiographic evaluation of juvenile periodontitis (periodontosis). Journal of periodontology. 1987;58(5):321-6.	Excluded by title
107	Bishara SE, Bayati P, Zaher AR, Jakobsen JR. Comparisons of the dental arch changes in patients with Class II, division 1 malocclusions: extraction vs nonextraction treatments. Angle Orthodontist. 1994;64(5):351-8.	Excluded by title
108	Bishara SE. Mandibular changes in persons with untreated and treated Class II division 1 malocclusion. American journal of orthodontics and dentofacial orthopedics : official publication of the American Association of Orthodontists, its constituent societies, and the American Board of Orthodontics. 1998;113(6):661-73.	Excluded by title
109	Bjerklin K, Al-Najjar M, Karestedt H, Andren A. Agenesis of mandibular second premolars with retained primary molars. A longitudinal radiographic study of 99 subjects from 12 years of age to adulthood. European Journal of Orthodontics. 2008;30(3):254-61.	Excluded by title
110	Blinkhorn AS, Hassall DC, Holloway PJ, Mellor AC, Worthington HV. An assessment of capitation in the new General Dental Service contract. Community Dental Health. 1996;13 SUPPL. 1:3-20.	Excluded by title
111	Bodner L, Goldstein J, Sarnat H. Eruption cysts: A clinical report of 24 new cases. Journal of Clinical Pediatric Dentistry. 2004;28(2):183-6.	Excluded by title
112	Bönecker M, Grossman E, Cleaton-Jones PE, Parak R. Clinical, histological and microbiological study of hand-excavated carious dentine in extracted permanent teeth. Sadj. 2003;58(7):273-8.	Excluded by title
113	Booij JW, Goeke J, Bronkhorst EM, Katsaros C, Ruf S. Class II treatment by extraction of maxillary first molars or Herbst appliance: dentoskeletal and soft tissue effects in comparison. J Orofac Orthop. 2013;74(1):52-63.	Excluded by title
114	Booij JW, Kuijpers-Jagtman AM, Bronkhorst EM, Livas C, Ren Y, Kuijpers MAR, et al. Class II Division 1 malocclusion treatment with extraction of maxillary first molars: Evaluation of treatment and post-treatment changes by the PAR Index. Orthod Craniofac Res. 2021;24(1):102-10.	Excluded by title
115	Børsting T, Schuller A, Van Dommelen P, Stafne SN, Slåttelid Skeie M, Skaare AB, et al. Associations between maternal vitamin D status in second and third trimester of pregnancy and offspring enamel hypomineralisation at 7-9 years: A longitudinal study. Norsk Epidemiologi. 2019;28:45.	Excluded by title
116	Borzabadi-Farahani A, Yen SLK, Francis C, Lara-Sanchez PA, Hammoudeh J. A rare case of accessory maxilla and bilateral Tessier no. 7 clefts, a 10-year follow-up. Journal of Cranio-Maxillofacial Surgery. 2013;41(6):527-31.	Excluded by title
117	Bossù M, Bartoli A, Orsini G, Luppino E, Polimeni A. Enamel hypoplasia in coeliac children: a potential clinical marker of early diagnosis. European journal of paediatric dentistry : official journal of European Academy of Paediatric Dentistry. 2007;8(1):31-7.	Excluded by title
118	Botelho AM, Tavano KTA, Correa-Faria P, Morato LNS, Viana MR. Esthetic-functional recovery of permanent posterior tooth using autogenous biological restoration. Journal of Indian Society of Pedodontics and Preventive Dentistry. 2012;30(4):333-6.	Excluded by title
119	Botton G, Morgental CS, Scherer MM, Lenzi TL, Montagner AF, Rocha RO. Are self-etch adhesive systems effective in the retention of occlusal sealants? A systematic review and meta-analysis. International journal of paediatric dentistry. 2016;26(6):402-11.	Excluded by title
120	Bravo M, Osorio E, García-Anllo I, Llodra JC, Baca P. The influence of dft index on sealant success: A 48-month survival analysis. Journal of Dental Research. 1996;75(2):768-74.	Excluded by title
121	Breakspear EK. Indications for extraction of the lower second permanent molar. Dent Pract Dent Rec. 1967;17(5):198-200.	Excluded by title
122	Bredy E, Erbring C, Reichel I. Frequency of Tooth Extraction in Orthodontic Treatment. Stomatologie der DDR. 1988;38(6):391-5.	Excluded by title
123	Bresciani L, Favari C, Calani L, Francinelli V, Riva A, Petrangolini G, et al. The Effect of Formulation of Curcuminoids on Their Metabolism by Human Colonic Microbiota. Molecules. 2020;25(4).	Excluded by title
124	Brill WA. The distal shoe space maintainer chairside fabrication and clinical performance. Pediatr Dent. 2002;24(6):561-5.	Excluded by title
125	Brocklehurst P, Price J, Glenny AM, Tickle M, Birch S, Mertz E, et al. The effect of different methods of remuneration on the behaviour of primary care dentists. Cochrane Database Syst Rev. 2013;2013(11):Cd009853.	Excluded by title
126	Brownbill JW, Setcos JC. Treatment selections for fissured grooves of permanent molar teeth. ASDC J Dent Child. 1990;57(4):274-8.	Excluded by title
127	Brunetto M, Da Silva Pereira Andriani J, Ribeiro GLU, Locks A, Correa M, Correa LR. Three-dimensional assessment of buccal alveolar bone after rapid and slow maxillary expansion: A clinical trial study. American Journal of Orthodontics and Dentofacial Orthopedics. 2013;143(5):633-44.	Excluded by title
128	Brunton PA, Kassir A, Dashti M, Setcos JC. Effect of Different Application and Polymerization Techniques on the Microleakage of Proximal Resin Composite Restorations In Vitro. Operative Dentistry. 2004;29(1):54-9.	Excluded by title
129	Brusevold IJ, Bie TMG, Baumgartner CS, Das R, Espelid I. Molar incisor malformation in six cases: description and diagnostic protocol. Oral Surgery, Oral Medicine, Oral Pathology and Oral Radiology. 2017;124(1):52-61.	Excluded by title
130	Buchanan GD, Tredoux S, Schouwstra CM, Nel C, Gamieldien MY. Double Gemination or Fusion of a Permanent Mandibular Second Molar: A Case Report. Chin J Dent Res. 2021;24(3):199-202.	Excluded by title
131	Burrow JF, Burrow MF, Makinson OF. Pits and fissures: Relative space contribution in fissures from sealants, prophylaxis pastes and organic remnants. Australian Dental Journal. 2003;48(3):175-9.	Excluded by title
132	Byrne CD. Diagnosis and management of non-alcoholic fatty liver disease (NAFLD). Postgraduate Medical Journal. 2018;94:A2.	Excluded by title

133	Calheiros-Lobo MJ, Costa F, Pinho T. Infraocclusion level and root resorption of the primary molar in second premolar agenesis: A retrospective cross-sectional study in the Portuguese population. Dent Med Probl. 2022;59(2):195-207.	Excluded by title
134	Campos M, González H, Bocaz G, Vásquez O. Active site peptide of beta-lactamase from Shigella flexneri UCSF-129. Microbios. 1997;90(362):17-22.	Excluded by title
135	Carlos JP, Wolfe MD, Zambon JJ, Kingman A. Periodontal Disease in Adolescents: Some Clinical and Microbiologic Correlates of Attachment Loss. Journal of Dental Research. 1988;67(12):1510-4.	Excluded by title
136	Carrington J, Watson AFR, Gee IL. The effects of smoking status and ventilation on environmental tobacco smoke concentrations in public areas of UK pubs and bars. Atmospheric Environment. 2003;37(23):3255-66.	Excluded by title
137	Carroll CE, Jones JE. Pressure-Appliance Therapy Following Premature Loss of Primary Molars. Journal of Dentistry for Children. 1982;49(5):347-51.	Excluded by title
138	Carvalho GS, Oliveira JR, Vasques ICF, Justi M, Santana MLT, Job MTP, et al. Steel mill waste effects on rice growth: comparison of chemical extractants on lead and zinc availability. Environ Sci Pollut Res Int. 2021;28(20):25844-57.	Excluded by title
139	Casián-Adem J, Cobos L, Waggoner WF, Fuks AB. Prefabricated Zirconia Crowns - A Solution to Treat Hypomineralized Permanent Molars: Report of a Case. J Clin Pediatr Dent. 2021;45(1):8-11.	Excluded by title
140	Castilho LS, Cotta FVMD, Bueno AC, Moreira AN, Ferreira EF, Magalhães CS. Validation of DIAGNOdent laser fluorescence and the International Caries Detection and Assessment System (ICDAS) in diagnosis of occlusal caries in permanent teeth: An in vivo study. European Journal of Oral Sciences. 2016;124(2):188-94.	Excluded by title
141	Castro LC, Galvão AC. Comparison of three different preparation methods in the improvement of sealant retention. Journal of Clinical Pediatric Dentistry. 2004;28(3):249-52.	Excluded by title
142	Castro VM, Katz JO, Hardman PK, Glaros AG, Spencer P. In vitro comparison of conventional film and direct digital imaging in the detection of approximal caries. Dentomaxillofacial Radiology. 2007;36(3):138-42.	Excluded by title
143	Cauwels RG, De Coster PJ, Mortier GR, Marks LA, Martens LC. Dentinogenesis imperfecta associated with short stature, hearing loss and mental retardation: a new syndrome with autosomal recessive inheritance? J Oral Pathol Med. 2005;34(7):444-6.	Excluded by title
144	Çelik D, Taşdemir T, Er K. Comparative study of 6 rotary nickel-titanium systems and hand instrumentation for root canal preparation in severely curved root canals of extracted teeth. Journal of Endodontics. 2013;39(2):278-82.	Excluded by title
145	Cernei ER, Maxim DC, Zetu IN. The influence of premature loss of temporary upper molars on permanent molars. Rev Med Chir Soc Med Nat Iasi. 2015;119(1):236-42.	Excluded by title
146	Chailertvanitkul P, Paphangkorakit J, Sooksantisakoonchai N, Pumas N, Pairojamornyoot W, Leela-apiradee N, et al. Randomized control trial comparing calcium hydroxide and mineral trioxide aggregate for partial pulpotomies in cariously exposed pulps of permanent molars. International Endodontic Journal. 2014;47(9):835-42.	Excluded by title
147	Chaipattanawan N, Chompu-Inwai P, Nirunsittirat A, Phinyo P, Manmontri C. Longevity of stainless steel crowns as interim restorations on young permanent first molars that have undergone vital pulp therapy treatment in children and factors associated with their treatment failure: A retrospective study of up to 8.5 years. Int J Paediatr Dent. 2022;32(6):925-37.	Excluded by title
148	Chang YI, Shin SJ, Baek SH. Three-dimensional finite element analysis in distal en masse movement of the maxillary dentition with the multiloop edgewise archwire. European Journal of Orthodontics. 2004;26(3):339-45.	Excluded by title
149	Chapple JR, Nunn JH. The oral health of children with clefts of the lip, palate, or both. Cleft Palate-Craniofacial Journal. 2001;38(5):525-8.	Excluded by title
150	Charbeneau GT, Dennison JB, Ryge G. A filled pit and fissure sealant: 18-month results. Journal of the American Dental Association (1939). 1977;95(2):299-306.	Excluded by title
151	Charbeneau GT, Dennison JB. Clinical success and potential failure after single application of a pit and fissure sealant: a four-year report. Journal of the American Dental Association (1939). 1979;98(4):559-64.	Excluded by title
152	Chatterjee R, Acevedo AM, Kleinberg I. Comparison of the Detection of Early Occlusal Caries in Extracted Human Permanent Molar Teeth by Electrical Conductance and Biopsy Means. J Clin Dent. 2019;30(2):1-5.	Excluded by title
153	Chau SS, King NM. An in vitro investigation of developmental defects of enamel under wet and dry conditions. New Zealand Dental Journal. 1989;85(381):78-82.	Excluded by title
154	Chaves JC, Santos TRd, Marsillac MdWSd, Alexandria A, Fidalgo TKdS. Assessment of Dental Caries and Intervention in the First Permanent Molars of Brazilian Children. Pesqui bras odontopediatria clín integr. 2021;21:e0010-e.	Excluded by title
155	Chawla N, Messer LB, Silva M. Clinical studies on molar-incisor-hypomineralisation part 2: development of a severity index. European archives of paediatric dentistry : official journal of the European Academy of Paediatric Dentistry. 2008;9(4):191-9.	Excluded by title
156	Chen H, Li W, Zhu Y. Improved window adaptive gray level co-occurrence matrix for extraction and analysis of texture characteristics of pulmonary nodules. Comput Methods Programs Biomed. 2021;208:106263.	Excluded by title
157	Chen T, Ruan DD, Zhang JH, Wang HL, Wu M, Wu QY, et al. Eucarbwenstols A-H, eight novel compounds from Eucalyptus robusta prevents MPC-5 injury via ROS modulation and regulation of mitochondrial membrane potential. Bioorg Chem. 2022;129:106159.	Excluded by title
158	Cheyne VD, Wessels KE. Impaction of permanent first molar with resorption and space loss in region of deciduous second molar. J Am Dent Assoc. 1947;35(11):774-87.	Excluded by title
159	Chintakanon K, Boonpinon P. Ectopic eruption of the first permanent molars: Prevalence and etiologic factors. Angle Orthodontist. 1998;68(2):153-60.	Excluded by title
160	Cho J, Kim DK. [Study on the shape and depth of the occlusal central fissure in permanent molar teeth]. Taehan Chikkwa Uisa Hyophoe Chi. 1989;27(10):959-64.	Excluded by title
161	Cho SY, Lee CK. Autotransplantation of a supplemental premolar: a case report. J Can Dent Assoc. 2007;73(5):425-9.	Excluded by title
162	Chong LY, Clarkson JE, Dobbyn-Ross L, Bhakta S. Slow-release fluoride devices for the control of dental decay. Cochrane Database Syst Rev. 2018;3(3):Cd005101.	Excluded by title
163	Chouchene F, Masmoudi F, Baaziz A, Maatouk F, Ghedira H. Clinical status and assessment of caries on first permanent molars in a group of 6- to 13-year-old Tunisian school children. Clin Exp Dent Res. 2023;9(1):240-8.	Excluded by title
164	Chowdhary N, Subba Reddy VV. Dentin comparison in primary and permanent molars under transmitted and polarised light microscopy: An in vitro study. Journal of Indian Society of Pedodontics and Preventive Dentistry. 2010;28(3):167-72.	Excluded by title
165	Clark W, Geneser M, Owais A, Kanellis M, Qian F. Success rates of Hall technique crowns in primary molars: a retrospective pilot study. Gen Dent. 2017;65(5):32-5.	Excluded by title
166	Corega C, Vaida L, Festila DG, Rigoni G, Albanese M, D'Agostino A, et al. Inclusion of all permanent mandibular molars and all maxillary second and third molars: A case report and review of the literature. Minerva Stomatologica. 2013;62:69-72.	Excluded by title
167	Corona SAM, Borsatto MC, Garcia L, Ramos RP, Palma-Dibb RG. Randomized, controlled trial comparing the retention of a flowable restorative system with a conventional resin sealant: One-year follow up. International Journal of Paediatric Dentistry. 2005;15(1):44-50.	Excluded by title
168	Correr GM, Alonso RCB, Consani S, Puppin-Rontani RM, Ferracane JL. In vitro wear of primary and permanent enamel. Simultaneous erosion and abrasion. American Journal of Dentistry. 2007;20(6):394-9.	Excluded by title
169	Cortes O, Garcia-Godoy F, Boj JR. Bond strength of resin-reinforced glass ionomer cements after enamel etching. American journal of dentistry. 1993;6(6):299-301.	Excluded by title
170	Costa EL, Bastos Filho PSdC, Moura MdS, Sousa TSd, Lemos A, Pedrosa MAC. Efeitos de um programa de exercícios em grupo sobre a força de preensão manual em idosas com baixa massa óssea. Arq bras endocrinol metab. 2012;56(5):313-8.	Excluded by title
171	Costa LdS, Alves SSS, Lima DDC, Dietrich L, Santos Filho PCF, Martins VdM. Lesão cervical não cariosa e hipersensibilidade dentinária: relato de caso clínico. ROBRAC. 2018;27(83):247-51.	Excluded by title
172	Costa WCdLB, Werneck MAF, Palmier AC. Secondary care in oral health in small municipalities: a cross-sectional evaluation of demand x access. RGO (Porto Alegre). 2018;66(1):70-6.	Excluded by title
173	Cote S, Geltman P, Nunn M, Lituri K, Henshaw M, Garcia RI. Dental caries of refugee children compared with US children. Pediatrics. 2004;114(6):e733-40.	Excluded by title
174	Coulomb E. [The 1st permanent molar in children. Clinical and therapeutic approach as a function of its anatomy, physiology and its particular pathology]. Inf Dent. 1986;68(39):3883-92.	Excluded by title
175	Counihan KP, O'Connell AC. Case report: pre-eruptive intra-coronal radiolucencies revisited. Eur Arch Paediatr Dent. 2012;13(4):221-6.	Excluded by title
176	Croll TP, Sundfeld RH. Resin-based composite reinforced sealant. ASDC J Dent Child. 1999;66(4):233-7, 28.	Excluded by title
177	Cunha SA, Soares CJ, Rosatto CMP, Vieira J, Pereira R, Soares PBF, et al. Effect of Endodontic Sealer in Young Molars Treated by Undergraduate Students - A Randomized Clinical Trial. Braz Dent J. 2020;31(6):589-97.	Excluded by title
178	Curzon MEJ, Ogden AR, Williams-Ward M, Cleaton-Jones PE. Case report: A medieval case of molar-incisor-hypomineralisation. British Dental Journal. 2015;219(12):583-7.	Excluded by title
179	da Silva Neto JM, dos Santos RL, Sampaio MCC, Sampaio FC, Passos IA. Radiographic diagnosis of incipient proximal caries: An Ex-Vivo study. Brazilian Dental Journal. 2008;19(2):97-102.	Excluded by title
180	Dabbagh B, Sigal MJ, Tompson BD, Titley K, Andrews P. Ectopic eruption of the permanent maxillary first molar: Predictive factors for irreversible outcome. Pediatric Dentistry. 2017;39(3):215-8.	Excluded by title
181	Dadpe MV, Chordiya NA, Kale YJ, Dahake PT, Kendre SB, Mankar SS. Evaluation of Efficiency of Distal Shoe Space Maintainer: A Systematic Review. Journal of Advanced Oral Research. 2023;14(1):11-20.	Excluded by title
182	Dall AQK, Sarwanand, Batool S, Arora S, Qureshi S, Bari MA. Location of pulp chamber in lower first permanent molars: In-vitro study. Journal of the Liaquat University of Medical and Health Sciences. 2020;19(2):122-7.	Excluded by title
183	Dammaschke T, Witt M, Ott K, Schäfer E. Scanning electron microscopic investigation of incidence, location, and size of accessory foramina in primary and permanent molars. Quintessence International. 2004;35(9):699-705.	Excluded by title
184	D'Angelo M, Margiotta V, Ammatuna P, Sammartano F. Treatment of prepubertal periodontitis: A case report and discussion. Journal of Clinical Periodontology. 1992;19(3):214-9.	Excluded by title
185	Dangy B. [The first permanent molar in orthodontic practice]. Cah Odontostomatol Touraine. 1976;8(3):13-6.	Excluded by title
186	Daozhang C, Yuxian C, Hua Z. Analysis of the serum biomarkers in human knee osteoarthritis. Osteoarthritis and Cartilage. 2012;20:S89.	Excluded by title
187	Darby IB, Lu J, Calache H. Radiographic study of the prevalence of periodontal bone loss in Australian school-aged children attending the Royal Dental Hospital of Melbourne. Journal of Clinical Periodontology. 2005;32(9):959-65.	Excluded by title
188	Dastouri M, Kowash M, Al-Halabi M, Salami A, Khamis AH, Hussein I. United Arab Emirates dentists' perceptions about the management of broken down first permanent molars and their enforced extraction in children: a questionnaire survey. Eur Arch Paediatr Dent. 2020;21(1):31-41.	Excluded by title
189	Dave M, Thomson F, Barry S, Horner K, Thakker N, Petersen HJ. The use of localised CBCT to image inflammatory collateral cysts: a retrospective case series demonstrating clinical and radiographic features. European Archives of Paediatric Dentistry. 2020;21(3):329-37.	Excluded by title
190	Davey KW. Effect of premature loss of primary molars on the anteroposterior position of maxillary first permanent molars and other maxillary teeth. Journal of dentistry for children. 1967;34(5):383-94.	Excluded by title
191	Davidovich E, Kreiner B, Peretz B. Treatment of severe pre-eruptive intracoronal resorption of a permanent second molar. Pediatric Dentistry. 2005;27(1):74-7.	Excluded by title
192	Davidovitch M, McInnis D, Lindauer SJ. The effects of lip bumper therapy in the mixed dentition. American Journal of Orthodontics and Dentofacial Orthopedics. 1997;111(1):52-8.	Excluded by title
193	Davies PH, Downer MC, Lennon MA. Periodontal bone loss in English secondary school children. A longitudinal radiological study. J Clin Periodontol. 1978;5(4):278-84.	Excluded by title
194	Dawson LR, Simon Jr JF, Taylor PP. Use of amalgam and stainless steel restorations for primary molars. ASDC journal of dentistry for children. 1981;48(6):420-2.	Excluded by title
195	de Carvalho RN, dos Santos Letieri A, Vieira TI, dos Santos TMP, Lopes RT, de Almeida Neves A, et al. Accuracy of visual and image-based ICDAS criteria compared with a micro-CT gold standard for caries detection on occlusal surfaces. Brazilian Oral Research. 2018;32.	Excluded by title
196	de Farias AL, Rojas-Gualdrón DF, Girotto Bussaneli D, Santos-Pinto L, Mejía JD, Restrepo M. Does molar-incisor hypomineralization (MIH) affect only permanent first molars and incisors? New observations on permanent second molars. Int J Paediatr Dent. 2022;32(1):1-10.	Excluded by title
197	de Mello Malta F, Amgarten D, Val FC, Cervato MC, de Azevedo BMC, de Souza Basqueira M, et al. Mass molecular testing for COVID19 using NGS-based technology and a highly scalable workflow. Sci Rep. 2021;11(1):7122.	Excluded by title
198	Deery C. Fissure seal or fluoride varnish? Evid Based Dent. 2016;17(3):77-8.	Excluded by title

199	De-la-Rosa-Gay C, Valmaseda-Castellón E, Gay-Escoda C. Predictive model of third molar eruption after second molar extraction. Am J Orthod Dentofacial Orthop. 2010;137(3):346-53.	Excluded by title
200	Demir T, Ates U, Cehreli B, Cehreli ZC. Autotransplantation of a supernumerary incisor as a replacement for fused tooth: 24-month follow-up. Oral Surgery, Oral Medicine, Oral Pathology, Oral Radiology and Endodontology. 2008;106(4):e1-e6.	Excluded by title
201	Dennison JB, Straffon LH, More FG. Evaluating tooth eruption on sealant efficacy. Journal of the American Dental Association (1939). 1990;121(5):610-4.	Excluded by title
202	Dhar V, Chen H. Evaluation of resin based and glass ionomer based sealants placed with or without tooth preparation - A two year clinical trial. Pediatric Dentistry. 2012;34(1):46-50.	Excluded by title
203	Dhillon J, Pathak A. Comparative evaluation of shear bond strength of three pit and fissure sealants using conventional etch or self-etching primer. Journal of Indian Society of Pedodontics and Preventive Dentistry. 2012;30(4):288-92.	Excluded by title
204	Dhindsa A, Pandit IK. Modified Willet's appliance for bilateral loss of multiple deciduous molars: a case report. J Indian Soc Pedod Prev Dent. 2008;26(3):132-5.	Excluded by title
205	Dhull KS, Bhojraj N, Yadav S, Prabhakaran SD. Modified distal shoe appliance for the loss of a primary second molar: a case report. Quintessence Int. 2011;42(10):829-33.	Excluded by title
206	Di Massa E. [Space closure in a 4 first permanent molar extraction case]. Mondo Ortod. 1988;13(4):113-8.	Excluded by title
207	Dias C, Closs LQ, Fontanella V, de Araujo FB. Vertical alveolar growth in subjects with infraoccluded mandibular deciduous molars. American Journal of Orthodontics and Dentofacial Orthopedics. 2012;141(1):81-6.	Excluded by title
208	Diaz MCA, Pinzan A, Freitas MRd. Extração de primeiros molares permanentes: apresentação de um caso. Ortodontia. 1992;25(1):47-53.	Excluded by title
209	Dickson JA, Jones AG. Extraction of four second permanent molars in the presence of severe premolar crowding: a case report. Dental update. 1996;23(8):339-40, 42-43.	Excluded by title
210	Dimri M, Jain A. Stainless steel crown bridge replacing permanent molar in the adolescent patient: a case report. J Indian Soc Pedod Prev Dent. 2001;19(2):74-6.	Excluded by title
211	Dineshshankar J, Sivakumar M, Balasubramanium A, Kesavan G, Karthikeyan M, Prasad VS. Taurodontism. Journal of Pharmacy and Bioallied Sciences. 2014;6:S13-S5.	Excluded by title
212	Dixit LP, Gurung CK, Gurung N, Joshi N. Reasons underlying the extraction of permanent teeth in patients attending Peoples Dental College and Hospital. Nepal Med Coll J. 2010;12(4):203-6.	Excluded by title
213	Do T, Church B, Veríssimo C, Hackmyer SP, Tantbirojn D, Simon JF, et al. Cuspal flexure, depth-of-cure, and bond integrity of bulk-fill composites. Pediatric Dentistry. 2014;36(7):468-73.	Excluded by title
214	Domínguez A, Aznar T. Removable prostheses for preschool children: Report of two cases. Quintessence International. 2004;35(5):397-400.	Excluded by title
215	Dougherty HL. The effect of mechanical forces upon the mandibular buccal segments during orthodontic treatment. American Journal of Orthodontics. 1968;54(2):83-103.	Excluded by title
216	Drummond BK, Bimstein E. Prevalence of marginal alveolar bone loss in children referred for treatment to the Paediatric Clinic at the School of Dentistry, University of Otago. N Z Dent J. 1995;91(406):138-40.	Excluded by title
217	Ducommun F, Bornstein MM, Bosshardt D, Katsaros C, Dula K. Diagnosis of tooth ankylosis using panoramic views, cone beam computed tomography, and histological data: A retrospective observational case series study. European Journal of Orthodontics. 2018;40(3):231-8.	Excluded by title
218	Dugoni SA, Lee JS. Mixed dentition case report. American Journal of Orthodontics and Dentofacial Orthopedics. 1995;107(3):239-44.	Excluded by title
219	Dugues P, Martin M, Abe E, Etting I, Edel Y, Alvarez JC, et al. Targeted and untargeted screening of new psychoactive substances (NPS) and classical drugs of abuse in Paris using hair testing: A 10-years study (2012–2021). Toxicologie Analytique et Clinique. 2022;34(3):S80.	Excluded by title
220	Dukic W, Glavina D. Clinical evaluation of three fissure sealants: 24 month follow-up. European archives of paediatric dentistry : official journal of the European Academy of Paediatric Dentistry. 2007;8(3):163-6.	Excluded by title
221	Duncalf WV, Wilson NH. A comparison of the marginal and internal adaptation of amalgam and resin composite restorations in small to moderate-sized Class II preparations of conventional design. Quintessence Int. 2000;31(5):347-52.	Excluded by title
222	Duncan WK, Ashrafi MH. Ectopic eruption of the mandibular first permanent molar. J Am Dent Assoc. 1981;102(5):651-4.	Excluded by title
223	Dury DC, Roberts MW, Miser JS, Folio J. Dental root agenesis secondary to irradiation therapy in a case of rhabdomyosarcoma of the middle ear. Oral Surgery, Oral Medicine, Oral Pathology. 1984;57(6):595-9.	Excluded by title
224	Eghbal MJ, Asgary S, Baglue RA, Parirokh M, Ghoddusi J. MTA pulpotomy of human permanent molars with irreversible pulpitis. Australian Endodontic Journal. 2009;35(1):4-8.	Excluded by title
225	Einwag J. Effect of entirely preformed stainless steel crowns on periodontal health in primary, mixed dentitions. ASDC journal of dentistry for children. 1984;51(5):356-9.	Excluded by title
226	Ekambaram M, Yiu CKY, Matinlinna JP. Effect of solvents on dentin collagen cross-linking potential of carbodiimide. Journal of Adhesive Dentistry. 2015;17(3):219-26.	Excluded by title
227	Eklund SA, Ismail AI. Time of Development of Occlusal and Proximal Lesions: Implications for Fissure Sealants. Journal of Public Health Dentistry. 1986;46(2):114-21.	Excluded by title
228	Ekstrand KR, Cordeschi T, Abreu-Placeres N. ICCMS™ root caries lesions stages and their underlying depth towards the pulp: an in vitro study with histologic evaluation. Clin Oral Investig. 2022;26(3):2597-605.	Excluded by title
229	Ekstrand KR, Nielsen LA, Carvalho JC, Thylstrup A. Dental plaque and caries on permanent first molar occlusal surfaces in relation to sagittal occlusion. Scand J Dent Res. 1993;101(1):9-15.	Excluded by title
230	El Ashiry EA, Farsi NM. Prevalence of extra root canal orifices of maxillary first permanent molars in a Saudi subpopulation utilizing microcomputed tomography. Journal of Contemporary Dental Practice. 2018;19(11):1312-6.	Excluded by title
231	El-Fateh T, Ruf S. Herbst treatment with mandibular cast splints - Revisited. Angle Orthodontist. 2011;81(5):820-7.	Excluded by title
232	Elgalaid TO, Creanor SL, Creanor S, Hall AF. The permeability of natural dentine caries before and after restoration: An in vitro study. Journal of Dentistry. 2007;35(8):656-63.	Excluded by title
233	El-Mowafy O, El-Badrawy W, Wasef M, Omar H, Kermanshahi S. Efficacy of new LED light-curing units in hardening of Class II composite restorations. J Can Dent Assoc. 2007;73(3):253.	Excluded by title
234	Emërllahu F, Beqiri M, Iliazi-Shahiqi D. Evaluation of treatment needs of permanent first molar teeth among patients in the main center of family medicine in Gjilan Kosovo. Acta Stomatologica Croatica. 2018;52(2):164.	Excluded by title
235	Epasinghe DJ, Yiu CKY. Effect of etching on bonding of a self-etch adhesive to dentine affected by amelogenesis imperfecta. Journal of investigative and clinical dentistry. 2018;9(1).	Excluded by title
236	Erickson PR. Estimation of the caries-related risk associated with infant formulas. Pediatric Dentistry. 1998;20(7):395-403.	Excluded by title
237	Eriksen HM, Grytten J, Hoist D. Is there a long-term caries-preventive effect of sugar restrictions during world war II? Acta Odontologica Scandinavica. 1991;49(3):163-8.	Excluded by title
238	Espinosa R, Valencia R, Uribe M, Ceja I, Saadia M. Enamel deproteinization and its effect on acid etching: An in vitro study. Journal of Clinical Pediatric Dentistry. 2008;33(1):13-9.	Excluded by title
239	Evancusky JW, Meiers JC. Microleakage of Compoglass-F and Dyract-AP compomers in Class V preparations after salivary contamination. Pediatr Dent. 2000;22(1):39-42.	Excluded by title
240	Fagrell TG, Dietz W, Jälevik B, Norén JG. Chemical, mechanical and morphological properties of hypomineralized enamel of permanent first molars. Acta Odontologica Scandinavica. 2010;68(4):215-22.	Excluded by title
241	Fagrell TG, Lingström P, Olsson S, Steiniger F, Norén JG. Bacterial invasion of dentinal tubules beneath apparently intact but hypomineralized enamel in molar teeth with molar incisor hypomineralization. International Journal of Paediatric Dentistry. 2008;18(5):333-40.	Excluded by title
242	Fagrell TG, Salmon P, Melin L, Norén JG. Onset of Molar Incisor Hypomineralization (MIH). Swedish Dental Journal. 2013;37(2):61-70.	Excluded by title
243	Falk WV. Retention of deciduous molar crown after early loss of mandibular permanent molar. Oral Surg Oral Med Oral Pathol. 1982;54(4):479.	Excluded by title
244	Farah R, Drummond B, Swain M, Williams S. Linking the clinical presentation of molar-incisor hypomineralisation to its mineral density. International Journal of Paediatric Dentistry. 2010;20(5):353-60.	Excluded by title
245	Farcașiu AT, Luca R, Didilescu A, Stanciu IA, Farcasiu C, Vinereanu A, et al. Congenitally missing second permanent molars in non-syndromic patients (Review). Experimental and Therapeutic Medicine. 2022;23(2).	Excluded by title
246	Fearne J, Anderson P, Davis GR. 3D X-ray microscopic study of the extent of variations in enamel density in first permanent molars with idiopathic enamel hypomineralisation. British Dental Journal. 2004;196(10):634-8.	Excluded by title
247	Fedorowicz Z, Nasser M, Wilson N. Adhesively bonded versus non-bonded amalgam restorations for dental caries. Cochrane Database Syst Rev. 2009(4):Cd007517.	Excluded by title
248	Feigal RJ, Quelhas I. Clinical trial of a self-etching adhesive for sealant application: Success at 24 months with Prompt L-Pop. American Journal of Dentistry. 2003;16(4):249-51.	Excluded by title
249	Felicita AS, Khader SA. Comparison of two treatment protocols for intrusion and retraction of maxillary anterior teeth using mini-implants : A prospective clinical trial. J Orofac Orthop. 2024;85(1):13-29.	Excluded by title
250	Felicita AS, Wahab TU. Intrusion of the maxillary posterior teeth with a single buccal mini-implant positioned bilaterally in young adults with a tendency towards hyperdivergence: A clinical study. J Orthod. 2022;49(3):338-46.	Excluded by title
251	Fenner DE, Zutshi M, Lucente V, Nihira MA, Culligan P, Mellgren A. Decrease in health resource utilization following TOPAS® treatment for fecal incontinence. Female Pelvic Medicine and Reconstructive Surgery. 2015;21(5):S13-S4.	Excluded by title
252	Ferdianakis K, White GE. Newer class I cavity preparation for permanent teeth using air abrasion and composite restoration. Journal of Clinical Pediatric Dentistry. 1999;23(3):201-16.	Excluded by title
253	Ferdianakis K. Microleakage reduction from newer esthetic restorative materials in permanent molars. Journal of Clinical Pediatric Dentistry. 1998;22(3):221-9.	Excluded by title
254	Ferlin LH, Daruge AD, Daruge RJ, Rancan SV. [Prevalence of first permanent molar loss in 6 to 12-year-old students of both sexes in Ribeirão Preto]. Rev Odontol Univ Sao Paulo. 1989;3(1):239-45.	Excluded by title
255	Fernández-Barrera M, Lara-Carrillo E, Scougall-Vilchis RJ, Pontigo-Loyola AP, Ávila-Burgos L, Casanova-Rosado JF, et al. Study protocol of the cost-effectiveness comparison of two preventive methods in the incidence of caries: A randomized, controlled clinical trial. Medicine (Baltimore). 2019;98(30):e16634.	Excluded by title
256	Ferreira JTL, Romano FL, Sasso Stuani MB, Assed Carneiro FC, Nakane Matsumoto MA. Traction of impacted canines in a skeletal Class III malocclusion: A challenging orthodontic treatment. American Journal of Orthodontics and Dentofacial Orthopedics. 2017;151(6):1159-68.	Excluded by title
257	Fichera G, Greco M, Leonardi R. Effectiveness of the Passive Lingual Arch for E Space Maintenance in Subjects with Anterior or Posterior Rotation of the Mandible: A Retrospective Study. Medical Principles and Practice. 2010;20(2):165-70.	Excluded by title
258	Fierro Monti C, Vera Morales A, Pérez Flores MA. Erupción ectópica del primer molar permanente Superior, evolución y tratamiento a 3 años: Caso clínico. Int j odontostomatol (Print). 2007;1(2):185-90.	Excluded by title
259	Figueira HS, Medina PO, de Jesus GP, Hanan ARA, Júnior ECS, Hanan SA. Oral findings in Coffin-Siris syndrome: A case report. Revista Portuguesa de Estomatologia, Medicina Dentaria e Cirurgia Maxilofacial. 2021;62(1):42-9.	Excluded by title
260	Flores Cañas DE, Flores Velásquez EM, García Reyes NA, Reyes Bermúdez RO, Rivera Ventura JO. Prevalencia de maloclusiones en niños de 9-15 años que asisten a determinadas escuelas de los departamentos de San Salvador, San Miguel y La libertad. 2003. p. 86-.	Excluded by title
261	Folayan MO, Ozeigbe EO, Onyejaeka N, Chukwumah NM, Oyedele T. Non-third molar related pericoronitis in a sub-urban Nigeria population of children. Niger J Clin Pract. 2014;17(1):18-22.	Excluded by title
262	Foley J. A prospective study of the use of nitrous oxide inhalation sedation for dental treatment in anxious children. Eur J Paediatr Dent. 2005;6(3):121-8.	Excluded by title
263	Foley J. Efficacy of nitrous oxide inhalation sedation and first permanent molar tooth extractions. SAAD Dig. 2007;23:3-9.	Excluded by title
264	Foley JI. Dental students consistency in applying the ICDAS system within paediatric dentistry. Eur Arch Paediatr Dent. 2012;13(6):319-22.	Excluded by title
265	Fortini A, Lupoli M, Giuntoli F, Franchi L. Dentoskeletal effects induced by rapid molar distalization with the first class appliance. American Journal of Orthodontics and Dentofacial Orthopedics. 2004;125(6):697-704.	Excluded by title
266	Fowler KJ, Santymire RM. Characterizing zoo-housed Bactrian camel (Camelus bactrianus) reproduction using gonadal steroid metabolite analysis in feces. Domest Anim Endocrinol. 2022;80:106721.	Excluded by title
267	Franchi L, Vangelisti A, Fortini A, Baccetti T. Dentoskeletal effects of an intraoral appliance for molar distalization: A systematic comparison with alternative appliances. Mondo Ortodontico. 2011;36(2):53-63.	Excluded by title

268	Franchi M, Breschi L, Ruggeri O. Cusp fracture resistance in composite-amalgam combined restorations. J Dent. 1999;27(1):47-52.	Excluded by title
269	Francis R, Mascarenhas AK, Soparkar P, Al-Mutawaa S. Retention and effectiveness of fissure sealants in Kuwaiti school children. Community Dental Health. 2008;25(4):211-5.	Excluded by title
270	Francisco DdS, Faria FR, Peruzzolo CC, Yamaguti WP, Paulin E. Relationship between handgrip strength and pulmonary capacity in patients on hemodialysis. Fisioter Mov (Online). 2020;33:e003348-e.	Excluded by title
271	Frank CA. Treatment options for impacted teeth. Journal of the American Dental Association. 2000;131(5):623-+.	Excluded by title
272	Friedl K, Hiller KA, Friedl KH. Clinical performance of a new glass ionomer based restoration system: A retrospective cohort study. Dental Materials. 2011;27(10):1031-7.	Excluded by title
273	Frigidi GA, Gotta SL. Cementoblastoma asociado a primer molar permanente. Reporte de un caso. Rev Asoc Odontol Argent. 2023;111(1):5-.	Excluded by title
274	Fu PS, Wang JC, Chen CH, Huang TK, Tseng CH, Hung CC. Management of unilaterally deep impacted first, second, and third mandibular molars. Angle Orthodontist. 2012;82(3):565-71.	Excluded by title
275	Fuge KN, Stuck AM, Love RM. A comparison of digitally scanned radiographs with conventional film for the detection of small endodontic instruments. Int Endod J. 1998;31(2):123-6.	Excluded by title
276	Fumes AC, Longo DL, De Rossi A, Da Silva Fidalgo TK, De Paula E Silva FWG, Borsatto MC, et al. Microleakage of sealants after phosphoric acid, Er: YAG laser and air abrasion enamel conditioning: Systematic review and meta-analysis. Journal of Clinical Pediatric Dentistry. 2017;41(3):167-72.	Excluded by title
277	Fung L, Smales R, Ngo H, Mount G. Diagnostic comparison of three groups of examiners using visual and laser fluorescence methods to detect occlusal caries in vitro. Australian Dental Journal. 2004;49(2):67-71.	Excluded by title
278	Fux-Noy A. Distal Migration and Ectopic Eruption of the Mandibular First Premolar: A Case Report. J Clin Pediatr Dent. 2019;43(5):364-6.	Excluded by title
279	Fux-Noy A. Distal Migration and Ectopic Eruption of the Mandibular First Premolar: Case Report. J Clin Pediatr Dent. 2020;44(2):127-9.	Excluded by title
280	Fyffe HE, Deery C, Nugent ZJ, Nuttall NM, Pitts NB. In vitro validity of the Dundee Selectable Threshold Method for caries diagnosis (DSTM). Community Dent Oral Epidemiol. 2000;28(1):52-8.	Excluded by title
281	Gaardmand E, Poulsen S, Haubek D. Pilot study of minimally invasive cast adhesive copings for early restoration of hypomineralised first permanent molars with post-eruptive breakdown. European Archives of Paediatric Dentistry. 2013;14(1):35-9.	Excluded by title
282	Gandini M, Vertuan V, Davis JM. A Comparative-Study between Visible-Light-Activated and Autopolymerizing Sealants in Relation to Retention. Journal of Dentistry for Children. 1991;58(4):297-9.	Excluded by title
283	Gao W, Peng D, Smales RJ, Yip KHK. Comparison of atraumatic restorative treatment and conventional restorative procedures in a hospital clinic: Evaluation after 30 months. Quintessence International. 2003;34(1):31-7.	Excluded by title
284	Garcia-Godoy F, de Araujo FB. Enhancement of fissure sealant penetration and adaptation: the enameloplasty technique. The Journal of clinical pediatric dentistry. 1994;19(1):13-8.	Excluded by title
285	García-Godoy F, Dodge WW, Donohue M, O'Quinn JA. Composite resin bond strength after enamel bleaching. Operative dentistry. 1993;18(4):144-7.	Excluded by title
286	Garcia-Godoy F, Dodge WW, Donohue M, O'Quinn JA. Effect of a fluoridated etchant on the shear bond strength of a composite resin to enamel. International Journal of Paediatric Dentistry. 1992;2(1):25-30.	Excluded by title
287	Garcia-Godoy F, O'Quinn JA, Donohue M. Effect of a triclosan/copolymer/fluoride dentifrice prophylaxis on the shear bond strength of a composite resin to enamel. American journal of dentistry. 1991;4(4):167-9.	Excluded by title
288	García-Godoy F, Rodríguez M, Barbería E. Dentin bond strength of fluoride-releasing materials. American Journal of Dentistry. 1996;9(2):80-2.	Excluded by title
289	Garcia-Godoy F. Dentin surface treatment and shear bond strength of a light-cured glass ionomer. American journal of dentistry. 1992;5(5):283-5.	Excluded by title
290	Garcia-Godoy F. Shear bond strength of a resin composite to enamel treated with an APF gel. Pediatric dentistry. 1993;15(4):272-4.	Excluded by title
291	Garcia-Godoy F. Triclosan/copolymer/NAF dentifrice prophylaxis, reduced etching time and shear bond strength of a resin composite to enamel. American journal of dentistry. 1992;5(6):312-4.	Excluded by title
292	Garot E, Couture-Veschambre C, Manton D, Beauval C, Rouas P. Analytical evidence of enamel hypomineralisation on permanent and primary molars amongst past populations. Scientific Reports. 2017;7.	Excluded by title
293	Garot E, Denis A, Delbos Y, Manton D, Silva M, Rouas P. Are hypomineralised lesions on second primary molars (HSPM) a predictive sign of molar incisor hypomineralisation (MIH)? A systematic review and a meta-analysis. Journal of Dentistry. 2018;72:8-13.	Excluded by title
294	Geduk N, Ozdemir M, Erbas Unverdi G, Ballikaya E, Cehreli ZC. Clinical and radiographic performance of preformed zirconia crowns and stainless-steel crowns in permanent first molars: 18-month results of a prospective, randomized trial. BMC Oral Health. 2023;23(1 C7 - 828).	Excluded by title
295	Gee IL, Watson AFR, Carrington J. The contribution of environmental tobacco smoke to indoor pollution in pubs and bars. Indoor and Built Environment. 2005;14(3-4):301-6.	Excluded by title
296	Gemert-Schriks MCM, Amerongen WE, Cate JM, Aartman IHA. Three-year survival of single- and two-surface ART restorations in a high-caries child population. Clinical Oral Investigations. 2007;11(4):337-43.	Excluded by title
297	Generali L, Righi E, Todesca MV, Consolo U. Canal shaping with WaveOne reciprocating files: Influence of operator experience on instrument breakage and canal preparation time. Odontology. 2014;102(2):217-22.	Excluded by title
298	Gergely L. The frequency of deep bite in juvenile persons. Fogorvosi Szemle. 1949;42((12)):422-6.	Excluded by title
299	Geron S, Shpack N, Kandos S, Davidovitch M, Vardimon AD. Anchorage loss--a multifactorial response. Angle Orthod. 2003;73(6):730-7.	Excluded by title
300	Geuther M, Kleber BM, Strahlendorf F. [The relevance of the loss of first permanent molar for the periodontium of neighbouring teeth]. Dtsch Stomatol (1990). 1991;41(7):261-2.	Excluded by title
301	Ghanim A, Morgan M, Mariño R, Bailey D, Manton D. Molar-incisor hypomineralisation: Prevalence and defect characteristics in Iraqi children. International Journal of Paediatric Dentistry. 2011;21(6):413-21.	Excluded by title
302	Ghoul-Mazgar S. RANK, RANKL and OPG expressions in a permanent molar with a replacement resorption. Odontology. 2013;101(2):239-43.	Excluded by title
303	Gillcrist JA, Vaughan MP, Plumlee GN, Jr., Wade G. Clinical sealant retention following two different tooth-cleaning techniques. J Public Health Dent. 1998;58(3):254-6.	Excluded by title
304	Girotti AW. Photosensitized cross-linking of erythrocyte membrane proteins. Evidence against participation of amino groups in the reaction. Biochim Biophys Acta. 1980;602(1):45-56.	Excluded by title
305	Goldberg D, Turley PK. Orthodontic space closure of the edentulous maxillary first molar area in adults. Int J Adult Orthodon Orthognath Surg. 1989;4(4):255-66.	Excluded by title
306	Göllner N, Winkler J, Göllner P, Gkantidis N. Effect of mandibular first molar mesialization on alveolar bone height: a split mouth study. Progress in Orthodontics. 2019;20(1).	Excluded by title
307	Gomez F, Fuentes J, Saravia D, Silva M. Induction of root development and apical closure in permanent mandibular molar with irreversible pulpitis through total pulpotomy with application of mineral trioxide aggregate. Int j odontostomatol (Print). 2020;14(2):144-9.	Excluded by title
308	Gonçalves RA, Vargas IA, Ruschel HC. Abordagem clínica da erupção ectópica de um primeiro molar permanente superior - Relato de caso. Stomatos. 2012;18(35):16-25.	Excluded by title
309	Gonzalez-Ocasio J, Stevens M. Autotransplantation of Third Molars With Platelet-Rich Plasma for Immediate Replacement of Extracted Non-Restorable Teeth: A Case Series. Journal of Oral and Maxillofacial Surgery. 2017;75(9):1833.e1-.e6.	Excluded by title
310	Goto G, Zhang Y. Study of cervical pulp horns in human primary molars. J Clin Pediatr Dent. 1995;20(1):41-4.	Excluded by title
311	Gourraud JB, Chaix MA, Shohoudi A, Pagé P, Dubuc M, Thibault B, et al. Transvenous Lead Extraction in Adults With Congenital Heart Disease: Insights From a 20-Year Single-Center Experience. Circ Arrhythm Electrophysiol. 2018;11(2):e005409.	Excluded by title
312	Gozzi I, Rizzo F, Bagattoni S, Biondi E. Regional odontodysplasia: case report and literature review. Dental Cadmos. 2023;91(3):178-85.	Excluded by title
313	Graf H. The First Molar from Jaw Orthopedic and Gnathologic Sight. Stomatologie der DDR. 1987;37(3):155-9.	Excluded by title
314	Grewal N, Sharma N, Kaur N. Surface remineralization potential of nano-hydroxyapatite, sodium monofluorophosphate, and amine fluoride containing dentifrices on primary and permanent enamel surfaces: An in vitro study. Journal of Indian Society of Pedodontics and Preventive Dentistry. 2018;36(2):158-66.	Excluded by title
315	Grine FE, Holt S, Brink JS, du Plessis A. Enamel pearls: Their occurrence in recent human populations and earliest manifestation in the modern human lineage. Archives of Oral Biology. 2019;101:147-55.	Excluded by title
316	Grine FE. Enamel thickness of deciduous and permanent molars in modern Homo sapiens. American Journal of Physical Anthropology. 2005;126(1):14-31.	Excluded by title
317	Groper JN. Ectopic eruption of a mandibular first permanent molar: report of an unusual case. ASDC J Dent Child. 1992;59(3):228-30.	Excluded by title
318	Grošelj M, Jan J. Molar incisor hypomineralisation and dental caries among children in Slovenia. Eur J Paediatr Dent. 2013;14(3):241-5.	Excluded by title
319	Gu Q, Feng Z, Liang Q, Li M, Wang W, Rong P. [Development of a radiomics signature to predict Ki-67 expression level in non-small cell lung cancer]. Zhong Nan Da Xue Xue Bao Yi Xue Ban. 2018;43(11):1216-22.	Excluded by title
320	Guelmann M, Bonnin S, Primosch RE, Söderholm KJ. Microleakage and wall adaptation of conservative restorations. Am J Dent. 2002;15(6):407-11.	Excluded by title
321	Gugnani N, Gugnani S. Sealants generally show equal performance regardless of tooth type and position. Evid Based Dent. 2018;19(2):40-1.	Excluded by title
322	Gujjar KR, Indushekar KR, Amith HV, Sharma SL. Modified distal shoe appliance--fabrication and clinical performance. J Dent Child (Chic). 2012;79(3):185-8.	Excluded by title
323	Gunda SA, Patil A, Varekar A. First permanent molar root development arrest associated with compound odontoma. BMJ Case Rep. 2013;2013.	Excluded by title
324	Güngör HC, Canoğlu E, Çehreli ZC. The effects of dentin adhesives and liner materials on the microleakage of class II resin composite restorations in primary and permanent teeth. Journal of Clinical Pediatric Dentistry. 2014;38(3):223-8.	Excluded by title
325	Halicioglu K, Toptas O, Akkas I, Celikoglu M. Permanent first molar extraction in adolescents and young adults and its effect on the development of third molar. Clin Oral Investig. 2014;18(5):1489-94.	Excluded by title
326	Hall FM, Cook PA. Resorption of a first permanent molar. Br Dent J. 1989;166(1):19-20.	Excluded by title
327	Haznedaroğlu F, Ersev H, Odabaşi H, Yetkin G, Batur B, Aşçi S, et al. Incidence of patent furcal accessory canals in permanent molars of a Turkish population. International Endodontic Journal. 2003;36(8):515-9.	Excluded by title
328	Heliotis I, Gakhal M, Whatling R. Resorption of maxillary first permanent molars by impacted maxillary second premolars: A case series. Dental Update. 2020;47(11):946-9.	Excluded by title
329	Hennessy J, Al-Awadhi EA, Dwyer LO, Leith R. Treatment of ectopic first permanent molar teeth. Dent Update. 2012;39(9):656-8, 60-1.	Excluded by title
330	Herman RJ, Currier GF, Miyake A. Mini-implant anchorage for maxillary canine retraction: A pilot study. American Journal of Orthodontics and Dentofacial Orthopedics. 2006;130(2):228-35.	Excluded by title
331	Hernandez M, Rivero AR, Mendoza DM. Isotopic and paleopathological analysis of Pre-Columbian secondary interments at Cueva Vigía, Sancti Spiritus, Cuba. American Journal of Physical Anthropology. 2017;162:214.	Excluded by title
332	Hernández Ortega E, Taboada Aranza O. Prevalencia y algunos factores de riesgo de caries dental en el primer molar permanente en una población escolar de 6 a 12 años de edad. Rev ADM. 2017;74(3):141-5.	Excluded by title
333	Hernandéz-Gatón P, Serrano CR, Nelson Filho P, De Castañeda ER, Lucisano MP, Da Silva RAB, et al. Stepwise Excavation Allows Apexogenesis in Permanent Molars with Deep Carious Lesions and Incomplete Root Formation. Caries Research. 2015;49(6):637-9.	Excluded by title
334	Hildebolt CF, Zerbolio DJ, Jr., Shrout MK, Ritzi S, Gravier MJ. Radiometric Classification of Alveolar Bone Health. Journal of Dental Research. 1992;71(9):1594-7.	Excluded by title
335	Hilgers KK, Kinane DE, Scheetz JP. Association between childhood obesity and smooth-surface caries in posterior teeth: a preliminary study. Pediatr Dent. 2006;28(1):23-8.	Excluded by title
336	Holloway PJ, Blinkhorn AS, Hassall DC, Mellor AC, Worthington HV. An assessment of capitation in the General Dental Service Contract 1. The level of caries and its treatment in regularly attending children and adolescents. British Dental Journal. 1997;182(11):418-23.	Excluded by title
337	Holroyd I, Rule DC. Adenomatoid odontogenic tumour in a 12-year-old boy. Int J Paediatr Dent. 1997;7(2):101-6.	Excluded by title
338	Holtan JR, Nystrom GP, Olin PS, Phelps RA, 2nd, Phillips JJ, Douglas WH. Bond strength of six dentinal adhesives. J Dent. 1994;22(2):92-6.	Excluded by title
339	Honório HM, Rios D, Santos CF, Buzalaf MAR, Machado MAAM. Influence of dental plaque on human enamel erosion: In situ/ex vivo study. Oral Health and Preventive Dentistry. 2010;8(2):179-84.	Excluded by title
340	Hou GL, Tsai CC. The morphology of root fusion in Chinese adults (I). Grades, types, location and distribution. Journal of Clinical Periodontology. 1994;21(4):260-4.	Excluded by title

341	Hou J, Qin Y, Hu Y, Li F, Wen L, Lu Q, et al. Texture analysis based on intravoxel incoherent motion DWI for stratification of the clinical stages of nasopharyngeal cancer. International Journal of Clinical and Experimental Medicine. 2019;12(2):1897-905.	Excluded by title
342	Houpt M, Fukus A, Eidelman E. The preventive resin (composite resin/sealant) restoration: nine-year results. Quintessence Int. 1994;25(3):155-9.	Excluded by title
343	Howard RD. Skeletal changes with extra oral traction. European Journal of Orthodontics. 1982;4(3):197-202.	Excluded by title
344	Hsiao CC, Boynton JR. Etiology, Classification and Management of Ectopic Eruption of Permanent First Molars. J Mich Dent Assoc. 2016;98(1):26-30.	Excluded by title
345	Hubbard GW, Nanda RS, Currier GF. A cephalometric evaluation of nonextraction cervical headgear treatment in Class II malocclusions. Angle Orthodontist. 1994;64(5):359-70.	Excluded by title
346	Hugo B, Otto A, Stassinakis A, Hofmann N, Klaiber B. [A retrospective in vivo study of Sonicsys approx restorations]. Schweiz Monatsschr Zahnmed. 2001;111(2):152-8.	Excluded by title
347	Hunter B, Hunter ML, Pizarro KA. Complete eruption of a malformed mandibular permanent molar following extraction of a complex odontome. Int J Paediatr Dent. 1991;1(1):31-4.	Excluded by title
348	Hysi D, Kuscu OO, Droboniku E, Toti C, Xhemnica L, Caglar E. Prevalence and aetiology of Molar-Incisor Hypomineralisation among children aged 8-10 years in Tirana, Albania. European Journal of Paediatric Dentistry. 2016;17(1):75-9.	Excluded by title
349	Ibrahim SH, Eisa MSMS. Microshear Bond Strength, Ultramorphological, and Elemental Assessment of Gold–Silver Nanoparticle-treated Dentin Bonded to Resin Composite with Different Adhesive Modes. Journal of Contemporary Dental Practice. 2022;23(7):679-87.	Excluded by title
350	Ikeshima A. Papillon-Lefévre syndrome: a highly-suspected case. Journal of oral science. 2006;48(4):257-60.	Excluded by title
351	Imparato JCP, Moreira KMS, Olegário IC, da Silva S, Raggio DP. Partial caries removal increases the survival of permanent tooth: a 14-year case report. Eur Arch Paediatr Dent. 2017;18(6):423-6.	Excluded by title
352	Innes N, Borrie F, Bearn D, Evans D, Rauchhaus P, McSwiggan S, et al. Should I eXtract Every Six dental trial (SIXES): study protocol for a randomized controlled trial. Trials. 2013;14:59.	Excluded by title
353	Iqbal Z, Qureshi AH. MTA monoblock obturation technique in endodontic retreatment. J Coll Physicians Surg Pak. 2014;24 Suppl 3:S180-2.	Excluded by title
354	Ish T, Suckling G. The Severity of Dental Fluorosis in Children Exposed to Water with a High Fluoride Content for Various Periods of Time. Journal of Dental Research. 1991;70(6):952-6.	Excluded by title
355	Ishikawa I, Umeda M, Laosrisin N. Clinical, bacteriological, and immunological examinations and the treatment process of two Papillon-Lefevre syndrome patients. Journal of Periodontology. 1994;65(4):364-71.	Excluded by title
356	Ismail MQ, Lauridsen E, Andreasen JO, Hermann NV. Ectopic eruption of the second premolar: an analysis of four different treatment approaches. Eur Arch Paediatr Dent. 2020;21(1):119-27.	Excluded by title
357	Jaafar N, Razak IA, Nor GM. Trends in tooth loss due to caries and periodontal disease by tooth type. Singapore Dent J. 1989;14(1):39-41.	Excluded by title
358	Jagailloux Y, Duval X, Le Saos F, Rouille A, Constantin V, Gaulier JM, et al. QTc prolongation after consumption of NPS: about a case. Toxicologie Analytique et Clinique. 2021;33(2):153-9.	Excluded by title
359	Jagailloux Y, Duval X, Le Saos F, Rouille A, Constantin V, Gaulier JM, et al. Toxicologie Analytique et Clinique. 2021;33(3):S56.	Excluded by title
360	Jainaen A, Mahakunakorn N, Arayatrakullikit U, Sutthiprapaporn P, Noisombat R. Cone-beam computed tomography evaluation of curved root canals prepared using reciprocal rotary files and rotational rotary files. Journal of Conservative Dentistry. 2018;21(1):32-6.	Excluded by title
361	Jälevik B, Dietz W, Norén JG. Scanning electron micrograph analysis of hypomineralized enamel in permanent first molars. International Journal of Paediatric Dentistry. 2005;15(4):233-40.	Excluded by title
362	Jälevik B, Klingberg G. Treatment outcomes and dental anxiety in 18-year-olds with MIH, comparisons with healthy controls - A longitudinal study. International Journal of Paediatric Dentistry. 2012;22(2):85-91.	Excluded by title
363	Jälevik B, Klingberg GA. Dental treatment, dental fear and behaviour management problems in children with severe enamel hypomineralization of their permanent first molars. International Journal of Paediatric Dentistry. 2002;12(1):24-32.	Excluded by title
364	Jälevik B, Norén JG. Enamel hypomineralization of permanent first molars: A morphological study and survey of possible aetiological factors. International Journal of Paediatric Dentistry. 2000;10(4):278-89.	Excluded by title
365	Jang KT, Mejía FA, García-Godoy F. Dentin bond strength of packable composites using one-bottle adhesives. American Journal of Dentistry. 2000;13(6):308-10.	Excluded by title
366	Jarrett WM. Treatment plan and treated cases. American Journal of Orthodontics. 1948;34(11):938-53.	Excluded by title
367	Jasulaityte L, Veerkamp JS, Weerheijm KL. Molar incisor hypomineralization: review and prevalence data from the study of primary school children in Kaunas/Lithuania. Eur Arch Paediatr Dent. 2007;8(2):87-94.	Excluded by title
368	Jasulaityte L, Weerheijm KL, Veerkamp JS. Prevalence of molar-incisor-hypomineralisation among children participating in the Dutch National Epidemiological Survey (2003). European archives of paediatric dentistry : official journal of the European Academy of Paediatric Dentistry. 2008;9(4):218-23.	Excluded by title
369	Jathanna VR, Shetty SR, Hegde MN, Roopalekha Jathanna PN, Jathanna RV. Epidemiological survey on prevalence of missing first molar due to caries: A case study. Journal of Pharmaceutical Sciences and Research. 2015;8(9):1065-9.	Excluded by title
370	Jayaraman J. Adolescent Patient with Cystic Fibrosis Presents with Suspected Ludwig Angina. Dent Clin North Am. 2023;67(3):443-6.	Excluded by title
371	Jenkins FR, Nichol RE. Atypical retention of infraoccluded primary molars with permanent successor teeth. Eur Arch Paediatr Dent. 2008;9(1):51-5.	Excluded by title
372	Jensen ED, Smart G, Lee N, Tan J, Oliver K, Ha WN, et al. Prevalence and morphological features of molar-root incisor malformation in children attending a specialist paediatric dental unit. International Journal of Paediatric Dentistry. 2023;33(6):543-52.	Excluded by title
373	Jetpurwala M, Sawant KR, Jain PS, Dedhia SP. Parental Perception of the Importance of the Permanent First Molar in Their Children. J Dent Child (Chic). 2020;87(1):26-30.	Excluded by title
374	Jian KL, Zhang C, Shang ZC, Yang L, Kong LY. Eucalrobusone C suppresses cell proliferation and induces ROS-dependent mitochondrial apoptosis via the p38 MAPK pathway in hepatocellular carcinoma cells. Phytomedicine. 2017;25:71-82.	Excluded by title
375	Jiao J, Shi D, Cao ZQ, Meng HX, Lu RF, Zhang L, et al. Effectiveness of non-surgical periodontal therapy in a large Chinese population with chronic periodontitis. J Clin Periodontol. 2017;44(1):42-50.	Excluded by title
376	Jin Y, Cao W, Wu M, Yuan Y. Accurate fuzzy predictive models through complexity reduction based on decision of needed fuzzy rules. Neurocomputing. 2019;323:344-51.	Excluded by title
377	Johansson AK, Johansson A, Birkhed D, Omar R, Baghdadi S, Khan N, et al. Dental erosion associated with soft-drink consumption in young Saudi men. Acta Odontol Scand. 1997;55(6):390-7.	Excluded by title
378	Johansson AK. On dental erosion and associated factors. Swed Dent J Suppl. 2002(156):1-77.	Excluded by title
379	Jonsdottir J, Lencioni T, Gervasoni E, Crippa A, Anastasi D, Carpinella I, et al. Improved Gait of Persons With Multiple Sclerosis After Rehabilitation: Effects on Lower Limb Muscle Synergies, Push-Off, and Toe-Clearance. Frontiers in Neurology. 2020;11.	Excluded by title
380	Jorkjend L, Birkeland JM. Alveolar bone loss in the permanent first molars of Norwegian schoolchildren receiving systematic dental care. Community Dentistry and Oral Epidemiology. 1976;4(1):22-4.	Excluded by title
381	Juarez C. Defining the isotopic fingerprint in modern Mexican populations: Using strontium, carbon, nitrogen and oxygen to determine region of origin for deceased undocumented border crossers. American Journal of Physical Anthropology. 2010;141:137.	Excluded by title
382	Juarez C. Using geolocation at the US Mexico border isotopic fingerprinting in modern Mexican populations: Using strontium, carbon, and oxygen to determine region of origin for deceased undocumented border crossers. American Journal of Physical Anthropology. 2011;144:180-1.	Excluded by title
383	Jumlongras D, White GE. Bond strengths of composite resin and compomers in primary and permanent teeth. Journal of Clinical Pediatric Dentistry. 1997;21(3):223-9.	Excluded by title
384	Jung YJ, Choi H, Oh E. Effects of fine particulate matter and cigarette smoking on MPTP-induced dopaminergic neuronal cell death: Implication for Parkinson's disease. Movement Disorder. 2021;36(SUPPL 1):S334-S6.	Excluded by title
385	Kabil NS, Badran AS, Wassel MO. Effect of the addition of chlorhexidine and miswak extract on the clinical performance and antibacterial properties of conventional glass ionomer: an in vivo study. International Journal of Paediatric Dentistry. 2017;27(5):380-7.	Excluded by title
386	Kadiyala SV, Chidambaranathan K. Prevalence of primate and anterior spacing for primary dentition in 3 to 6 year old children in chennai. Research Journal of Pharmacy and Technology. 2018;11(12):5413-6.	Excluded by title
387	Kalender A, Oztan MD, Basmaci F, Aksoy U, Orhan K. CBCT evaluation of multiple idiopathic internal resorptions in permanent molars: Case report. BMC Oral Health. 2014;14(1).	Excluded by title
388	Kälin C, Paul SJ, Schärer P, Düggelin M, Mathys D, Guggenheim R. Evaluation of the interface between one-bottle bonding agents and dentin by cryopreparation and low-temperature scanning electron microscopy (LTSEM). A pilot study on perfused dentinal samples. J Dent. 1998;26(5-6):511-20.	Excluded by title
389	Kamma JJ, Lygidakis NA, Nakou M. Subgingival microflora and treatment in prepubertal periodontitis associated with chronic idiopathic neutropenia. Journal of Clinical Periodontology. 1998;25(9):759-65.	Excluded by title
390	Kanas RJ, Kanas SJ. Localized aggressive multiparous periodontitis: A newly documented entity. General Dentistry. 2011;59(4):292-301.	Excluded by title
391	Kandasamy S, Woods MG. Is orthodontic treatment without premolar extractions always non-extraction treatment? Aust Dent J. 2005;50(3):146-51.	Excluded by title
392	Kankaala TM, Virtanen JI, Larmas MA. Timing of first fillings in the primary dentition and permanent first molars of asthmatic children. Acta Odontologica Scandinavica. 1998;56(1):20-4.	Excluded by title
393	Kantaputra PN, Kapoor S, Verma P, Kaewgahya M, Kawasaki K, Ohazama A, et al. Al-Awadi-Raas-Rothschild syndrome with dental anomalies and a novel WNT7A mutation. European Journal of Medical Genetics. 2017;60(12):695-700.	Excluded by title
394	Kantaputra PN, Smith LJ, Casal ML, Kuptanon C, Chang Y-C, Nampoothiri S, et al. Oral manifestations in patients and dogs with mucopolysaccharidosis Type VII. American Journal of Medical Genetics Part A. 2019;179(3):486-93.	Excluded by title
395	Kantovitz KR, Pascon FM, Nociti Jr FH, Tabchoury CPM, Puppin-Rontani RM. Inhibition of enamel mineral loss by fissure sealant: An in situ study. Journal of Dentistry. 2013;41(1):42-50.	Excluded by title
396	Kanzow P, Wiegand A, Schwendicke F. Cost-effectiveness of repairing versus replacing composite or amalgam restorations. J Dent. 2016;54:41-7.	Excluded by title
397	Kashbour W, Gupta P, Worthington HV, Boyers D. Pit and fissure sealants versus fluoride varnishes for preventing dental decay in the permanent teeth of children and adolescents. Cochrane Database Syst Rev. 2020;11(11):Cd003067.	Excluded by title
398	Kato A, Ziegler A, Higuchi N, Nakata K, Nakamura H, Ohno N. Aetiology, incidence and morphology of the C-shaped root canal system and its impact on clinical endodontics. International Endodontic Journal. 2014;47(11):1012-33.	Excluded by title
399	Kaya MS, Akyuz S, Guclu B, Diracoglu D, Yarat A. Masticatory parameters of children with and without clinically diagnosed caries in permanent dentition. European Journal of Paediatric Dentistry. 2017;18(2):116-20.	Excluded by title
400	Kayhanian S, Ghazali N, Malins TJ, Gahir D. Long-term survival and management of childhood oral carcinoma cuniculatum. British Journal of Oral and Maxillofacial Surgery. 2015;53(10):e84.	Excluded by title
401	Kayipmaz S, Saricaoğlu ST, Sezgin OS. Unusual teeth transpositions: Two case reports. Oral Radiology. 2009;25(1):81-4.	Excluded by title
402	Kells BE, Linden GJ. Overhanging amalgam restorations in young adults attending a periodontal department. J Dent. 1992;20(2):85-9.	Excluded by title
403	Keszthelyi G, Szabó I. Attachment loss in primary molars. Journal of Clinical Periodontology. 1987;14(1):48-51.	Excluded by title
404	Ketley CE, Holt RD. Visual and radiographic diagnosis of occlusal caries in first permanent molars and in second primary molars. British Dental Journal. 1993;174(10):364-70.	Excluded by title
405	Khodadadi E, Khafri S. Epidemiological evaluation of DMFT of first permanent molar in 12 year old students of Babol city; Iran (2011-2012). Journal of Babol University of Medical Sciences. 2013;15(5):102-6.	Excluded by title
406	Khouja T, Smith KJ. Cost-effectiveness analysis of two caries prevention methods in the first permanent molar in children. J Public Health Dent. 2018;78(2):118-26.	Excluded by title
407	Kilpatrick N. New developments in understanding development defects of enamel: Optimizing clinical outcomes. Journal of Orthodontics. 2009;36(4):277-82.	Excluded by title
408	Kim HR, Nam SH, Kim HJ, Choi SY. Buccal Bifurcation Cyst: Two Case Reports and a Literature Review. J Clin Pediatr Dent. 2018;42(3):221-4.	Excluded by title
409	Kirzioğlu Z, Ertürk MS. Success of reinforced fiber material space maintainers. J Dent Child (Chic). 2004;71(2):158-62.	Excluded by title

410	Kocadereli I, Turgut MD. Management of occlusal and developmental disturbances resulting from an ankylosed maxillary second primary molar: case report. J Dent Child (Chic). 2003;70(2):178-81.	Excluded by title
411	Koch MJ, García-Godoy F. The clinical performance of laboratory-fabricated crowns placed on first permanent molars with developmental defects. Journal of the American Dental Association. 2000;131(9):1285-90.	Excluded by title
412	Komorowska A, Wróblewski M. Results of premolar grafting into the extraction wounds of the first permanent molars. Czasopismo stomatologiczne. 1981;34(4):413-21.	Excluded by title
413	Kondo S, Wakatsuki E, Shun-Te H, Sheng-Yen C, Shibazaki Y, Arai M. Comparison of the crown dimensions between the maxillary second deciduous molar and the first permanent molar. Okajimas Folia Anat Jpn. 1996;73(4):179-84.	Excluded by title
414	Kopperud SE, Pedersen CG, Espelid I. Treatment decisions on Molar-Incisor Hypomineralization (MIH) by Norwegian dentists - a questionnaire study. Bmc Oral Health. 2016;17.	Excluded by title
415	Kosyk M, Zimmerman C, Shakir S, Zapatero Z, Kalmar C, Nah HD, et al. Comprehensive mid-term outcomes following infant mandibular distraction osteogenesis. Cleft Palate-Craniofacial Journal. 2021;58(4 SUPPL):49.	Excluded by title
416	Koyuturk AE, Ozmen B, Tokay U, Tuloglu N, Erhan Sari M, Taha Sonmez T. Two-year follow-up of indirect posterior composite restorations of permanent teeth with excessive material loss in pediatric patients: A clinical study. Journal of Adhesive Dentistry. 2013;15(6):583-90.	Excluded by title
417	Kucukyilmaz E, Sener Y, Tosun G, Savas S. Periostitis ossificans managed with endodontic treatment. J Dent Child (Chic). 2015;82(1):53-6.	Excluded by title
418	Kumar Dash J, Mohapatra M, Mishra L. Extraoral inverted teeth eruption: A case report. Oral Surgery, Oral Medicine, Oral Pathology, Oral Radiology and Endodontology. 2004;98(1):37-9.	Excluded by title
419	Kumar H, Palamara J, Burrow MF, Manton DJ. Resin infiltration-taking the first steps to filling the holes in cheese molars. Ann R Australas Coll Dent Surg. 2012;21:120-3.	Excluded by title
420	Kupietzky A, Flaitz CM, Zeltser R. Eruption of a severely displaced second permanent molar following surgical removal of an odontoma. Pediatr Dent. 2003;25(4):378-82.	Excluded by title
421	Kupietzky A. Correction of ectopic eruption of permanent molars utilizing the brass wire technique. Pediatr Dent. 2000;22(5):408-12.	Excluded by title
422	Kurkutan E, Gundogar ZU, Keskin G. Prevalence and characteristics of ectopic eruption of the first permanent molar and predictive factors for clinical outcome. Journal of Oral Health and Oral Epidemiology. 2023;12(2):89-94.	Excluded by title
423	Kurol J, Bjerklin K. Ectopic eruption of maxillary first permanent molars: a review. ASDC J Dent Child. 1986;53(3):209-14.	Excluded by title
424	Kurol J, Bjerklin K. Treatment of children with ectopic eruption of the maxillary first permanent molar by cervical traction. Am J Orthod. 1984;86(6):483-92.	Excluded by title
425	Kurol J, Olson L. Ankylosis of primary molars-a future periodontal threat to the first permanent molars? European Journal of Orthodontics. 1991;13(5):404-9.	Excluded by title
426	Kuzmina IN, Kuzmina E, Ekstrand KR. Dental caries among children from Solntsevsky – a district in Moscow, 1993. Community Dentistry and Oral Epidemiology. 1995;23(5):266-70.	Excluded by title
427	Kwon SO, Lee S, Lee N. Treatment of combined primary impaction of primary second molars and permanent first molars. Pediatric Dentistry. 2016;38(7):E143-E7.	Excluded by title
428	la Monaca G, Cristalli MP, Pranno N, Galluccio G, Annibali S, Pippi R. First and second permanent molars with failed or delayed eruption: Clinical and statistical analyses. American Journal of Orthodontics and Dentofacial Orthopedics. 2019;156(3):355-64.	Excluded by title
429	Lam PP, Sardana D, Lo EC, Yiu CK. FISSURE SEALANT IN A NUTSHELL. EVIDENCE-BASED META-EVALUATION OF SEALANTS' EFFECTIVENESS IN CARIES PREVENTION AND ARREST. Journal of Evidence-Based Dental Practice. 2021;21(3).	Excluded by title
430	Lam WYH, Ho EHT, Pow EHN. Rehabilitation of molar-incisor hypomineralization (MIH) complicated with localized tooth surface loss: A case report. Quintessence International. 2014;45(5):377-9.	Excluded by title
431	Lang J, Birkenbeil S, Bock S, Heinrich-Weltzien R, Kromeyer-Hauschild K. Dental enamel defects in German medieval and early-modern-age populations. Anthropol Anz. 2016;73(4):343-54.	Excluded by title
432	Langowski N, Pilbrow V, Sagona A. Dietary trajectories and stable isotope analyses indicate marked diversity between neighboring sites of Samtavro and Tchkantiskhedi (Republic of Georgia, 1st-6th c. AD). American Journal of Physical Anthropology. 2015;156:198.	Excluded by title
433	Larsson E, Nordblom A. Thirteen-year-old boy with dentinogenesis imperfecta - pedodontic and orthodontic treatment. Swedish dental journal. 1981;5(5-6):213-7.	Excluded by title
434	Latcham NL, Powell RN, Jago JD, Seymour GJ, Aitken JF. A radiographic study of chronic periodontitis in 15 year old Queensland children. J Clin Periodontol. 1983;10(1):37-45.	Excluded by title
435	Lavanya S, Sujatha S. Detection of MB2 canal in maxillary primary second molar using cone beam computerized tomography (CBCT) – An in vitro study. Journal of Pharmaceutical Sciences and Research. 2016;8(4):220-3.	Excluded by title
436	Lavergne J, Petrovic AG. Inclinación de crecimiento mandibular y magnitud del movimiento distal de premolares luego de la extracción de los molares permanentes. Ortodoncia. 1987;51(101):7-15.	Excluded by title
437	Lazo R, Silva-Esteves F, Carpio C. Mantenedor de espacio intra-alveolar: tipo zapata distal: reporte de casos. Odontol pediatr (Lima). 2002;1(1):7-11.	Excluded by title
438	Leclercq M, Haghi P, Reiss T, Vennat E. Ultrastructure of dentine: A TEM investigation. Computer Methods in Biomechanics and Biomedical Engineering. 2021;24(SUPPL 1):S59-S60.	Excluded by title
439	Lee BS, Lai EHH, Liao KH, Lee CY, Hsieh KH, Lin CP. A Novel Polyurethane-based Root Canal-obturation Material and Urethane-Acrylate-based Root Canal Sealer-Part 2: Evaluation of Push-out Bond Strengths. Journal of Endodontics. 2008;34(5):594-8.	Excluded by title
440	Lee BS, Lin PY, Chen MH, Hsieh TT, Lin CP, Lai JY, et al. Tensile bond strength of Er,Cr:YSGG laser-irradiated human dentin and analysis of dentin-resin interface. Dental Materials. 2007;23(5):570-8.	Excluded by title
441	Lee BS, Lin YC, Chen SF, Chen SY, Chang CC. Influence of calcium hydroxide dressing and acid etching on the push-out bond strengths of three luting resins to root canal dentin. Clinical Oral Investigations. 2014;18(2):489-98.	Excluded by title
442	Lee DC, Oh JM, Choi H, Kim SW, Kim SW, Kim BG, et al. Eupatilin inhibits reactive oxygen species generation via Akt/NF-κB/MAPK signaling pathways in particulate matter-exposed human bronchial epithelial cells. Toxics. 2021;9(2):1-11.	Excluded by title
443	Lee HS, Kim SH, Kim SO, Choi BJ, Cho SW, Park W, et al. Microscopic analysis of molar-incisor malformation. Oral Surgery, Oral Medicine, Oral Pathology and Oral Radiology. 2015;119(5):544-52.	Excluded by title
444	Lee J, Chen JW, Omar S, Kwon SR, Meharry M. Evaluation of stain penetration by beverages in demineralized enamel treated with resin infiltration. Operative Dentistry. 2016;41(1):93-102.	Excluded by title
445	Lee J, Jang KT, Kim JW, Lee SH, Hahn SH, Kim CC. Effect of ultrasonic vibration on dentin bond strength and resin infiltration. Am J Dent. 2003;16(6):404-8.	Excluded by title
446	Lee J, Johnson J, Bister D, Chaudhary M, Khoshkhounejad G. Adherence to RCS recommendations for extraction of first permanent molars in a teaching hospital: To compensate or not to compensate? J Orthod. 2021;48(3):305-12.	Excluded by title
447	Lee YL, Li KC, Yiu CKY, Boyd DH, Ekambaram M. Evaluation of developmentally hypomineralised enamel after surface pretreatment with Papacarie Duo gel and different etching modes: an in vitro SEM and AFM study. European Archives of Paediatric Dentistry. 2022;23(1):117-31.	Excluded by title
448	Lehmann S, Sczyslo A, Froch-Cortis J, Rothschild MA, Thevis M, Andresen-Streichert H, et al. Organ distribution of diclazepam, pyrazolam and 3-fluorophenmetrazine. Forensic Sci Int. 2019;303:109959.	Excluded by title
449	Leibovitz S, Haviv Y, Zilberman U. The effect of Ekman-Westborg and Julin trait (generalized megadontia) and localized megadontia on tooth components size and ion content. Clin Oral Investig. 2021;25(3):833-9.	Excluded by title
450	Leite AP, Silva RG, da Cruzfilho AM, Pécora JD. In vitro study of cervical enamel projection in human molars. Brazilian dental journal. 1995;6(1):25-8.	Excluded by title
451	Leite FM, Dias RSC, Matos HR, Pereira TM, Oliveira MTS, MacIel LAS, et al. Association between skeletal muscular mass and anthropometric parameters in hypertense and diabetics attended in a renal disease prevention center. Journal of Clinical Hypertension. 2020;22(4):761.	Excluded by title
452	Lemos Rinaldi MR, Azeredo F, Martinelli de Lima E, Deon Rizzatto SM, Sameshima G, Macedo de Menezes L. Cone-beam computed tomography evaluation of bone plate and root length after maxillary expansion using tooth-borne and tooth-tissue-borne banded expanders. American Journal of Orthodontics and Dentofacial Orthopedics. 2018;154(4):504-16.	Excluded by title
453	León-Prados JA, Ahmaidi S, González-Jurado J, Weissland T. Acute effects of static passive vs contract-relax short-duration stretching on isometric and isokinetic performance in knee muscles: A single-group, pilot study. Isokinetics and Exercise Science. 2018;26(4):313-22.	Excluded by title
454	Leppäniemi A, Lukinmaa PL, Alaluusua S. Nonfluoride Hypomineralizations in the Permanent First Molars and Their Impact on the Treatment Need. Caries Research. 2001;35(1):36-40.	Excluded by title
455	Levine RS, Nugent ZJ, Rudolf MC, Sahota P. Dietary patterns, toothbrushing habits and caries experience of schoolchildren in West Yorkshire, England. Community Dent Health. 2007;24(2):82-7.	Excluded by title
456	Levit B. Pérdida de molar previo a la erupción del 1er. molar permanente. Manejo del mantenimiento del espacio (trad). Bol Asoc Argent Odontol Niños. 1992;21(1):26-7.	Excluded by title
457	Lewis PD. Canine retraction. American Journal of Orthodontics. 1970;57(6):543-60.	Excluded by title
458	Li M, Fang S, Wang X, Chen S, Cao L, Han J, et al. Peripheral Blood Leukocyte Detection Based on an Improved Detection Transformer Algorithm. Sensors (Basel). 2023;23(16).	Excluded by title
459	Li X, Cai H, Wu W, Si S, Zhu M. Exposure duration of ambient fine particulate matter determines the polarization of macrophages. Central European Journal of Urology. 2023;48(3):219-27.	Excluded by title
460	Lim WH, Chun YS. Orthodontic treatment combined with autotransplantation after removal of ameloblastoma. Am J Orthod Dentofacial Orthop. 2009;135(3):375-9.	Excluded by title
461	Lin H, Xin W, Chang S, Yang Q, Miao Q, Liu R, et al. Focus on hierarchical features: Soft-weighted hierarchical features network. Neurocomputing. 2023;516:182-93.	Excluded by title
462	Lin R, Hildebrand T, Donly KJ. In vitro remineralization associated with a bioerodible fluoridated resin and a fluoride varnish. American Journal of Dentistry. 2009;22(4):203-5.	Excluded by title
463	Lin YT, Chang LC. Space changes after premature loss of the mandibular primary first molar: a longitudinal study. J Clin Pediatr Dent. 1998;22(4):311-6.	Excluded by title
464	Lin YT, Lin WH, Lin YT. Immediate and six-month space changes after premature loss of a primary maxillary first molar. J Am Dent Assoc. 2007;138(3):362-8.	Excluded by title
465	Lin YT, Lin WH, Lin YTJ. Twelve-month space changes after premature loss of a primary maxillary first molar. International Journal of Paediatric Dentistry. 2011;21(3):161-6.	Excluded by title
466	Lin YT. Ectopically erupting mandibular first permanent molar: treatment of a case. J Clin Pediatr Dent. 1996;21(1):31-3.	Excluded by title
467	Lindemeyer RG, Glavich GG. Space maintainer for the loss of a permanent molar in the adolescent patient: report of case. ASDC J Dent Child. 1996;63(3):213-5.	Excluded by title
468	Lindsten R, Ogaard B, Larsson E. Difference in dental lateral arch length between 9-year-olds born in the 1960s and the 1980s. American journal of orthodontics and dentofacial orthopedics : official publication of the American Association of Orthodontists, its constituent societies, and the American Board of Orthodontics. 2000;117(6):663-8.	Excluded by title
469	Litzenburger F, Lederer A, Kollmuss M, Hickel R, Kunzelmann K-H, Heck K. Near-infrared transillumination with high dynamic range imaging for occlusal caries detection in vitro. Lasers in Medical Science. 2020;35(9):2049-58.	Excluded by title
470	Liu G, Li W, Li L, Jiang X. The value of CT image-based texture analysis for differentiating renal primary undifferentiated pleomorphic sarcoma from three subtypes of renal cell carcinoma. International Journal of Clinical and Experimental Medicine. 2017;10(9):13526-33.	Excluded by title
471	Liu J, Liu Y, Li S, Ying S, Zheng L, Zhao Z. Artificial intelligence-aided detection of ectopic eruption of maxillary first molars based on panoramic radiographs. J Dent. 2022;125:104239.	Excluded by title
472	Livas C, Halazonetis DJ, Booij JW, Pandis N, Tu YK, Katsaros C. Maxillary sinus floor extension and posterior tooth inclination in adolescent patients with Class II Division 1 malocclusion treated with maxillary first molar extractions. American Journal of Orthodontics and Dentofacial Orthopedics. 2013;143(4):479-85.	Excluded by title
473	Lizarelli Rde F, Moriyama LT, Bagnato VS. Ablation of composite resins using Er:YAG laser--comparison with enamel and dentin. Lasers Surg Med. 2003;33(2):132-9.	Excluded by title
474	Lizio G, Corinaldesi G, Bianchi A, Marchetti C. Successful resolution of juvenile paradental cysts after marsupialization in five consecutive patients. Australian Dental Journal. 2011;56(4):427-32.	Excluded by title
475	Lo YF, Crispin A, Kesslerc A, Hickeld R, Kühnisch J. What is an appropriate etching time for sealant application on permanent molars? Results from a meta-analysis. Journal of Adhesive Dentistry. 2019;21(6):487-95.	Excluded by title
476	Loevy HT, Shore SW. Dental maturation in hemifacial microsomia. J Craniofac Genet Dev Biol Suppl. 1985;1:267-72.	Excluded by title
477	Lopez R. Root Resorption in the Furcation Area: A Differential Diagnostic Consideration. Journal of Periodontology. 2010;81(11):1698-702.	Excluded by title

478	Loureiro H, Monteiro M, Lajes M, Silva M, Martins R, Veríssimo M. Relation between hand grip strength and body composition in the elderly. European Geriatric Medicine. 2018;9:S304.	Excluded by title
479	Lunt DA. Evidence of Tooth Extraction in a Cypriot Mandible of the Hellenistic or Early Roman Period, C.150 Bc to 100 Ad. British Dental Journal. 1992;173(7):242-3.	Excluded by title
480	Lupi-Pegurier L, Bertrand MF, Muller-Bolla M, Rocca JP, Bolla M. Comparative study of microleakage of a pit and fissure sealant placed after preparation by Er: YAG laser in permanent molars. Journal of Dentistry for Children. 2003;70(2):134-8.	Excluded by title
481	Lygidakis NA, Oulis KI. A comparison of Fluroshield with Delton fissure sealant: Four year results. Pediatric Dentistry. 1999;21(7):429-31.	Excluded by title
482	Mabrouk R, Baccouche C, Frih N. Morphometric Analysis of Furcation Areas of Multirooted Teeth in a Tunisian Population. International Journal of Dentistry. 2020;2020.	Excluded by title
483	Macedo BGd, Oliveira HSCd, Paula MVd, Gomes GdC, Antunes CMdF. Association between inflammatory mediators, grip strength and mobility in community-dwelling elderly. Fisioter Mov (Online). 2018;31:e003132-e.	Excluded by title
484	Madrid CC, De Pauli Paglioni M, Line SR, Vasconcelos KG, Brandão TB, Lopes MA, et al. Structural Analysis of Enamel in Teeth from Head-and-Neck Cancer Patients Who Underwent Radiotherapy. Caries Research. 2017;51(2):119-28.	Excluded by title
485	Mahmood AA, Hossain R, Bhattacharyya S, Privat K, Sahajwalla V. Rapid transformation of the metal-polymer laminated packaging materials into ceramic carbide reinforced Al-alloy. Resources, Conservation and Recycling. 2021;167.	Excluded by title
486	Mahoney EK. The treatment of localised hypoplastic and hypomineralised defects in first permanent molars. N Z Dent J. 2001;97(429):101-5.	Excluded by title
487	Maita Castañeda LM. Tratamiento ortodóncico de premolar superior impactada por molar decidua anquilosada. Kiru. 2008;5(2):116-22.	Excluded by title
488	Maj G, Alleva F, Lucchese FP. Changes in length and width of the mandibular arch from the mixed dentition to the completion of the permanent dentition. European Journal of Orthodontics. 1979;1(4):259-64.	Excluded by title
489	Malden N, Kaye-Wilson LG, Hudson I, Valentine D. Transient uniocular blindness after dental extraction under general anaesthetic. Br Dent J. 1993;174(9):334-5.	Excluded by title
490	Mandari GJ, Frencken JE, van't Hof MA. Six-year success rates of occlusal amalgam and glass-ionomer restorations placed using three minimal intervention approaches. Caries Res. 2003;37(4):246-53.	Excluded by title
491	Manikandaselvi S, Brindha P. Chemical standardization studies on Capparis spinosa L. International Journal of Pharmacy and Pharmaceutical Sciences. 2014;6(SUPPL 1):47-54.	Excluded by title
492	Manuja N, Pandit IK, Srivastava N, Gugnani N, Nagpal R. Comparative evaluation of shear bond strength of various esthetic restorative materials to dentin: An in vitro study. Journal of Indian Society of Pedodontics and Preventive Dentistry. 2011;29(1):7-13.	Excluded by title
493	Mardal M, Miserez B, Bade R, Portolés T, Bischoff M, Hernández F, et al. 3-Fluorophenmetrazine, a fluorinated analogue of phenmetrazine: Studies on in vivo metabolism in rat and human, in vitro metabolism in human CYP isoenzymes and microbial biotransformation in Pseudomonas Putida and wastewater using GC and LC coupled to (HR)-MS techniques. J Pharm Biomed Anal. 2016;128:485-95.	Excluded by title
494	Marks D, Owens BM, Johnson WW. Effect of adhesive agent and fissure morphology on the in vitro microleakage and penetrability of pit and fissure sealants. Quintessence International. 2009;40(9):763-72.	Excluded by title
495	Marro F, O'Toole S, Bernabé E, Bartlett D, Aránguiz V. Associated risk factors with quantitative erosive tooth wear progression. Journal of Dentistry. 2022;123.	Excluded by title
496	Martin G, Ricucci D, Gibbs JL, Lin LM. Histological findings of revascularized/revitalized immature permanent molar with apical periodontitis using platelet-rich plasma. J Endod. 2013;39(1):138-44.	Excluded by title
497	Martin-De Las Heras S, Valenzuela A, Overall CM. The matrix metalloproteinase gelatinase A in human dentine. Archives of Oral Biology. 2000;45(9):757-65.	Excluded by title
498	Martínez MV, Tomich D, Ucero CT, Spina MN. La flexicorticotomia como procedimiento para la mesialización de un molar inferior en pacientes adultos: reporte de un caso. Acta odontol venez. 2013;51(3).	Excluded by title
499	Martins JFB, Hagay S, Herbst SR, Falk S. Cost-effectiveness analysis of full versus selective root canal retreatment. International Endodontic Journal. 2023;56(11):1319-27.	Excluded by title
500	Mastora A, Vadiakas G, Agouropoulos A, Gartagani-Panagiotopoulou P, Gemou Engesaeth V. Developmental defects of enamel in first permanent molars associated with use of asthma drugs in preschool aged children: A retrospective case-control study. European Archives of Paediatric Dentistry. 2017;18(2):105-11.	Excluded by title
501	Matsuzaki H, Yanagi Y, Katase N, Nagatsuka H, Hara M, Ashida M, et al. Conditions inhibiting eruption of permanent first molars. Pediatric Dentistry. 2013;35(1):67-70.	Excluded by title
502	Mattar RE, Sulimany AM, Binsaleh SS, Hamdan HM, Al-Majed IM. Evaluation of fissure sealant retention rates using Isolite in comparison with rubber dam and cotton roll isolation techniques: A randomized clinical trial. International Journal of Paediatric Dentistry. 2023;33(1):12-9.	Excluded by title
503	Matus D, Cantín M. Evaluación de la frecuencia, ubicación y tipos de itsmos en raíces mesiobucales de primeros molares maxilares: un estudio ex vivo. Int j morphol. 2016;34(2):804-10.	Excluded by title
504	Mazhari F, Mehrabkhani M, Sadeghi S, Malekabadi KS. Effect of bevelling on marginal microleakage of buccal-surface fissure sealants in permanent teeth. Eur Arch Paediatr Dent. 2009;10(4):241-3.	Excluded by title
505	Mazhari F, Valizadeh M. Distal guide as a substitute for distal shoe space maintainer: a case series study. J Clin Pediatr Dent. 2022;46(6):58-62.	Excluded by title
506	Mazzawi JM, Henson T, Donly KJ. Inhibition of enamel demineralization by a bioerodible fluoridated resin. American Journal of Dentistry. 2007;20(2):134-6.	Excluded by title
507	Mc Cafferty J, Al Awadi E, O'Connell AC. Case report: Management of severe posterior open bite due to primary failure of eruption. European archives of paediatric dentistry : official journal of the European Academy of Paediatric Dentistry. 2010;11(3):155-8.	Excluded by title
508	McKay GS. The histology and microbiology of acute occlusal dentine lesions in human permanent molar teeth. Archives of Oral Biology. 1976;21(1):51-8.	Excluded by title
509	McKenna EF, Grundy GE. Glass ionomer cement fissure sealants applied by operative dental auxiliaries — retention rate after one year. Australian Dental Journal. 1987;32(3):200-3.	Excluded by title
510	McKnight-Hanes C, Myers DR, Salama FS, Thompson WO, Barenie JT. Comparing treatment options for occlusal surfaces utilizing an invasive index. Pediatr Dent. 1990;12(4):241-5.	Excluded by title
511	McMullan RE, Richardson A. Spontaneous changes in the rotation of premolar teeth from eruption until the established dentition. European Journal of Orthodontics. 1991;13(5):392-6.	Excluded by title
512	McNamara CM, Foley T, O'Sullivan VR, Crowley N, McConnell RJ. External resorption presenting as an intracoronal radiolucent lesion in a pre-eruptive tooth. Oral Dis. 1997;3(3):199-201.	Excluded by title
513	Medina W, Hurtig AK, San Sebastián M, Quizhpe E, Romero C. Dental caries in 6-12-year-old indigenous and non-indigenous schoolchildren in the amazon basin of ecuador. Brazilian Dental Journal. 2008;19(1):83-6.	Excluded by title
514	MejÀRe I, MjÖR IA. Glass ionomer and resin-based fissure sealants: a clinical study. European Journal of Oral Sciences. 1990;98(4):345-50.	Excluded by title
515	Mejàre I, Stenlund H. Caries rates for the mesial surface of the first permanent molar and the distal surface of the second primary molar from 6 to 12 years of age in Sweden. Caries Res. 2000;34(6):454-61.	Excluded by title
516	Mekari A, Almarrawi A, Splieth CH. Autotransplantation of an immature mandibular third molar to a recipient site with periradicular lesion: a case report. Quintessence Int. 2023;54(3):228-33.	Excluded by title
517	Meller C, Reichenmiller K, Schwahn C, Samietz S, Blunck U. Resin-based pit-and-fissure sealants: Microleakage reduction and infiltration enhancement using a bonding agent. Journal of Adhesive Dentistry. 2015;17(1):59-65.	Excluded by title
518	Melo FGCd, Cavalcanti AL, Fontes LdBC, Garcia AFG. Perda precoce de molares permanentes e fatores associados em escolares de 9, 12 e 15 anos da rede pública municipal de Campina Grande, estado da Paraíba, Brasil. Acta sci, Health sci. 2011;33(1):99-105.	Excluded by title
519	Memis S, Bas Z. Multiple dens invaginatus in Wilson's disease: A case report. Australian Endodontic Journal. 2021;47(3):679-83.	Excluded by title
520	Mendonça FL, Di Leone CCL, Grizzo IC, Cruvinel T, de Oliveira TM, Navarro MFDL, et al. Simplified occlusal replica adapted technique with glass ionomer cement for molar-incisor hypomineralization–affected molars: An 18-month follow-up. Journal of the American Dental Association. 2020;151(9):678-83.	Excluded by title
521	Mendoza-Hernández Y, Morales-Chávez M. Caries and premature loss of the first permanent molar in grade school children, and parents' knowledge level, in Vargas state, Venezuela. J oral res (Impresa). 2019;8(2):166-72.	Excluded by title
522	Meng M, Zhou X, Zhang Q, Zou J. Management of ectopically erupting permanent molars in a seven-year-old girl: A case report. J Pak Med Assoc. 2021;71(12):2809-11.	Excluded by title
523	Messer LB, Cline JT. Relative caries experience of sealed versus unsealed permanent posterior teeth: a three-year study. ASDC journal of dentistry for children. 1980;47(3):175-82.	Excluded by title
524	Meza Ruiz MA, Moreira Recinos CR. Evaluación in vitro de un material experimental a base de papaya 2r2m1 a diferentes concentraciones para remoción quimico-mecanica de dentina infectada. 2011. p. 40-.	Excluded by title
525	Mickenautsch S, Yengopal V. Caries-Preventive Effect of High-Viscosity Glass Ionomer and Resin-Based Fissure Sealants on Permanent Teeth: A Systematic Review of Clinical Trials. PLoS One. 2016;11(1):e0146512.	Excluded by title
526	Mickenautsch S, Yengopal V. Validity of sealant retention as surrogate for caries prevention--a systematic review. PLoS One. 2013;8(10):e77103.	Excluded by title
527	Milgrom P, Tut OK. Evaluation of Pacific Islands Early Childhood Caries Prevention Project: Republic of the Marshall Islands. J Public Health Dent. 2009;69(3):201-3.	Excluded by title
528	Mishra S, Mohanty S. Distal shoe space maintainer for the premature loss of primary second molar in a 5-year-old patient: A case report. Indian Journal of Forensic Medicine and Toxicology. 2020;14(4):8976-9.	Excluded by title
529	Mistry M, Zhu S, Moazzez R, Donaldson N, Bartlett DW. Effect of Model Variables on in vitro Erosion. Caries Res. 2015;49(5):508-14.	Excluded by title
530	Mitropoulos C. DMFS(e) index for selection of clinical trial subjects. Community Dentistry and Oral Epidemiology. 1985;13(1):30-2.	Excluded by title
531	Modesto A, Miranda DKBd, Bastos EPdS, Asturian C, Garcia ES. Prevalência da perda do primeiro molar permanente. Rev bras odontol. 1993;50(3):52-4.	Excluded by title
532	Molaasadolah F, Hosseinipour ZS, Afzali F, Parhizkar A, Poorzandpoush K. The effect of two calcium phosphate-containing agents on the enamel resistance of permanent molars to demineralization: An experimental study. Clin Exp Dent Res. 2022;8(6):1533-9.	Excluded by title
533	Morgan CR, Rodd HD, Clayton N, Boissonade FM. Changes in proteinase-activated receptor 2 expression in the human tooth pulp in relation to caries and pain. Journal of Oral and Facial Pain and Headache. 2009;23(3):265-74.	Excluded by title
534	Morita M, Kimura M, Nagashima T, Komoriyama H, Kiyoto K, Maeda C, et al. A case of unresectable advanced gastric cancer successfully treated with chemoimmunotherapy (low-dose FPM/OK-432 therapy). Biotherapy. 1998;12(5):803-5.	Excluded by title
535	Moscovich H, Creugers NH, Jansen JA, Wolke JG. In vitro dentine hardness following gamma-irradiation and freezing. J Dent. 1999;27(7):503-7.	Excluded by title
536	Moscovich H, Creugers NH. The novel use of extracted teeth as a dental restorative material--the 'Natural Inlay'. J Dent. 1998;26(1):21-4.	Excluded by title
537	Movahhedian N, Adibi S, Tavakoli HS, Baseri H. How does triangular-shaped radiolucency affect caries diagnosis? Oral Radiology. 2017;33(1):32-7.	Excluded by title
538	Mros ST, Berglundh T. Aggressive periodontitis in children: A 14-19-year follow-up. Journal of Clinical Periodontology. 2010;37(3):283-7.	Excluded by title
539	Mulic A, Cehajic E, Tveit AB, Stenhagen KR. How serious is molar incisor hypomineralisation (MIH) among 8- and 9-year-old children in Bosnia-Herzegovina? A clinical study. European Journal of Paediatric Dentistry. 2017;18(2):153-7.	Excluded by title
540	Muller-Bolla M, Courson F, Lupi-Pégurier L, Tardieu C, Mohit S, Staccini P, et al. Effectiveness of Resin-Based Sealants with and without Fluoride Placed in a High Caries Risk Population: Multicentric 2-Year Randomized Clinical Trial. Caries Research. 2018;52(4):312-22.	Excluded by title
541	Nagasiri R, Chitmongkolsuk S. Long-term survival of endodontically treated molars without crown coverage: a retrospective cohort study. J Prosthet Dent. 2005;93(2):164-70.	Excluded by title
542	Nagpal R, Manuja N, Pandit IK. Adhesive bonding to pulp chamber dentin after different irrigation regimens. Journal of investigative and clinical dentistry. 2015;6(4):287-93.	Excluded by title
543	Nagpal R, Manuja N, Pandit IK. Effect of ethanol wet bonding technique on the durability of resin- dentin bond with contemporary adhesive systems. The Journal of clinical pediatric dentistry. 2015;39(2):133-42.	Excluded by title
544	Nagpal R, Manuja N, Pandit IK. Effect of proanthocyanidin treatment on the bonding effectiveness of adhesive restorations in pulp chamber. Journal of Clinical Pediatric Dentistry. 2013;38(1):49-53.	Excluded by title

545	Nascimento GC, Bortolanza M, Bariotto K, Leite-Panissi C, Del Bel E. Effects of L-DOPA and doxycycline administration on nociceptive responses in an animal model of Parkinson's disease. Journal of Cerebral Blood Flow and Metabolism. 2017;37(1):480.	Excluded by title
546	Nazir M, Walsh T, Mandall NA, Matthew S, Fox D. Banding versus bonding of first permanent molars: a multi-centre randomized controlled trial. J Orthod. 2011;38(2):81-9.	Excluded by title
547	Neely AL. Prevalence of juvenile periodontitis in a circumpubertal population. J Clin Periodontol. 1992;19(6):367-72.	Excluded by title
548	Neuhaus KW, Jasarevic E, Lussi A. Impact of Different Illumination Conditions on Visual Caries Detection with ICDAS. Caries Research. 2015;49(6):633-6.	Excluded by title
549	Ngo HC, Mount G, Mc Intyre J, Tuisuva J, Von Doussa RJ. Chemical exchange between glass-ionomer restorations and residual carious dentine in permanent molars: an in vivo study. J Dent. 2006;34(8):608-13.	Excluded by title
550	Niknejad E, Alinejad V. Factors affecting the duration of decay of the first permanent molar tooth. Research Journal of Medical Sciences. 2016;10(2):36-8.	Excluded by title
551	Nogueira AJdS, Athayde Neto MDd, Hoshino N, Gillet AVM. Comprometimento do primeiro molar permanente após 1 ano de sua erupção. Rev odontopediatr. 1995;4(3):135-45.	Excluded by title
552	Nogueira VKC, Mendes Soares IP, Fragelli CMB, Boldieri T, Manton DJ, Bussaneli DG, et al. Structural integrity of MIH-affected teeth after treatment with fluoride varnish or resin infiltration: An 18-Month randomized clinical trial. J Dent. 2021;105:103570.	Excluded by title
553	Nopnakeepongsa W, Jantarat J, Surarit R, Smutkeeree A. Assessment of root dentin pH changes in primary and permanent molars with different types of calcium hydroxide intracanal medication. Pediatric Dental Journal. 2019;29(1):23-9.	Excluded by title
554	Norlund A, Axelsson S, Dahlén G, Espelid I, Mejàre I, Tranæus S, et al. Economic aspects of the detection of occlusal dentine caries. Acta Odontologica Scandinavica. 2009;67(1):38-43.	Excluded by title
555	Nouri MR, Kennedy DB. Optimal fit of chairside-fabricated distal shoe space maintainer. Eur Arch Paediatr Dent. 2013;14(5):351-4.	Excluded by title
556	Ntaoutidou S, Arhakis A, Tolidis K, Kotsanos N. Clinical evaluation of a surface pre-reacted glass (S-PRG) filler-containing dental sealant placed with a self-etching primer/adhesive. European Archives of Paediatric Dentistry. 2018;19(6):431-7.	Excluded by title
557	Nytun RB, Raadal M, Espelid I. Diagnosis of dentin involvement in occlusal caries based on visual and radiographic examination of the teeth. Scand J Dent Res. 1992;100(3):144-8.	Excluded by title
558	O'Connell AC, Puck JM, Grimbacher B, Facchetti F, Majorana A, Gallin JI, et al. Delayed eruption of permanent teeth in hyperimmunoglobulinemia E recurrent infection syndrome. Oral Surgery, Oral Medicine, Oral Pathology, Oral Radiology, and Endodontics. 2000;89(2):177-85.	Excluded by title
559	Ogidan O. Ectopic eruption of maxillary first permanent molar: a case report. Afr Dent J. 1995;9:34-7.	Excluded by title
560	Oh NY, Nam SH, Lee JS, Kim HJ. Delayed Spontaneous Eruption of Severely Infraoccluded Primary Second Molar: Two Case Reports. J Clin Pediatr Dent. 2020;44(3):185-9.	Excluded by title
561	Ohtawa Y, Ichinohe S, Kimura E, Hashimoto S. Erupted complex odontoma delayed eruption of permanent molar. Bull Tokyo Dent Coll. 2013;54(4):251-7.	Excluded by title
562	Olgen IC, Sonmez H, Bezgin T. Effects of different remineralization agents on MIH defects: a randomized clinical study. Clinical Oral Investigations. 2022;26(3):3227-38.	Excluded by title
563	Oliveira FSd. Selamento de fossas e fissuras: avaliação longitudinal da retenção e da eficácia na inibição da cárie oclusal do primeiro molar permanente. 2002. p. xxi,197-xxi,.	Excluded by title
564	Oliveira LAd, Pinto CMPX, Naves Neto VA. Cementoblastoma benigno na mandíbula: Relato de caso. Rev cir traumatol buco-maxilo-fac. 2013;13(3):49-54.	Excluded by title
565	Oliver K, Messer LB, Manton DJ, Kan K, Ng F, Olsen C, et al. Distribution and severity of molar hypomineralisation: trial of a new severity index. Int J Paediatr Dent. 2014;24(2):131-51.	Excluded by title
566	Oliver RG, Richmond S, Hunter B. Submerged permanent molars: Four case reports. British Dental Journal. 1986;160(4):128-30.	Excluded by title
567	Oliver RG. Orthodontics in general practice. Declining caries and the role of the first permanent molar. Dent Update. 1986;13(10):493-8.	Excluded by title
568	Oliver SJ, Dummer PM, Oliver RG, Hicks R, Addy M, Kingdon A, et al. The relationship between loss of first permanent molar teeth and the prevalence of caries and restorations in adjacent teeth: a study of 15-16- year-old children. J Dent. 1988;16(4):155-9.	Excluded by title
569	Olmo-González B, Moreno-López R, Ribera-Uribe M. Dental management strategies for Molar Incisor Hypomineralization. Pediatric Dental Journal. 2020;30(3):139-54.	Excluded by title
570	Oltramari PVP, Bespalez Neto R. Ortodontia infantil: manejo de espaço na dentadura mista com alinhadores. Ortho Sci, Orthod sci pract. 2023;16(62):106-16.	Excluded by title
571	Omar SM, Meguid SH. The role of the fissure morphology in caries prevalence of the first permanent molar in Libyan children. J Indian Soc Pedod Prev Dent. 1998;16(4):107-10.	Excluded by title
572	Onda S, Minemura R, Masaki T, Funatsu S. Shape and number of the roots of the permanent molar teeth. Bull Tokyo Dent Coll. 1989;30(4):221-31.	Excluded by title
573	Ong JC, De Silva RK, Tong DC. Retrieval of a root fragment from the maxillary sinus--an appreciation of the Caldwell-Luc procedure. N Z Dent J. 2007;103(1):14-6.	Excluded by title
574	Opinya GN, Kaimenyi JT, Meme JS. Oral findings in Fanconi's anemia. A case report. Journal of periodontology. 1988;59(7):461-3.	Excluded by title
575	Opydo-Szymaczek J, Gerreth K. Developmental Enamel Defects of the Permanent First Molars and Incisors and Their Association with Dental Caries in the Region of Wielkopolska, Western Poland. Oral Health Prev Dent. 2015;13(5):461-9.	Excluded by title
576	Orellana Centeno JE, González Quintero JS, Nava Calvillo JF, Nava Zarate N, Orellana Centeno M, Ponce Palomares SM. Estudio epidemiológico de la incidencia de caries del órgano dentario 46 como primer diente afectado en la dentición permanente. Acta odontol venez. 2012;50(2).	Excluded by title
577	Ortiz MIG, Ribeiro MES, Lima DANL, Silva CM, Loretto SC, da Silva e Souza Júnior MH. COMPLIANCE OF RANDOMIZED CLINICAL TRIALS ON DENTAL CARIES PREVENTION METHODS WITH THE CONSORT STATEMENT: A SYSTEMATIC REVIEW. Journal of Evidence-Based Dental Practice. 2021;21(2).	Excluded by title
578	Orton-Gibbs S, Crow V, Orton HS. Eruption of third permanent molars after the extraction of second permanent molars. Part 1: Assessment of third molar position and size. American Journal of Orthodontics and Dentofacial Orthopedics. 2001;119(3):226-38.	Excluded by title
579	Orton-Gibbs S, Orton S, Orton H. Eruption of third permanent molars after the extraction of second permanent molars. Part 2: Functional occlusion and periodontal status. American Journal of Orthodontics and Dentofacial Orthopedics. 2001;119(3):239-44.	Excluded by title
580	Oulis C, Berdousis E, Vadiakas G, Goumenos G. Garre's osteomyelitis of an unusual origin in a 8-year-old child. A case report. Int J Paediatr Dent. 2000;10(3):240-4.	Excluded by title
581	Oulis CJ, Berdouses ED. Fissure sealant retention and caries development after resealing on first permanent molars of children with low, moderate and high caries risk. Eur Arch Paediatr Dent. 2009;10(4):211-7.	Excluded by title
582	Oulis CJ, Tsinidou K, Vadiakas G, Mamai-Homata E, Polychronopoulou A, Athanasouli T. Caries prevalence of 5, 12 and 15-year-old Greek children: A national pathfinder survey. Community Dental Health. 2012;29(1):29-32.	Excluded by title
583	Owen DG. The incidence and nature of space closure following the premature extraction of deciduous teeth: A literature survey. American Journal of Orthodontics. 1971;59(1):37-49.	Excluded by title
584	Owens BM, Kitchens M. The erosive potential of soft drinks on enamel surface substrate: An in vitro scanning electron microscopy investigation. Journal of Contemporary Dental Practice. 2007;8(7):011-20.	Excluded by title
585	Owens BM, Sharp HK, Fourmy EE, Phebus JG. Effect of occlusal calculus utilized as a potential "biological sealant" in special needs patients with gastric feeding tubes: a qualitative in vitro contrast to pit and fissure sealant restorations. General Dentistry. 2016;64(4):24-9.	Excluded by title
586	Oyedele TA, Fadeju AD, Adeyemo YI, Nzomiwu CL, Ladeji AM. Impact of oral hygiene and socio-demographic factors on dental caries in a suburban population in Nigeria. European Archives of Paediatric Dentistry. 2018;19(3):155-61.	Excluded by title
587	Ozmeriç N, Bal B, Baloş K, Berker E, Bulut S. The correlation of gingival crevicular fluid interleukin-8 levels and periodontal status in localized juvenile periodontitis. J Periodontol. 1998;69(11):1299-304.	Excluded by title
588	Pagaria S, Singh BD, Dubey A, Avinash A. Molar incisor hypomineralisation: A review. Kathmandu University Medical Journal. 2015;13(51):281-5.	Excluded by title
589	Paleczny G. Treatment of the ankylosed mandibular permanent first molar: a case study. J Can Dent Assoc. 1991;57(9):717-9.	Excluded by title
590	Palencar AJ. Intrusion and uprighting teeth for fixed prosthesis with temporary anchorage device. Int J Orthod Milwaukee. 2012;23(3):15-8.	Excluded by title
591	Palesik B, Šileikytė K, Griškevičius J, Stonkus R, Šidlauskas A, Lopatienė K. Impact of temperature changes to the adhesion strength of molar tubes: an in vitro study. BMC Oral Health. 2022;22(1).	Excluded by title
592	Palma C, Coelho A, González Y, Cahuana A. Failure of eruption of first and second permanent molars. Journal of Clinical Pediatric Dentistry. 2003;27(3):239-45.	Excluded by title
593	Papageorgiou SN, Dimitraki D, Kotsanos N, Bekes K, van Waes H. Performance of pit and fissure sealants according to tooth characteristics: A systematic review and meta-analysis. J Dent. 2017;66:8-17.	Excluded by title
594	Parentoni AN, Lustosa LP, Santos KDd, Sá LF, Ferreira FO, Mendonça VA. Comparação da força muscular respiratória entre os subgrupos de fragilidade em idosas da comunidade. Fisioter pesqui. 2013;20(4):361-6.	Excluded by title
595	Park S, Byun S, Kim J, Yang B, Oh S. Treatment of Molar Incisor Malformation and the short term follow-up: Case reports. European Journal of Paediatric Dentistry. 2020;21(3):238-42.	Excluded by title
596	Pasqualini D, Bianchi CC, Paolino DS, Mancini L, Cemenasco A, Cantatore G, et al. Computed micro-tomographic evaluation of glide path with nickel-titanium rotary pathfile in maxillary first molars curved canals. Journal of Endodontics. 2012;38(3):389-93.	Excluded by title
597	Passos TGR, Tannure PN, Imparato JCP, Pintor AVB. Molar-incisor malformation: a narrative review. Rev Cient CRO-RJ (Online). 2020;5(3):2-12.	Excluded by title
598	Passy L, Silva S, Brock I, Wells G, Cox A, Danson S. Assessing melanoma BRAF status through ddPCR of cfDNA. Postgraduate Medical Journal. 2019;95(1130):686-7.	Excluded by title
599	Pastrav M, Viorica T, Pastrav O. Different types of intraoral anchorage appliances used in orthodontic treatments and their efficiency in cases with first permanent molar extractions. Clujul Medical. 2015;88:S196.	Excluded by title
600	Patel M, Schultz K, Rosenfeld E. The earliest known reported occurrence of dentigerous cyst in a six-month-old child. Int J Oral Maxillofac Surg. 2022;51(12):1535-7.	Excluded by title
601	Patiño Villada FA, Arboleda Franco SA, Paz Fernández JAd. Sarcopenia in community-dwelling persons over 60 years of age from a northern Spanish city: relationship between diagnostic criteria and association with the functional performance. Nutr hosp. 2015;31(5):2154-60.	Excluded by title
602	Patricia Pontigo-Loyola A, de Lourdes Marquez-Corona M, Minaya-Sanchez M, Eduardo Lucas-Rincon S, Fernando Casanova-Rosado J, Luis Robles-Minaya J, et al. Correlation between the caries status of the first permanent molars and the overall DMFT Index: A cross-sectional study. Medicine. 2020;99(5).	Excluded by title
603	Pavlič A, Vrecl M, Jan J, Bizjak M, Nemec A. Case report of a molar-root incisor malformation in a patient with an autoimmune lymphoproliferative syndrome. BMC Oral Health. 2019;19(1):49.	Excluded by title
604	Pawar A, Thakur B, Machado R, Kwak S, Kim HC. An In-Vivo cone-beam computed tomography analysis of root and canal morphology of maxillary first permanent molars in an Indian population. Indian Journal of Dental Research. 2021;32(1):104-9.	Excluded by title
605	Peiris HRD, Pitakotuwage TN, Takahashi M, Sasaki K, Kanazawa E. Root canal morphology of mandibular permanent molars at different ages. International Endodontic Journal. 2008;41(10):828-35.	Excluded by title
606	Penchas J, Peretz B, Becker A. The dilemma of treating severely decayed first permanent molars in children: to restore or to extract. ASDC journal of dentistry for children. 1994;61(3):199-205.	Excluded by title
607	Penha KJDS, Roma FRVDO, Filho EMM, Ribeiro CCC, Firoozmand LM. Bioactive self-etching sealant on newly erupted molars: A split-mouth clinical trial. Journal of Dentistry. 2021;115.	Excluded by title
608	Pereira AC, Eggertsson H, González-Cabezas C, Zero DT, Eckert GJ, Mialhe FL. Quantitative light-induced fluorescence (QLF) in relation to other technologies and conventional methods for detecting occlusal caries in permanent teeth. Braz j oral sci. 2011;10(1):27-32.	Excluded by title
609	Pereira AC, Eggertsson H, Martinez-Mier EA, Mialhe FL, Eckert GJ, Zero DT. Validity of caries detection on occlusal surfaces and treatment decisions based on results from multiple caries-detection methods. European Journal of Oral Sciences. 2009;117(1):51-7.	Excluded by title
610	Pereira IF, Santiago FZM, Sette-Dias AC, Noronha V. Taking advantage of an unerupted third molar: a case report. Dental Press J Orthod. 2017;22(4):97-101.	Excluded by title
611	Peres RCR, Scarel-Caminaga RM, Do Espírito Santo AR, Line SRP. Association between PAX-9 promoter polymorphisms and hypodontia in humans. Archives of Oral Biology. 2005;50(10):861-71.	Excluded by title
612	Perry DA, Newman MG. Occurrence of periodontitis in an urban adolescent population. Journal of periodontology. 1990;61(3):185-8.	Excluded by title
613	Pessôa CP, Pion L, Reyes A, Matos R, Alencar CJF, Novaes TF, et al. Conservative approach for molar-incisor hypomineralization: A case report and 7-year follow-up. General Dentistry. 2018;66(3):e1-e4.	Excluded by title

614	Petcu A, Maxim A, Haba D. [Correlation between the lower first permanent molar axis and the premature loss of temporary molars]. Rev Med Chir Soc Med Nat Iasi. 2009;113(4):1253-7.	Excluded by title
615	Pilmanis AA, Webb JT, Kannan N, Balldin UI. The risk of altitude decompression sickness at 12,000 m and the effect of ascent rate. Aviat Space Environ Med. 2003;74(10):1052-7.	Excluded by title
616	Pino Guerrero EF, Castillo Cevallos JL. Toma de decisión para colocar mantenedor de espacio después de la pérdida prematura de primeros molares primarios: revisión de la literatura. Rev odontopediatr latinoam. 2017;7(1):37-43.	Excluded by title
617	Piva LM, Brito HHA, Leite HR, O'Reilly M. Effects of cervical headgear and fixed appliances on the space available for maxillary second molars. American Journal of Orthodontics and Dentofacial Orthopedics. 2005;128(3):366-71.	Excluded by title
618	Plakwicz P, Czochrowska EM, Milczarek A, Zadurska M. Vertical bone growth following autotransplantation of the developing maxillary third molar to replace a retained mandibular permanent molar: a case report. Int J Periodontics Restorative Dent. 2014;34(5):667-71.	Excluded by title
619	Plotkin M, Bishanga D, Kidanto H, Jennings MC, Ricca J, Mwanamsangu A, et al. Tracking facility-based perinatal deaths in Tanzania: Results from an indicator validation assessment. PLoS One. 2018;13(7):e0201238.	Excluded by title
620	Pomarico L, Primo LG, Noce D. Ectopic eruption of the maxillary central permanent incisors and mandibular first permanent molars: Report of an unusual case. Quintessence International. 2006;37(9):677-83.	Excluded by title
621	Poveda R, Jiménez Y, Gavaldá C, Sanchís JM, Carbonell E, Margaix M, et al. Pediatric dental care in a tertiary public hospital. Four years of experience in the Service of Stomatology of Valencia University General Hospital (Valencia, Spain). Medicina Oral, Patologia Oral y Cirugia Bucal. 2008;13(5):331-5.	Excluded by title
622	Prabhakar AR, Basappa N, Raju OS. Foreign body in a mandibular permanent molar--a case report. J Indian Soc Pedod Prev Dent. 1998;16(4):120-1.	Excluded by title
623	Prado C, Garone Netto N. Pit sealing of deciduous and permanent molars. In vivo evaluation. Revista de odontologia da Universidade de São Paulo / USP. 1990;4(4):329-33.	Excluded by title
624	Prati C, Chersoni S, Mongiorgi R, Pashley DH. Resin-infiltrated dentin layer formation of new bonding systems. Operative Dentistry. 1998;23(4):185-94.	Excluded by title
625	Primozic J, Federici Canova F, Rizzo FA, Marzo G, Quinzi V. Diagnostic ability of the primary second molar crown-to-root length ratio and the corresponding underlying premolar position in estimating future expander anchoring teeth exfoliation. Orthod Craniofac Res. 2021;24(4):561-7.	Excluded by title
626	Pujol TA, Serban N, Swann J, Kottke M. Medicaid Claims for Contraception Among Women With Medical Conditions After Release of the US Medical Eligibility Criteria for Contraceptive Use. Prev Chronic Dis. 2019;16:E03.	Excluded by title
627	Puppin-Rontani RM, Baglioni-Gouvea ME, DeGoes MF, Garcia-Godoy F. Compomer as a pit and fissure sealant: Effectiveness and retention after 24 months. Journal of Dentistry for Children. 2006;73(1):31-6.	Excluded by title
628	Pytlik W. Primary failure of eruption: a case report. International dental journal. 1991;41(5):274-8.	Excluded by title
629	Qin D, Jiang HF, Shen L, Zhang C, Chai ZW, Wang JH. [Prevalence of dental caries and associated factors among 10-12-year-old students in Chongqing]. Hua Xi Kou Qiang Yi Xue Za Zhi. 2019;37(6):608-14.	Excluded by title
630	Qin M, Liu HS. Clinical evaluation of a flowable resin composite and flowable compomer for preventive resin restorations. Operative Dentistry. 2005;30(5):580-7.	Excluded by title
631	Qudeimat MA, Alomari QD, Altarakemah Y, Alshawaf N, Honkala EJ. Variables affecting the inter- and intra-examiner reliability of ICDAS for occlusal caries diagnosis in permanent molars. Journal of Public Health Dentistry. 2016;76(1):9-16.	Excluded by title
632	Qudeimat MA, Altarakemah Y, Alomari Q, Alshawaf N, Honkala E. The impact of ICDAS on occlusal caries treatment recommendations for high caries risk patients: An in vitro study. BMC Oral Health. 2019;19(1).	Excluded by title
633	Quinn GW. Extraction of 4 2nd Molars. Angle Orthodontist. 1985;55(1):58-69.	Excluded by title
634	Quinn GW. Extraction of four second molars. Angle Orthod. 1985;55(1):58-69.	Excluded by title
635	Quintella C, Damante JH, Janson G, Guerra E, de Freitas MR, Starling C. Orthodontic therapy in patients with pericoronal hamartoma. Am J Orthod Dentofacial Orthop. 2008;133(5):758-61.	Excluded by title
636	Quinzi V, Federici Canova F, Rizzo FA, Marzo G, Rosa M, Primozic J. Factors related to maxillary expander loss due to anchoring deciduous molars exfoliation during treatment in the mixed dentition phase. Eur J Orthod. 2021;43(3):332-7.	Excluded by title
637	Qureshi JV, Goldner M, Le Riche WH, Hargreaves JA. Streptococcus mutans serotypes in young schoolchildren. Caries Research. 1977;11(3):141-52.	Excluded by title
638	Rad AS, Reid J. Delayed eruption of a permanent molar associated with a complex odontoma: Report of case. Journal of Dentistry for Children. 1996;63(4):299-&.	Excluded by title
639	Raghoebar GM, Boering G, Booy K, Vissink A. Treatment of the retained permanent molar. J Oral Maxillofac Surg. 1990;48(10):1033-8.	Excluded by title
640	Raghoebar GM, Boering G, Vissink A, Stegenga B. Eruption disturbances of permanent molars: a review. Journal of Oral Pathology & Medicine. 1991;20(4):159-66.	Excluded by title
641	Rahimah SB, Djunaedi DD, Soeroto AY, Bisri T. The phytochemical screening, total phenolic contents and antioxidant activities in vitro of white oyster mushroom (Pleurotus ostreatus) preparations. Open Access Macedonian Journal of Medical Sciences. 2019;7(15):2404-12.	Excluded by title
642	Raja A, White DA, Kerr SE, Dietrich T. Prevention in the context of caries-related extractions under general anaesthesia: an evaluation of the use of sealants and other preventive care by referring dentists. Br Dent J. 2019;227(6):489-95.	Excluded by title
643	Rapeepattana S, Suntornlohanakul S, Thearmontree A. Orthodontic treatment needs of children with high caries using Index for Preventive and Interceptive Orthodontic Needs (IPION). Eur Arch Paediatr Dent. 2019;20(4):351-8.	Excluded by title
644	Rapp R, Matthews G, Simpson M, Pashley DH. In vitro permeability of furcation dentin in permanent teeth. Journal of Endodontics. 1992;18(9):444-7.	Excluded by title
645	Rasheed B, Khalid A, Wahab A, Zahid A, Ahmed A, Sarfraz T. EXAMINATION OF THE EFFECT OF PERSISTENT MOLAR LOSS ON LENGTH OF ORTHODONTIC THERAPY FOR SPACE CLOSURE. NeuroQuantology. 2023;21(5):1438-46.	Excluded by title
646	Raskin SE, Tranby EP, Ludwig S, Okunev I, Frantsve-Hawley J, Boynes S. Survival of silver diamine fluoride among patients treated in community dental clinics: a naturalistic study. BMC Oral Health. 2021;21(1).	Excluded by title
647	Rayman M. Early extraction of four first permanent molars: report of case. ASDC journal of dentistry for children. 1979;46(3):234-7.	Excluded by title
648	Rebellato J, Lindauer SJ, Rubenstein LK, Isaacson RJ, Davidovitch M, Vroom K. Lower arch perimeter preservation using the lingual arch. American journal of orthodontics and dentofacial orthopedics : official publication of the American Association of Orthodontists, its constituent societies, and the American Board of Orthodontics. 1997;112(4):449-56.	Excluded by title
649	Ren J, Wu Y, Tao X. MRI texture analysis in differential diagnosis of orbital lymphoma and inflammatory pseudotumor. Chinese Journal of Medical Imaging Technology. 2017;33(7):980-4.	Excluded by title
650	Retief DH, Mandras RS, Russell CM, Denys FR. Extracted human versus bovine teeth in laboratory studies. American journal of dentistry. 1990;3(6):253-8.	Excluded by title
651	Retief DH, Wendt SL, Bradley EL, Denys FR. The effect of storage media and duration of storage of extracted teeth on the shear bond strength of Scotchbond 2/Silux to dentin. American journal of dentistry. 1989;2(5):269-73.	Excluded by title
652	Retief DH, Wendt SL, Bradley EL. Effect of adhesive thickness on the shear bond strength of Scotchbond 2/Silux to dentin. American journal of dentistry. 1989;2(6):341-4.	Excluded by title
653	Rezaei F, Imani MM, Khavid A, Nabavi A. Patterns of mandibular third molar impaction in an Iranian subpopulation. Pesquisa Brasileira em Odontopediatria e Clinica Integrada. 2020;20:1-9.	Excluded by title
654	Richards W. What should we be doing for children in our general practices? Evidence-Based Dentistry. 2020;21(3):100-1.	Excluded by title
655	Richardson ME, Richardson A. The effect of extraction of four second permanent molars on the incisor overbite. European Journal of Orthodontics. 1993;15(4):291-6.	Excluded by title
656	Richardson ME. Late lower arch crowding: the aetiology reviewed. Dental update. 2002;29(5):234-8.	Excluded by title
657	Richardson ME. Second permanent molar extraction and late lower arch crowding: a ten-year longitudinal study. Aust Orthod J. 1996;14(3):163-7.	Excluded by title
658	Rindler A. [Second permanent molar extraction in Class II cases with crowding in the lower arch]. Sven Tandlak Tidskr. 1970;63(10):699-708.	Excluded by title
659	Rizzatto SM, de Menezes LM, do Rego MV, Thiesen G, de Araujo VP, Freitas MP. Maxillary first permanent molar impaction. A conservative treatment approach. J Clin Pediatr Dent. 2005;30(2):169-73.	Excluded by title
660	Roberts MW. Treatment of ectopically erupting maxillary permanent first molars with a distal extended stainless steel crown. ASDC J Dent Child. 1986;53(6):430-2.	Excluded by title
661	Rock WP, Gordon PH, Bradnock G. Caries Experience in West Midland School-Children Following Fluoridation of Birmingham Water in 1964 - Caries of 1st Permanent Molars. British Dental Journal. 1981;150(10):269-73.	Excluded by title
662	Rodd HD, Boissonade FM, Day PF. Pulpal status of hypomineralized permanent molars. Pediatric Dentistry. 2007;29(6):514-20.	Excluded by title
663	Rodd HD, Boissonade FM. Comparative immunohistochemical analysis of the peptidergic innervation of human primary and permanent tooth pulp. Archives of Oral Biology. 2002;47(5):375-85.	Excluded by title
664	Rodd HD, Boissonade FM. Immunocytochemical investigation of immune cells within human primary and permanent tooth pulp. International Journal of Paediatric Dentistry. 2006;16(1):2-9.	Excluded by title
665	Rodd HD, Gerrard MP. Multifocal Langerhans' cell histiocytosis in a 4-year-old child: a case report. Int J Paediatr Dent. 1994;4(1):35-40.	Excluded by title
666	Rodd HD, Morgan CR, Day PF, Boissanade FM. Pulpal expression of TRPV1 in molar incisor hypomineralisation. European archives of paediatric dentistry : official journal of the European Academy of Paediatric Dentistry. 2007;8(4):184-8.	Excluded by title
667	Rodrigues MP, Soares PBF, Gomes MAB, Pereira RA, Tantbirojn D, Versluis A, et al. Direct resin composite restoration of endodontically-treated permanent molars in adolescents: Bite force and patient-specific finite element analysis. Journal of Applied Oral Science. 2020;28:1-11.	Excluded by title
668	Rodríguez Orizondo MdF, Mursulí Sosa M, Pérez García LM, Martínez Rodríguez M. Estado de salud del primer molar permanente en niños de 6-11 años. Sancti Spíritus. 2011. Gac méd espirit. 2013;15(1):37-47.	Excluded by title
669	Rönnerman A. The effect of early loss of primary molars on tooth eruption and space conditions. A longitudinal study. Acta Odontol Scand. 1977;35(5):229-39.	Excluded by title
670	Roosevelt-Silva B, Gustavo-Chab P, Fernando-Pedrin-Carvalho F, José-Roberto-Magalhães B, Guilherme J, Raquel-Conceição F. Contextual and Individual Determinants of Anterior Open Bite in Adolescents. Int Dent J. 2023;73(1):128-35.	Excluded by title
671	Roosevelt-Silva B, Gustavo-Chab P, Fernando-Pedrin-Carvalho F, Jose-Roberto-Magalhaes B, Guilherme J, Raquel-Conceicao F. Contextual and Individual Determinants of Anterior Open Bite in Adolescents. International Dental Journal. 2023;73(1):128-35.	Excluded by title
672	Ros Santana M, Martín Gomar J, Pérez Cabrera DL. Estado clínico de primeros molares permanentes en 3 escuelas primarias del reparto Jesús Menéndez, Bayamo 2005. MULTIMED. 2006;10(2).	Excluded by title
673	Rosa FM, Stankiewicz A, Faraco IM, Jr. Impaction of mandibular molar by supernumerary tooth: case report. J Dent Child (Chic). 2008;75(2):181-4.	Excluded by title
674	Rowlison T, Ottinger MA, Comizzoli P. Progressive incorporation of cenexin is related to sperm maturation during epididymal transit in the domestic cat. Reproduction, Fertility and Development. 2015;27(1):224.	Excluded by title
675	Rubin PF, Winocur E, Erez A, Birenboim-Wilensky R, Peretz B. Dental Treatment Needs among Children and Adolescents Residing in an Ugandan Orphanage. J Clin Pediatr Dent. 2016;40(6):486-9.	Excluded by title
676	Rushing SE. Completely submerged primary molars. Miss Dent Assoc J. 1996;52(4):26-7.	Excluded by title
677	Sabeti AK, Karimizadeh Z, Rafatjou R. Maximum equivalent stress induced and the displacement of the developing permanent first molars after the premature loss of primary second molars: A finite element analysis. Dent Med Probl. 2020;57(4):401-9.	Excluded by title
678	Sağdiç D, Işimer Y, Unal U. [Application for treatment of ectopic eruption]. Turk Ortodonti Derg. 1989;2(2):378-80.	Excluded by title
679	Saito E, Watanabe S, Maeyama Y, Niwa M, Igarashi S. [Impacted bilateral first permanent molars induced by the migrated second permanent molars]. Shoni Shikagaku Zasshi. 1990;28(4):1125-30.	Excluded by title
680	Sajovic J, Meglic A, Corradi Z, Khan M, Maver A, Vidmar MJ, et al. ABCA4 Variant c.5714+5G>A in Trans With Null Alleles Results in Primary RPE Damage. Invest Ophthalmol Vis Sci. 2023;64(12):33.	Excluded by title
681	Sakkas C, Khomenko L, Trachuk I. A comparative study of clinical effectiveness of fissure sealing with and without bonding systems: 3-year results. Eur Arch Paediatr Dent. 2013;14(2):73-81.	Excluded by title

682	Sakoolnamarka R, Burrow MF, Kubo S, Tyas MJ. Morphological study of demineralized dentine after caries removal using two different methods. Australian Dental Journal. 2002;47(2):116-22.	Excluded by title
683	Salzedas LMP, Louzada MJQ, Oliveira Filho ABd. Radiopacity of restorative materials using digital images. J appl oral sci. 2006;14(2):147-52.	Excluded by title
684	Samrit V, Kharbanda OP, Duggal R, Seith A, Malhotra V. Bone density and miniscrew stability in orthodontic patients. Aust Orthod J. 2012;28(2):204-12.	Excluded by title
685	Sana S, Kondody RT, Talapaneni AK, Fatima A, Bangi SL. Occlusal stress distribution in the human skull with permanent maxillary first molar extraction: A 3-dimensional finite element study. Am J Orthod Dentofacial Orthop. 2021;160(4):552-9.	Excluded by title
686	Santin GC, Palma-Dibb RG, Romano FL, De Oliveira HF, Nelson Filho P, De Queiroz AM. Physical and adhesive properties of dental enamel after radiotherapy and bonding of metal and ceramic brackets. American Journal of Orthodontics and Dentofacial Orthopedics. 2015;148(2):283-92.	Excluded by title
687	Santos PBd. Efeitos periodontais da movimentação dentária de molares inferiores para área de rebordo alveolar atrófico. 2014. p. 137-.	Excluded by title
688	Santschi K, Peutzfeldt A, Lussi A, Flury S. Effect of salivary contamination and decontamination on bond strength of two one-step self-etching adhesives to dentin of primary and permanent teeth. Journal of Adhesive Dentistry. 2015;17(1):51-7.	Excluded by title
689	Şaroğlu Sönmez I, Akbay Oba A, Erkmen M, Ekici S. Effects of different fissure sealant applications on laser fluorescence measurements. International Journal of Paediatric Dentistry. 2011;21(1):29-34.	Excluded by title
690	Savoldi F, Dalessandri D, Gardoni A, Dianiskova S, Bonetti S, Visconti L. Treatment of ankylosed deciduous molars with or without permanent successors in children and adolescents: a systematic review. Minerva Dent Oral Sci. 2021;70(6):276-85.	Excluded by title
691	Scherrer C, Naavaal S, Lin M, Griffin SO. COVID-19 Pandemic Impact on US Childhood Caries and Potential Mitigation. J Dent Res. 2022;101(10):1147-54.	Excluded by title
692	Schmalfuss A, Stenhagen KR, Tveit AB, Crossner CG, Espelid I. Canines are affected in 16-year-olds with molar–incisor hypomineralisation (MIH): an epidemiological study based on the Tromsø study: "Fit Futures". European Archives of Paediatric Dentistry. 2016;17(2):107-13.	Excluded by title
693	Schnabl D, Fleischer F, Riedmann M, Laimer J, Gaßner R. Prevalence and distribution of deep caries and abscess formation in children who required emergency dental general anaesthesia. A retrospective analysis. European Journal of Paediatric Dentistry. 2019;20(2):119-22.	Excluded by title
694	Schröder U, Granath L. A new interceptive treatment of cases with missing maxillary lateral incisors. A preliminary report. Swed Dent J. 1981;5(4):155-8.	Excluded by title
695	Schwendicke F, Rossi JG, Krois J, Basso M, Peric T, Turkun LS, et al. Cost-effectiveness of glass hybrid versus composite in a multi-country randomized trial. Journal of Dentistry. 2021;107.	Excluded by title
696	Schwendicke F, Stolpe M, Meyer-Lueckel H, Paris S. Detecting and treating occlusal caries lesions: a cost-effectiveness analysis. J Dent Res. 2015;94(2):272-80.	Excluded by title
697	Sechet P, Boutonnet P, Bourgeois G, Borde MM. [Importance of the permanent first molar in children]. Rev Odontostomatol Midi Fr. 1975(3):142-64.	Excluded by title
698	Seddon JL. Extraction of four first molars: A case for a general practitioner? Journal of Orthodontics. 2004;31(2):80-5.	Excluded by title
699	Seehra J, Winchester L, DiBiase AT, Cobourne MT. Orthodontic management of ectopic maxillary first permanent molars: a case report. Aust Orthod J. 2011;27(1):57-62.	Excluded by title
700	Sehrawat JS, Tabasum, Talwar M. Discriminant function analysis of elemental composition of molar teeth for sex determination of Northwest Indian subjects: A forensic anthropological study. Journal of Forensic Medicine and Toxicology. 2019;36(1):93-100.	Excluded by title
701	Selikowitz HS. Prevalence of dental caries in a group of adult Vietnamese refugees in Norway. Community dentistry and oral epidemiology. 1984;12(1):51-4.	Excluded by title
702	Shah A, Sharma S, Boruah S, Mukherjee A, Tyagi G, Singh A. Comparing Expansion of Maxillary Antrum between Periapical Surgery and Extraction of Permanent Maxillary First Molar in Pediatric Patients Using CBCT. Journal of Contemporary Dental Practice. 2022;23(6):606-12.	Excluded by title
703	Shalish M, Peck S, Wasserstein A, Peck L. Increased occurrence of dental anomalies associated with infraocclusion of deciduous molars. Angle Orthod. 2010;80(3):440-5.	Excluded by title
704	Shtereva L, Kondeva V. Twelve-Month Clinical Evaluation of Retention of Resin-Based Sealant on First Permanent Molars. Folia Medica. 2023;65(4):651-8.	Excluded by title
705	Shuman I, Cardo VA, Jr. Tooth Development Following Mandibular Distraction Osteogenesis in Neonates With Pierre Robin Sequence. J Craniofac Surg. 2021;32(2):675-7.	Excluded by title
706	Sian JS. Root resorption of first permanent molar by a supernumerary premolar. Dent Update. 1999;26(5):210-1.	Excluded by title
707	Sidaly R, Schmalfuss A, Skaare AB, Sehic A, Stiris T, Espelid I. Five-minute Apgar score ≤ 5 and Molar Incisor Hypomineralisation (MIH) - a case control study. BMC Oral Health. 2016;17(1).	Excluded by title
708	Silva EHEd, Borges FM, Cruz FCSd, Pena GdG. Associação entre Estado Nutricional e Força de Preensão Manual em Pacientes Oncológicos em Cuidados Paliativos. Rev bras cancerol. 2018;64(4):479-87.	Excluded by title
709	Silva MdCS, Brito MLdR, Araujo SM, Carvalho AMO, Alves Neto AA, Pastor IMO, et al. Condições dos primeiros molares permanentes em 1202 escolares da cidade de Salvador na faixa etária de 7 a 14 anos. Rev fac odontol Univ Fed Bahia. 1984;(4):9-25.	Excluded by title
710	Simu MR, Suhani R, Mesaros M. Increasing the difficulty of orthodontic treatment by the of loss of leeway space - A case report. Clujul Medical. 2016;89:S183.	Excluded by title
711	Singh C, Kaur K, Kapoor K. Retention of pit and fissure sealant versus flowable composite: An in vivo one-year comparative evaluation. Journal of Indian Society of Pedodontics and Preventive Dentistry. 2019;37(4):372-7.	Excluded by title
712	Singh S, Pandey RK. An evaluation of nanocomposites as pit and fissure sealants in child patients. Journal of Indian Society of Pedodontics and Preventive Dentistry. 2011;29(4):294-9.	Excluded by title
713	Sivarajkumar S, Huang Y, Wang Y. Fair patient model: Mitigating bias in the patient representation learned from the electronic health records. J Biomed Inform. 2023;148:104544.	Excluded by title
714	Skjeflo A, Zachrisson BU. [Ectopic tooth eruption of the first permanent molar in the maxilla]. Nor Tannlaegeforen Tid. 1971;81(3):203-10.	Excluded by title
715	Skolnick IM. Ankylosis of maxillary permanent first molar. J Am Dent Assoc. 1980;100(4):558-60.	Excluded by title
716	Smaïl-Faugeron V, Fron-Chabouis H, Courson F. Methodological quality and implications for practice of systematic Cochrane reviews in pediatric oral health: A critical assessment. BMC Oral Health. 2014;14(1).	Excluded by title
717	Smales RJ, Fang DTS. In vitro Effectiveness of Hand Excavation of Caries with the ART Technique. Caries Research. 1999;33(6):437-40.	Excluded by title
718	Smith NK, Morris KT, Wells M, Tantblrojn D, Versluis A. Rationale for caries inhibition of debonded glass Ionomer sealants: An in vitro study. Pediatric Dentistry. 2014;36(7):464-7.	Excluded by title
719	Smith R. The effects of extracting upper second permanent molars on lower second permanent molar position. Br J Orthod. 1996;23(2):109-14.	Excluded by title
720	Soares FZM, De Oliveira Rocha R, Raggio DP, Sadek FT, Cardoso PEC. Microtensile bond strength of different adhesive systems to primary and permanent dentin. Pediatric Dentistry. 2005;27(6):457-62.	Excluded by title
721	Sobue S, Okamoto M, Moriwaki Y. Caries-preventive effects of polycarboxylate cement containing Na2PO3F sealed on occlusal surfaces in newly erupted permanent molars. Caries Research. 1981;15(4):289-95.	Excluded by title
722	Sogur E, Baksi BG, Mert A. The effect of delayed scanning of storage phosphor plates on occlusal caries detection. Dentomaxillofac Radiol. 2012;41(4):309-15.	Excluded by title
723	SolmazgÜL Yazici Z, Arslan H. Spectrophotometric Analysis of Color Stability Induced by Various Calcium Silicate Cements in Full Pulpotomy of Permanent Molars: Theracal PT, Biodentine, and ProRoot MTA. Journal of Endodontics. 2023.	Excluded by title
724	Somwanshi YI, Katre AN, Jawdekar AM. Modified distal shoe appliance for multiple loss of first and second primary molars. Journal of Clinical and Diagnostic Research. 2016;10(10):ZJ03-ZJ4.	Excluded by title
725	Soni HK. Biodentine pulpotomy in mature permanent molar: A case report. Journal of Clinical and Diagnostic Research. 2016;10(7):ZD09-ZD11.	Excluded by title
726	Soviero V, Haubek D, Trindade C, Da Matta T, Poulsen S. Prevalence and distribution of demarcated opacities and their sequelae in permanent 1st molars and incisors in 7 to 13-year-old Brazilian children. Acta Odontologica Scandinavica. 2009;67(3):170-5.	Excluded by title
727	Splieth CH, Banerjee A, Bottenberg P, Breschi L, Campus G, Ekstrand KR, et al. How to Intervene in the Caries Process in Children: A Joint ORCA and EFCD Expert Delphi Consensus Statement. Caries Research. 2020;54(4):297-305.	Excluded by title
728	Srinivasan V, Deery C, Nugent Z. In-vitro microleakage of repaired fissure sealants: a randomized, controlled trial. Int J Paediatr Dent. 2005;15(1):51-60.	Excluded by title
729	Sripetchdanond J, Leevailoj C. Wear of human enamel opposing monolithic zirconia, glass ceramic, and composite resin: an in vitro study. J Prosthet Dent. 2014;112(5):1141-50.	Excluded by title
730	Steinberg J, Buckley R, Ickow I. Design of a novel orthodontic appliance to prevent pedicle trauma in patients undergoing double-opposing buccal flaps for palatal lengthening following maxillary advancement. Cleft Palate-Craniofacial Journal. 2021;58(4 SUPPL):12.	Excluded by title
731	Stephen KW, Kirkwood M, Campbell D, Young KC, Gillespie FC, Boyle P. Fissure sealing with Nuva-seal and Alphaseal: two-year data. Journal of Dentistry. 1981;9(1):53-7.	Excluded by title
732	Stephenson PA. Dental management of fibrous dysplasia. N Z Dent J. 1993;89(395):54-8.	Excluded by title
733	Stevenson AG. An unusual complication following the extraction of a mandibular permanent molar. Oral Surg Oral Med Oral Pathol. 1980;50(4):385.	Excluded by title
734	Stivaros N, Lowe C, Dandy N, Doherty B, Mandall NA. A randomized clinical trial to compare the Goshgarian and Nance palatal arch. Eur J Orthod. 2010;32(2):171-6.	Excluded by title
735	Suggett JA, Nagel MW, Schneider H, Avvakoumova V, Ali R, Mitchell J. Evaluation of performance of valved holding chambers (VHCS) used out-of-packaging for speed of medication delivery: Careful selection of the VHC is required. American Journal of Respiratory and Critical Care Medicine. 2015;191.	Excluded by title
736	Sun W, Chen H, Zhong Y, Zhang W, Chu F, Li L, et al. Three-Dimensional Tooth Models with Pulp Cavity Enhance Dental Anatomy Education. Anat Sci Educ. 2022;15(3):566-75.	Excluded by title
737	Suni J, Vähänikkilä H, Päkkilä J, Tjäderhane L, Larmas M. Review of 36,537 patient records for tooth health and longevity of dental restorations. Caries Res. 2013;47(4):309-17.	Excluded by title
738	Sutalo J, Ciglar I, Stanicic T. Endodontic System in Root Fusion of the Second Lower Permanent Molar. Acta Stomatologica Croatica. 1987;21(2):107-14.	Excluded by title
739	Sutalo J, Simeon P, Tarle Z, Prskalo K, Pevalek J, Stanicić T, et al. "C"-shaped canal configuration of mandibular second permanent molar. Coll Antropol. 1998;22(1):179-86.	Excluded by title
740	Taboada-Aranza O, Rodríguez-Nieto K. [Prevalence of plaque and dental decay in the first permanent molar in a school population of south Mexico City]. Bol Med Hosp Infant Mex. 2018;75(2):113-8.	Excluded by title
741	Taboada-Aranza O, Rodríguez-Nieto K. Prevalencia de placa dentobacteriana y caries dental en el primer molar permanente en una población escolar del sur de la Ciudad de México. Bol méd Hosp Infant Méx. 2018;75(2):113-8.	Excluded by title
742	Tai ZW, Peng WW, Du R, Zhu YQ. Evaluation of glide path preparation and shaping ability of two nickel-titanium engine driven systems in moderate curved root canals. Journal of Shanghai Jiaotong University (Medical Science). 2020;40(1):89-92.	Excluded by title
743	Tajmehr N, Graham A, Deery C. Should we root treat children's first permanent molars? Evid Based Dent. 2020;21(4):142-3.	Excluded by title
744	Talasaz AH, Powell AA, Huber DE, Berbee JG, Roh KH, Yu W, et al. Isolating highly enriched populations of circulating epithelial cells and other rare cells from blood using a magnetic sweeper device. Proc Natl Acad Sci U S A. 2009;106(10):3970-5.	Excluded by title
745	Tan KT, Zhan JQ, Chiu S, Pasian SG, Goyal K, Leung G, et al. MRI molecular imaging of VCAM-1. Journal of Vascular and Interventional Radiology. 2010;21(2):S127.	Excluded by title
746	Tan SP, Kuitert RB, Prahl-Andersen B. [Ectopic eruption of the maxillary first permanent molar]. Ned Tijdschr Tandheelkd. 2004;111(8):307-10.	Excluded by title
747	Tang R, Hu Y, Lin Z. Classification of furcation involvement of endodontically treated mandibular first permanent molars based on cone beam CT. Journal of Prevention and Treatment for Stomatological Diseases. 2023;31(7):488-93.	Excluded by title
748	Tarım Ertas E, Küçükyılmaz E, Ertaş H, Savaş S, Yırcalı Atıcı M. A comparative study of different radiographic methods for detecting occlusal caries lesions. Caries Res. 2014;48(6):566-74.	Excluded by title
749	Taşsöker M, Azman D, Aydin Kabakçi AD. Morphometric Analysis of the Foramen Palatinum Majus by Cone-beam Computed Tomography. Bezmialem Science. 2023;11(1):1-7.	Excluded by title
750	Tassoker M, Ozcan S, Karabekiroglu S. Occlusal Caries Detection and Diagnosis Using Visual ICDAS Criteria, Laser Fluorescence Measurements, and Near-Infrared Light Transillumination Images. Med Princ Pract. 2020;29(1):25-31.	Excluded by title
751	Tatoń G, Ziomber A, Rokita E, Ciesielczyk K, Thor P. Adipose tissue quantification in rats with the use of computed tomography. Current Medical Imaging Reviews. 2018;14(1):53-8.	Excluded by title
752	Taylor GD, Pearce KF, Vernazza CR. Management of compromised first permanent molars in children: Cross-Sectional analysis of attitudes of UK general dental practitioners and specialists in paediatric dentistry. International Journal of Paediatric Dentistry. 2019;29(3):267-80.	Excluded by title
753	Taylor NG, Gravely JF, Hume WJ. Resorption of the crown of an unerupted permanent molar. Int J Paediatr Dent. 1991;1(2):89-92.	Excluded by title
754	Taylor PJ, Kerr WJ, McColl JH. Factors associated with the standard and duration of orthodontic treatment. Br J Orthod. 1996;23(4):335-41.	Excluded by title
755	Taylor RF. Controlled serial extraction. American Journal of Orthodontics. 1971;60(6):576-99.	Excluded by title

756	Tekbas Atay M, Koray F. Microbiological and SEM assessment of atraumatic restorative treatment in adult dentition. Clin Oral Investig. 2021;25(12):6871-80.	Excluded by title
757	Terashima Y. A Rare Case of the Mandibular 1st Permanent Molar with 4 Roots. Aichi-Gakuin Journal of Dental Science. 1982;20(1):59-62.	Excluded by title
758	Thakkar PJ, Badakar CM, Hugar SM, Hallikerimath S, Patel PM, Shah P. An in vitro comparison of casein phosphopeptide-amorphous calcium phosphate paste, casein phosphopeptide-amorphous calcium phosphate paste with fluoride and casein phosphopeptide-amorphous calcium phosphate varnish on the inhibition of demineralization and promotion of remineralization of enamel. J Indian Soc Pedod Prev Dent. 2017;35(4):312-8.	Excluded by title
759	Thale Z, Kinder FR, Bair KW, Bontempo J, Czuchta AM, Versace RW, et al. Bengamides revisited: new structures and antitumor studies. J Org Chem. 2001;66(5):1733-41.	Excluded by title
760	Thomas P, Sandy JR. Should second molars be extracted? Dent Update. 1995;22(4):150-6.	Excluded by title
761	Thomas RM, Kelly A, Tagiyeva N, Kanagasingam S. Comparing endocrown restorations on permanent molars and premolars: a systematic review and meta-analysis. British Dental Journal. 2020.	Excluded by title
762	Tianviwat S, Chongsuvivatwong V, Sirisakulveroj B. Loss of sealant retention and subsequent caries development. Community Dental Health. 2008;25(4):216-20.	Excluded by title
763	Tickle M, Milsom K, Qualtrough A, Blinkhorn F, Aggarwal VR. The failure rate of NHS funded molar endodontic treatment delivered in general dental practice. Br Dent J. 2008;204(5):E8; discussion 254-5.	Excluded by title
764	Toedtling V, Devlin H, O'Malley L, Tickle M. A systematic review of second molar distal surface caries incidence in the context of third molar absence and emergence. British Dental Journal. 2020;228(4):261-6.	Excluded by title
765	Tollendal ME, Leite ICG. Indice de mortalidade do 1§ molar permanente. Rev odontopediatr. 1993;2(4):195-201.	Excluded by title
766	Tong HJ, Tahmassebi JF. Management of a child with severe hypodontia in the mixed dentition stage of development. European Archives of Paediatric Dentistry. 2014;15(6):449-54.	Excluded by title
767	Topaloglu-Ak A, Onçağ O, Gökçe B, Bent B. The effect of different enamel surface treatments on microleakage of fissure sealants. Acta Med Acad. 2013;42(2):223-8.	Excluded by title
768	Trentesaux T, Rousset MM, Dehaynin E, Laumaillé M, Delfosse C. 15-year follow-up of a case of amelogenesis imperfecta: Importance of psychological aspect and impact on quality of life. European Archives of Paediatric Dentistry. 2013;14(1):47-51.	Excluded by title
769	Triana R, Prado C, Garro J, García-Godoy F. Dentin bond strength of fluoride-releasing materials. American journal of dentistry. 1994;7(5):252-4.	Excluded by title
770	Tripodi D, Filippakos A, Piattelli A, D'Ercole S, Perrotti V. Wear of dental sealing materials using the replication technique. European Journal of Paediatric Dentistry. 2011;12(2):95-8.	Excluded by title
771	Tschechne S, Müller B, Dibbets J. Sagittal space relations in the maxilla during molar eruption. J Orofac Orthop. 2008;69(2):94-8.	Excluded by title
772	Tulunoglu O, Tulunoglu I, Ulusu T, Genç Y. Penetration of radiocalcium at the margins of resin and glass ionomer dentine bonding agents in primary and permanent teeth. J Dent. 2000;28(7):481-6.	Excluded by title
773	Tuo D, Zhang Z, Wang Y, Chen G. A novel ferroptosis phenotype related clinicalmolecular prognostic signature for hepatocellular carcinoma. Hepatology. 2020;72(1 SUPPL):627A-8A.	Excluded by title
774	Turgut MD, Attar N, Onen A. Radiopacity of direct esthetic restorative materials. Operative Dentistry. 2003;28(5):508-14.	Excluded by title
775	Tutino F, Alovisi M, Bernardi M, Carpegna G, Comba A, Pasqualini D, et al. Micro-CT evaluation of ProTaper Next and WaveOne Gold shaping in maxillary first molars curved canals: An in vitro study. Giornale Italiano di Endodonzia. 2019;33(1):65-9.	Excluded by title
776	Uhlen MM, Valen H, Karlsen LS, Skaare AB, Bletsa A, Ansteinsson V, et al. Treatment decisions regarding caries and dental developmental defects in children - a questionnaire-based study among Norwegian dentists. BMC Oral Health. 2019;19(1):80.	Excluded by title
777	Uprichard KK, Potter BJ, Russell CM, Schafer TE, Adair S, Weller RN. Comparison of direct digital and conventional radiography for the detection of proximal surface caries in the mixed dentition. Pediatr Dent. 2000;22(1):9-15.	Excluded by title
778	Vadiakas GP, Roberts MW. Primary posterior crossbite: diagnosis and treatment. The Journal of clinical pediatric dentistry. 1991;16(1):1-4.	Excluded by title
779	Vaidyanathan M, Sheehy EC, Gilbert SC, Beighton D. Antimicrobial properties of dentine bonding agents determined using in vitro and ex vivo methods. J Dent. 2009;37(7):514-21.	Excluded by title
780	Vaikuntam J, Tatum NB, McGuff HS. Regional odontodysplasia: review of the literature and report of a case. J Clin Pediatr Dent. 1996;21(1):35-40.	Excluded by title
781	Vaishnavi Devi B, Ravindran V, Delphine Priscilla Antony S. Common irrigants used by pediatric dentists for permanent molar root canal therapy. International Journal of Research in Pharmaceutical Sciences. 2020;11(Special Issue 3):163-71.	Excluded by title
782	Valencia R, Espinosa R, Borovoy N, Pérez S, Ceja I, Saadia M. Deproteinization effectiveness on occlusal enamel surfaces and resultant acid etching patterns: An in vitro Study. Journal of Clinical Pediatric Dentistry. 2018;42(6):434-41.	Excluded by title
783	Valencia R, Saadia M, Grinberg G. Controlled slicing in the management of congenitally missing second premolars. Am J Orthod Dentofacial Orthop. 2004;125(5):537-43.	Excluded by title
784	Van den Bossche LH, Demeulemeester JD, Bossuyt MH. Periodontal infection leading to periostitis ossificans ("Garrè's osteomyelitis") of the mandible. Report of a case. J Periodontol. 1993;64(1):60-2.	Excluded by title
785	van der Schoot EA, Kuitert RB, van Ginkel FC, Prahl-Andersen B. Clinical relevance of third permanent molars in relation to crowding after orthodontic treatment. J Dent. 1997;25(2):167-9.	Excluded by title
786	Vanderas AP, Gizani S, Papagiannoulis L. Progression of proximal caries in children with different caries indices: a 4-year radiographic study. European archives of paediatric dentistry : official journal of the European Academy of Paediatric Dentistry. 2006;7(3):148-52.	Excluded by title
787	Vanderas AP, Kavvadia K, Papagiannoulis L. Development of caries in permanent first molars adjacent to primary second molars with interproximal caries: four-year prospective radiographic study. Pediatr Dent. 2004;26(4):362-8.	Excluded by title
788	Vanderas AP, Manetas C, Koulatzidou M, Papagiannoulis L. Progression of proximal caries in the mixed dentition: A 4-year prospective study. Pediatric Dentistry. 2003;25(3):229-34.	Excluded by title
789	Vázquez-Domínguez I, Li CHZ, Fadaie Z, Haer-Wigman L, Cremers FPM, Garanto A, et al. Identification of a Complex Allele in IMPG2 as a Cause of Adult-Onset Vitelliform Macular Dystrophy. Invest Ophthalmol Vis Sci. 2022;63(5):27.	Excluded by title
790	Ventura Portillo KA, Romero Morales KD, Merino Benítez EA. Mortalidad del primer molar permanente y principal factores asociados en usuarios del sistema público de salud en tres municipios de El Salvador, 2013. 2014. p. 41-.	Excluded by title
791	Verdonschot EH, Wenzel A, Truin GJ, König KG. Performance of electrical resistance measurements adjunct to visual inspection in the early diagnosis of occlusal caries. J Dent. 1993;21(6):332-7.	Excluded by title
792	Verma S, Dasukil S, Namdev Sable M, Routray S. Radicular variant of dens in dente (RDinD) in a patient undergoing radioisotope therapy. Journal of Taibah University Medical Sciences. 2022;17(6):1094-8.	Excluded by title
793	Vishwanath AE, Sharmada BK, Pai SS, Nandini N, Roopa T. Tooth separation potential problems. Int J Orthod Milwaukee. 2014;25(1):35-7.	Excluded by title
794	Volchansky A, Evans B, Cleaton-Jones P. Position and orientation of the maxillary first permanent molar and localized attachment loss. World J Orthod. 2004;5(2):141-6.	Excluded by title
795	Vrbič V. Retention of a fluoride-containing sealant on primary and permanent teeth 3 years after placement. Quintessence International. 1999;30(12):825-8.	Excluded by title
796	Wadenya R, Mante FK. An in vitro comparison of marginal microleakage of alternative restorative treatment and conventional glass lonomer restorations in extracted permanent molars. Pediatric Dentistry. 2007;29(4):303-7.	Excluded by title
797	Waggoner WF, Johnston WM, Schumann S, Schikowski E. Microabrasion of human enamel in vitro using hydrochloric acid and pumice. Pediatric dentistry. 1989;11(4):319-23.	Excluded by title
798	Wang Q, Yu G, Zhou XD, Peters OA, Zheng QH, Huang DM. Evaluation of X-ray projection angulation for successful radix entomolaris diagnosis in mandibular first molars in vitro. Journal of Endodontics. 2011;37(8):1063-8.	Excluded by title
799	Warnakulasuriya S. Caries susceptibility of first permanent molars and treatment needs in Sri Lankan children, aged 13-16 years, in 1986. Community Dent Health. 1991;8(2):167-72.	Excluded by title
800	Wasti F, Shearer AC, Wilson NH. Root canal systems of the mandibular and maxillary first permanent molar teeth of south Asian Pakistanis. Int Endod J. 2001;34(4):263-6.	Excluded by title
801	Waterhouse PJ, Nunn JH. Crown and root resorption of a maxillary permanent first molar by an impacted second premolar: a case report. Int J Paediatr Dent. 1995;5(4):259-62.	Excluded by title
802	Weber FN. Prophylactic orthodontics. American Journal of Orthodontics. 1949;35(8):611-35.	Excluded by title
803	Wendell JJ, Vann WF, Jr. Wear of composite resin restorations in primary versus permanent molar teeth. J Dent Res. 1988;67(1):71-4.	Excluded by title
804	Wendt LK, Koch G, Birkhed D. Long-term evaluation of a fissure sealing programme in Public Dental Service clinics in Sweden. Swedish Dental Journal. 2001;25(2):61-5.	Excluded by title
805	Wenzel A, Fejerskov O, Kidd E, Joyston-Bechal S, Groeneveld A. Depth of occlusal caries assessed clinically, by conventional film radiographs, and by digitized, processed radiographs. Caries Research. 1990;24(5):327-33.	Excluded by title
806	Wenzel A, Larsen K, Fejerskov O. Detection of occlusal caries without cavitation by visual inspection, film radiographs, xeroradiographs, and digitized radiographs. Caries Research. 1991;25(5):365-71.	Excluded by title
807	Wessels KE, Cheyne VD. A determination of the number of surfaces involved in carious extracted teeth. Journal of Dental Research. 1947;26(5):375-81.	Excluded by title
808	Whittle KW, Whittle JG. Dental caries in 12-year-old children and the effectiveness of dental services in Salford, UK in 1960, 1988 and 1997. Br Dent J. 1998;184(8):394-6.	Excluded by title
809	Whittle KW, Whittle JG. Dental caries in 1-2-year-old children and the effectiveness of dental services in Salford, UK in 1960, 1988 and 1997. British Dental Journal. 1998;184(8):394-6.	Excluded by title
810	Wilmes B, Vasudavan S, Drescher D. Maxillary molar mesialization with the use of palatal mini-implants for direct anchorage in an adolescent patient. American Journal of Orthodontics and Dentofacial Orthopedics. 2019;155(5):725-32.	Excluded by title
811	Wogelius P, Haubek D, Nechifor A, Nørgaard M, Tvedebrink T, Poulsen S. Association between use of asthma drugs and prevalence of demarcated opacities in permanent first molars in 6-to-8-year-old Danish children. Community Dent Oral Epidemiol. 2010;38(2):145-51.	Excluded by title
812	Wogelius P, Haubek D, Poulsen S. Prevalence and distribution of demarcated opacities in permanent 1st molars and incisors in 6 to 8-year-old Danish children. Acta Odontologica Scandinavica. 2008;66(1):58-64.	Excluded by title
813	Wohltmann W, Matz R. Cutaneous sinus of dental origin. Journal of the American Academy of Dermatology. 2019;81(4):AB189.	Excluded by title
814	Woo T, Nam K, Heo S, Lim JY, Kim S, Yoo C. Predictive maintenance system for membrane replacement time detection using AI-based functional profile monitoring: Application to a full-scale MBR plant. Journal of Membrane Science. 2022;649.	Excluded by title
815	Wright JT, Tampi MP, Graham L, Estrich C, Crall JJ, Fontana M, et al. Sealants for preventing and arresting pit-and-fissure occlusal caries in primary and permanent molars: A systematic review of randomized controlled trials—a report of the American Dental Association and the American Academy of Pediatric Dentistry. Journal of the American Dental Association. 2016;147(8):631-45.e18.	Excluded by title
816	Xu Y, Jia YH, Chen L, Huang WM, Yang DQ. Metagenomic analysis of oral microbiome in young children aged 6-8 years living in a rural isolated Chinese province. Oral Dis. 2018;24(6):1115-25.	Excluded by title
817	Yamanel K, Caglar A, Gülsahi K, Ozden UA. Effects of different ceramic and composite materials on stress distribution in inlay and onlay cavities: 3-D finite element analysis. Dent Mater J. 2009;28(6):661-70.	Excluded by title
818	Yang X, He L, Yan S, Chen X, Que G. The impact of caries status on supragingival plaque and salivary microbiome in children with mixed dentition: a cross-sectional survey. BMC Oral Health. 2021;21(1):319.	Excluded by title
819	Yanikoglu F, Avci H, Celik ZC, Tagtekin D. Diagnostic Performance of ICDAS II, FluoreCam and Ultrasound for Flat Surface Caries with Different Depths. Ultrasound Med Biol. 2020;46(7):1755-60.	Excluded by title
820	Yansane A, Listl S, Dawda D, Brandon R, White J, Spallek H, et al. Increasing value, reducing waste: tailoring the application of dental sealants according to individual caries risk. Journal of Public Health Dentistry. 2020;80(S2):S8-S16.	Excluded by title

821	Yip KHK, Smales RJ, Gao W, Peng D. The effects of two cavity preparation methods on the longevity of glass ionomer cement restorations: An evaluation after 12 months. Journal of the American Dental Association. 2002;133(6):744-51.	Excluded by title
822	Yu M, Ju S, Zhang J, Li S, Lei J, Li X. Patch-DFD: Patch-based end-to-end DeepFake discriminator. Neurocomputing. 2022;501:583-95.	Excluded by title
823	Yu YX, Hu CH, Wang XM, Fan YF, Hu MJ, Shi C, et al. [Value of the application of enhanced CT radiomics and machine learning in preoperative prediction of microvascular invasion in hepatocellular carcinoma]. Zhonghua Yi Xue Za Zhi. 2021;101(17):1239-45.	Excluded by title
824	Yuan K, Hsu PC, Tseng CC, Kiang D, Wang JR. Detection rate of Actinobacillus actinomycetemcomitans on the permanent 1st molars of primary school children in Taiwan by polymerase chain reaction. Journal of Clinical Periodontology. 2001;28(4):348-52.	Excluded by title
825	Yuen S, Chan J, Tay F. Ectopic eruption of the maxillary permanent first molar: the effect of increased mesial angulation on arch length. J Am Dent Assoc. 1985;111(3):447-51.	Excluded by title
826	Zajicek P, Radinger J, Wolter C. Disentangling multiple pressures on fish assemblages in large rivers. Sci Total Environ. 2018;627:1093-105.	Excluded by title
827	Zanolli C. Additional Evidence for Morpho-Dimensional Tooth Crown Variation in a New Indonesian H-erectus Sample from the Sangiran Dome (Central Java). Plos One. 2013;8(7).	Excluded by title
828	Zee KY, Chiu MLB, Holmgren CJ, Walker RT, Corbet EF. Cervical enamel projections in Chinese first permanent molars. Australian Dental Journal. 1991;36(5):356-60.	Excluded by title
829	Zenkner JEA, Dalla Nora A, Alves LS, Carvalho J, Wagner MB, Maltz M. Long-term follow-up of inactive occlusal caries lesions: 4-5-year results. Clin Oral Investig. 2019;23(2):847-53.	Excluded by title
830	Zhang B, Wang Y, Ding C, Deng Z, Li L, Qin Z, et al. Multi-scale feature pyramid fusion network for medical image segmentation. Int J Comput Assist Radiol Surg. 2023;18(2):353-65.	Excluded by title
831	Zhang S, Xia B, Ge LH. [Experimental study of compomer sealant with non-rinse conditioner used on permanent molar]. Hua Xi Kou Qiang Yi Xue Za Zhi. 2007;25(6):561-3.	Excluded by title
832	Zhong X, Tang R, Li J, Lu B, Yang P, Chen Z, et al. MRI texture analysis in differential diagnosis of small hepatocellular carcinoma and dysplastic nodules in cirrhosis liver. Chinese Journal of Medical Imaging Technology. 2018;34(7):1041-5.	Excluded by title
833	Zhong X-M, Jia C-M, Duan Y-F, Duan Y, Qiao X. [An epidemiological investigation of caries status of the first permanent molars of children aged 7-9 year in Taizhou City]. Shanghai kou qiang yi xue = Shanghai journal of stomatology. 2017;26(3):328-30.	Excluded by title
834	Zhou Y, Ma X, Wang D, Qin W, Zhu J, Zhuo C, et al. The selective impairment of resting-state functional connectivity of the lateral subregion of the frontal pole in schizophrenia. PLoS One. 2015;10(3):e0119176.	Excluded by title
835	Zhu F, Chen Y, Yu Y, Xie Y, Zhu H, Wang H. Caries prevalence of the first permanent molars in 6-8 years old children. PLoS One. 2021;16(1):e0245345.	Excluded by title
836	Zouaidi K, Chala S, Ameziane R, Chhoul H. [First permanent molar caries: a case study of Moroccan children between 6 and 15 year-old]. Odontostomatol Trop. 2012;35(140):5-10.	Excluded by title
837	Albadri S, Zaitoun H, McDonnell ST, Davidson LE. Extraction of first permanent molar teeth: results from three dental hospitals. Br Dent J. 2007;203(7):E14; discussion 408-9.	Excluded by abstract
838	Alkhalaf R, Neves AA, Banerjee A, Hosey MT. Minimally invasive judgement calls: managing compromised first permanent molars in children. British Dental Journal. 2020;229(7):459-65.	Excluded by abstract
839	AlKhalaf R, Neves AA, Warburton F, Banerjee A, Hosey MT. Management of compromised first permanent molars in a cohort of UK paediatric patients referred to hospital-based services. Int J Paediatr Dent. 2022;32(5):724-36.	Excluded by abstract
840	Alswairki HJ, Alam MK. Orthodontic management of compromised first permanent molars with bilateral posterior crossbite, severe crowding and impacted maxillary canines. Bangladesh Journal of Medical Science. 2021;20(4):926-9.	Excluded by abstract
841	Antelo Vázquez L, Vázquez Amoroso LM, León Pujalte Y. La pérdida del primer molar permanente en niños de 12 a 14 años de edad. Mediciego. 2012;18(supl. 2).	Excluded by abstract
842	Arunachalam S, Sivakumar I, Jayaraman J, Sharan J. Does arch length preservation in mixed dentition children affect mandibular second permanent molar eruption? A systematic review and meta-analysis. BMC Oral Health. 2021;21(1):395.	Excluded by abstract
843	Ashley P, Noar J. Interceptive extractions for first permanent molars: a clinical protocol. Br Dent J. 2019;227(3):192-5.	Excluded by abstract
844	Bandeira Lopes L, Machado V, Botelho J, Haubek D. Molar-incisor hypomineralization: an umbrella review. Acta Odontologica Scandinavica. 2021;79(5):359-69.	Excluded by abstract
845	Bassani L, Pompeo DD, Haje OE, Cericato GO, Paranhos LR. Extração de primeiros molares permanentes em tratamentos ortodônticos - relato de caso. Ortho Sci, Orthod sci pract. 2015;8(29):104-11.	Excluded by abstract
846	Bayram M, Özer M, Arici S. Effects of first molar extraction on third molar angulation and eruption space. Oral Surgery, Oral Medicine, Oral Pathology, Oral Radiology and Endodontology. 2009;107(2):e14-e20.	Excluded by abstract
847	Botelho K, Carvalho L, Maciel R, Franca Cd, Colares V. Condição clínica dos primeiros molares permanentes: de crianças entre 6 e 8 anos de idade. Odontol clín-cient. 2011;10(2):167-71.	Excluded by abstract
848	Budhdeo R, Graham A, Harrison M. Mirror-Image Large Complex Odontomes. British Journal of Surgery. 2023;110:vii69.	Excluded by abstract
849	Carmenate Elizalde A, Díaz Gutiérrez CD, Yero Mier IM, Pérez Madrigal MV. Estado de salud del primer molar permanente en 8, 10, 12 años: Área Centro. Gac méd espirit. 2008;10(2).	Excluded by abstract
850	Cernei ER, Mavru RB, Zetui IN. SECOND MOLAR UPRIGHTING AFTER PREMATURE LOSS OF MANDIBULAR FIRST PERMANENT MOLAR--CASE REPORT. Rev Med Chir Soc Med Nat Iasi. 2015;119(2):572-8.	Excluded by abstract
851	Cobourne MT, Williams A, Harrison M. National clinical guidelines for the extraction of first permanent molars in children. Br Dent J. 2014;217(11):643-8.	Excluded by abstract
852	Colangelo G. [An orthodontic problem: the obligatory extraction of a first permanent molar]. Mondo Ortod. 1976;18(5):8-18.	Excluded by abstract
853	Conway M, Petrucci D. Three cases of first permanent molar extractions where extraction of the adjacent second deciduous molar is also indicated. Dent Update. 2005;32(6):338-40, 42.	Excluded by abstract
854	Crabb JJ, Rock WP. Treatment planning in relation to the first permanent molar. Br Dent J. 1971;131(9):396-401.	Excluded by abstract
855	Cuyac Lantigua M, Reyes Martín B, Mirabal Peon M, Villegas Rojas IM, Alfonso Biart B, Duque Reyes MV. Pérdida prematura del primer molar permanente en niños de la escuela primaria "Antonio López". México. 2016. Rev medica electron. 2017;39(3):607-14.	Excluded by abstract
856	da Costa-Silva CM, Jeremias F, de Souza JF, Cordeiro RC, Santos-Pinto L, Zuanon AC. Molar incisor hypomineralization: prevalence, severity and clinical consequences in Brazilian children. International journal of paediatric dentistry / the British Paedodontic Society [and] the International Association of Dentistry for Children. 2010;20(6):426-34.	Excluded by abstract
857	de Melo FGC, Cavalcanti AL, Fontes LBC, Granville-Garcia AF, Cavalcanti SDLB. Early loss of permanent molars and associated factors in schoolchildren aged 9, 12 and 15 years attending public schools in Campina Grande, Paraíba State, Brazil. Acta Scientiarum - Health Sciences. 2011;33(1):99-105.	Excluded by abstract
858	De Sousa AS, Araújo FRL, Villela GSC, Normando D. Impact of early loss of lower first permanent molars on third molar development and position. Pesquisa Brasileira em Odontopediatria e Clinica Integrada. 2021;21.	Excluded by abstract
859	Demir P, Aydoğdu H, Sar IŞ. Knowledge, Attitude and Clinical Practice of Dentists about Extraction of First Permanent Molars. J Clin Pediatr Dent. 2022;46(1):12-9.	Excluded by abstract
860	Dopico MP, Castro C. Importancia del primer molar permanente y consecuencias clínicas de su pérdida en edades tempranas del desarrollo. Rev Ateneo Argent Odontol. 2015;54(2):23-7.	Excluded by abstract
861	Eichenberger M, Erb J, Zwahlen M, Schätzle M. The timing of extraction of non-restorable first permanent molars: a systematic review. Eur J Paediatr Dent. 2015;16(4):272-8.	Excluded by abstract
862	Evans R. Incidence of lower second permanent molar impaction. Br J Orthod. 1988;15(3):199-203.	Excluded by abstract
863	Fitzpatrick L, O'Connell A. First permanent molars with molar incisor hypomineralisation. J Ir Dent Assoc. 2007;53(1):32-7.	Excluded by abstract
864	Gill DS, Lee RT, Tredwin CJ. Treatment planning for the loss of first permanent molars. Dental update. 2001;28(6):304-8.	Excluded by abstract
865	Gómez Porcegué Y, Sánchez Rodríguez L, Martínez Nazario M, Díaz Vázquez E. Pérdida del primer molar permanente en niños de 8, 10 y 12 años. Área Sur. 2013. Gac méd espirit. 2015;17(3).	Excluded by abstract
866	González J, Manrrique R, Carballo A, Carbonell M, Córdova L, Coronel G, et al. Proyecto ANACO-UCV: Estudio epidemiológico sobre la pérdida prematura del primer molar permanente en niños con edades comprendidas entre 6 y 10 años. Acta odontol venez. 2001;39(2):42-6.	Excluded by abstract
867	Grivu O, Bratu E, Halawi M, Grivu M. [Orthodontics problems of the first permanent molar tooth]. Stomatologie. 1990;37(1):67-79.	Excluded by abstract
868	Gürcan AT, Bayram M. Children's dental treatment requirements of first permanent molars with poor prognosis. Clin Oral Investig. 2022;26(1):803-12.	Excluded by abstract
869	Haque S, Sandler J, Cobourne MT, Bassett P, DiBiase AT. A retrospective study comparing the loss of anchorage following the extraction of maxillary first or second premolars during orthodontic treatment with fixed appliances in adolescent patients. J Orthod. 2017;44(4):268-76.	Excluded by abstract
870	Hatami A, Dreyer C. The extraction of first, second or third permanent molar teeth and its effect on the dentofacial complex. Aust Dent J. 2019;64(4):302-11.	Excluded by abstract
871	Hernandez M. [Damaged first permanent molar: how conservative can we be?]. Orthod Fr. 2023;94(3-4):453-60.	Excluded by abstract
872	Hernández Pozo ME, Llano Montanet M, Díaz Narváez VP. [Malocclusion and its relation to early loss of temporary molars and first permanent molar]. Rev Cubana Estomatol. 1986;23(1):22-32.	Excluded by abstract
873	Hunter ML, Addy M, Dummer PM, Hunter B, Kingdon A, Shaw WC. A longitudinal study of the condition of first permanent molars in a group of adolescents with special reference to elective orthodontic tooth extraction. Community Dent Health. 1991;8(1):9-15.	Excluded by abstract
874	Issáo M, Pinto AC. [The first permanent molar]. Ars Curandi Odontol. 1974;1(1):20-2.	Excluded by abstract
875	Johnsen DC. A review of orthodontic sequelae to early first permanent molar extraction. Some promise--many pitfalls. W V Dent J. 1976;50(2):9-12.	Excluded by abstract
876	Kaczmarek U, Jaworski A. Molar-incisor hypomineralisation - Etiology, prevalence, clinical picture and treatment - Review. Dental and Medical Problems. 2014;51(2):165-71.	Excluded by abstract
877	Karthikeson PS, Ravindran V, Pandurangan KK. Reasons for extraction of permanent first molars in children between 6-17 years of age - A retrospective study. European Journal of Molecular and Clinical Medicine. 2020;7(1):3282-94.	Excluded by abstract

878	Kellerhoff NM, Lussi A. ["Molar-incisor hypomineralization"]. Schweiz Monatsschr Zahnmed. 2004;114(3):243-53.	Excluded by abstract
879	Khoramrooz M, Mirrezaie SM, Emamian MH, Golbabaei Pasandi H, Dadgari A, Hashemi H, et al. Economic inequalities in decayed, missing, and filled first permanent molars among 8-12 years old Iranian schoolchildren. BMC Oral Health. 2023;23(1):728.	Excluded by abstract
880	Kılınç G, Çetin M, Köse B, Ellidokuz H. Prevalence, aetiology, and treatment of molar incisor hypomineralization in children living in Izmir City (Turkey). Int J Paediatr Dent. 2019;29(6):775-82.	Excluded by abstract
881	Kim MJ, Song JS, Kim YJ, Kim JW, Jang KT, Hyun HK. Clinical considerations for dental management of children with molar-root incisor malformations. Journal of Clinical Pediatric Dentistry. 2020;44(1):55-9.	Excluded by abstract
882	Kobylińska A, Piekoszewska-Ziętek P, Gozdowski D, Turska-Szybka A, Olczak-Kowalczyk D. Spatial changes in the dental arch after premature extraction of the first primary molar – a 12-month observational study. New Medicine. 2019;23(3):96-104.	Excluded by abstract
883	Korte A, Angelopoulou MV, Yfanti K. Guidance for Permanent First Molar Extraction in Molar-Incisor Malformation: Report of Two Cases. J Dent Child (Chic). 2022;89(1):29-35.	Excluded by abstract
884	Laverde-Giraldo M, Mejía-Roldán JD, Jhonson-Giraldo N, Santos-Pinto L, Restrepo M. Consideraciones diagnósticas para la exodoncia de primeros molares permanentes severamente afectados por la Hipomineralización de Molares e Incisivos. CES odontol. 2021;34(2):210-32.	Excluded by abstract
885	Lee HS, Kim SH, Kim SO, Lee JH, Choi HJ, Jung HS, et al. A new type of dental anomaly: Molar-incisor malformation (MIM). Oral Surgery, Oral Medicine, Oral Pathology and Oral Radiology. 2014;118(1):101-9.e3.	Excluded by abstract
886	Mathu-Muju KR, Kennedy DB. Loss of Permanent First Molars in the Mixed Dentition: Circumstances Resulting in Extraction and Requiring Orthodontic Management. Pediatr Dent. 2016;38(5):46-53.	Excluded by abstract
887	Mordecai R. Treatment planning for the loss of first permanent molars. Dental update. 2002;29(2):98; author reply -9.	Excluded by abstract
888	Nicodemo RA, Miranda P, Rangel FJdC. [Frequency of loss of Japanese first permanent molar teeth]. Rev Fac Odontol Sao Jose Dos Campos. 1974;3(1):73-7.	Excluded by abstract
889	Nicodemo RA, Miranda P. [Frequency of loss of the permanent first molar teeth among Brazilians. II. Comparative study of the prevalence of the loss among individuals belonging to different economic levels of the population]. Rev Fac Odontol Sao Jose Dos Campos. 1973;2(2):97-101.	Excluded by abstract
890	Normando ADC, Silva MdC, Le Bihan R, Simone JL. Alterações oclusais espontâneas decorrentes da perda dos primeiros molares permanentes inferiores. Rev dent press ortodon ortop maxilar. 2003;8(3):15-23.	Excluded by abstract
891	Normando ADC. Análise radiográfica das alterações dento-alveolares decorrentes da perda unilateral do primeiro molar permanente inferior. 2003. p. 51-.	Excluded by abstract
892	Normando D, Cavacami C. A influência da perda bilateral do primeiro molar inferior permanente na morfologia dentofacial: um estudo cefalométrico. Dental press j orthod (Impr). 2010;15(6):100-6.	Excluded by abstract
893	Oliver RG, Oliver SJ, Dummer PM, Hicks R, Kingdon A, Addy M, et al. Loss of the first permanent molar and caries experience of adjacent teeth. Community Dent Health. 1992;9(3):225-33.	Excluded by abstract
894	Ong DC, Bleakley JE. Compromised first permanent molars: an orthodontic perspective. Aust Dent J. 2010;55(1):2-14; quiz 105.	Excluded by abstract
895	Park K, Jung DW, Kim JY. Three-dimensional space changes after premature loss of a maxillary primary first molar. International Journal of Paediatric Dentistry. 2009;19(6):383-9.	Excluded by abstract
896	Pfyffer A. [Late results of extraction of the permanent first molar]. SSO Schweiz Monatsschr Zahnheilkd. 1951;61(6):565-90.	Excluded by abstract
897	Phillips JG. The difficult extraction. 1. The first permanent molar. Dent Update. 1987;14(1):23-7.	Excluded by abstract
898	Pupo Arias D, Batista Zaldívar XB, Nápoles González IdJ, Rivero Pérez O. Pérdida del primer molar permanente en niños de 7 a 13 años. Arch méd Camaguey. 2008;12(5).	Excluded by abstract
899	Rajashekhara BS, Keyur JM, Bhavna D, Poonacha KS. Management of early loss of first permanent molar: a new technique. J Indian Soc Pedod Prev Dent. 2012;30(4):349-51.	Excluded by abstract
900	Reyes Martín B, Cuyac Lantigua M, Alfonso Biart B, Mirabal Peón M, Duque Reyes MV, Sánchez Alvarado Y. Pérdida del primer molar permanente en niños de 6-12 años de edad, Colón, 2013. Rev medica electron. 2015;37(3).	Excluded by abstract
901	Rocha TL, Pinzan-Vercelino CRM, Bronfman CN, Janson G, Henriques JFC, Pinzan A. Verticalização de terceiros molares após extração de primeiros molares permanentes. Ortodontia. 2015;48(6):505-10.	Excluded by abstract
902	Russell DM. Extractions in support of orthodontic treatment. Nda j. 1994;45(2):15-9.	Excluded by abstract
903	Saber AM, Altoukhi DH, Horaib MF, El-Housseiny AA, Alamoudi NM, Sabbagh HJ. Consequences of early extraction of compromised first permanent molar: a systematic review. BMC Oral Health. 2018;18(1):59.	Excluded by abstract
904	Sabri R. Multidisciplinary management of permanent first molar extractions. Am J Orthod Dentofacial Orthop. 2021;159(5):682-92.	Excluded by abstract
905	Sayagh M, Maniere-Ezvan A, Vernet C, Muller-Bolla M. Therapeutic decisions in the presence of decayed permanent first molars in young subjects: a descriptive inquiry. Int Orthod. 2012;10(3):318-36.	Excluded by abstract
906	Scheu J, Cerda C, Rojas V. Timely extraction of the first permanent molars severely affected in mixed dentition. J oral res (Impresa). 2019;8(3):263-8.	Excluded by abstract
907	Shargill I, Hutton A. The management of hypomineralized first permanent molar teeth. Dent Update. 2007;34(10):636-8, 40.	Excluded by abstract
908	Silva ITPd. Má oclusão Classe I de Angle tratada com extrações de primeiros molares permanentes. Dental press j orthod (Impr). 2010;15(4):133-43.	Excluded by abstract
909	Stalpers MJP, Booij JW, Bronkhorst EM, Kuijpers-Jagtman AM, Katsaros C. Extraction of maxillary first permanent molars in patients with Class II Division 1 malocclusion. American Journal of Orthodontics and Dentofacial Orthopedics. 2007;132(3):316-23.	Excluded by abstract
910	Sutcliffe P. A longitudinal study of caries experience and extraction of permanent first molars in English children. Community Dentistry and Oral Epidemiology. 1974;2(4):182-6.	Excluded by abstract
911	Valdés-Martínez Sánchez N, Cid Rodríguez MdC, Quiñones Pérez JA, Soler Cárdenas SF, Hernádez Falcón L. Estado del primer molar permanente en niños de 6 a 11 años de edad. Rev medica electron. 2016;38(3).	Excluded by abstract
912	Vudata UK, Vanga NRV, Chandrabhatla SK. The Prevalence of Malocclusion among Six- and Nine-year-old School-going Children of Visakhapatnam: A Cross-sectional Study. Journal of Clinical and Diagnostic Research. 2023;17(6):ZC60-ZC6.	Excluded by abstract
913	Williams JK, Gowans AJ. Hypomineralised first permanent molars and the orthodontist. European Journal of Paediatric Dentistry. 2003;4(3):129-32.	Excluded by abstract
914	Willmott NS, Bryan RA, Duggal MS. Molar-incisor-hypomineralisation: a literature review. European archives of paediatric dentistry : official journal of the European Academy of Paediatric Dentistry. 2008;9(4):172-9.	Excluded by abstract
915	Wu M, Chen L, Bawole E, Anthonappa RP, King NM. Is there sufficient evidence to support an optimum time for the extraction of first permanent molars? Eur Arch Paediatr Dent. 2017;18(3):155-61.	Excluded by abstract
916	Zhang JG. [Spatial changes after extraction of the first permanent molar]. Zhonghua Kou Qiang Yi Xue Za Zhi. 1987;22(2):65-7, 126.	Excluded by abstract
917	Gorczyński M. Problems concerning extraction of the 1st permanent molars. Czasopismo stomatologiczne. 1968;21(5):533-7.	Excluded; fulltext unavailable
918	McEwen JD, Burke PH. [The first permanent molar. 3. Therapeutic aspects of first molar extraction]. Orthod Fr. 1969;40:29-98.	Excluded; fulltext unavailable
919	Pastore A, Gnudi A. [STATISTICAL INVESTIGATION ON THE PREMATURE LOSS OF THE FIRST PERMANENT MOLAR]. Ann Stomatol (Roma). 1965;14:219-24.	Excluded; fulltext unavailable
920	Pop AS, Campean RS, Ilea A, Nemes O, TĂrmure V. The influence of first permananent molar loss on dental arch morphology - A radiological evaluation. Clujul Medical. 2016;89:S174.	Excluded; fulltext unavailable
921	Richardson A. A cephalometric study of the effect of extraction of lower first permanent molars. Journal of Clinical Pediatric Dentistry. 2000;24(3):195-8.	Excluded; fulltext unavailable
922	Spuropoulos ND, Pierrakou ED, Patsakas AI. [The loss of the 1st permanent molar in a sample of Greek and American populations of 8 to 12 years of age]. Odontostomatol Proodos. 1980;34(3):129-42.	Excluded; fulltext unavailable
923	Toutountzakis N, Lagoudakis M, Xeniotou-Voutsina A. [Consequences of lower first permanent molar loss and its treatment in adults]. Stomatologia (Athenai). 1987;44(5):221-39.	Excluded; fulltext unavailable
924	Humphreys J, Graham A, Rodd HD, Albadri S, Parekh S, Somani C, et al. Molar incisor hypomineralisation: Teaching and assessment across the undergraduate dental curricula in the UK. International Journal of Paediatric Dentistry. 2024.	Excluded; not relevant
925	Ida-Bagus N, Vanda R, Ike-Sesaria P, Wulan P. Treatment of a patient with class I malocclusion with moderate crowding and missing first molar: A case report. Acta Medica Philippina. 2023:74-9.	Excluded; not relevant
926	Lin PY, Hsu LY, Ko HLA, Wang PH, Lin PY, Chi LY. Real-world effectiveness of national pit and fissure sealants program in Taiwan. Journal of Dentistry. 2023;135 C7 - 104587.	Excluded; not relevant
927	Tabatabai T, Kjellberg H. Effect of treatment with dental space maintainers after the early extraction of the second primary molar: a systematic review. Eur J Orthod. 2023;45(4):462-7.	Excluded; not relevant
928	Zietsman ST. [The first permanent molar in orthodontics]. J Dent Assoc S Afr. 1980;35(1):11-8.	Excluded; not relevant
929	Bekes K, Steffen R, Krämer N. Update of the molar incisor hypomineralization: Würzburg concept. European Archives of Paediatric Dentistry. 2023;24(6):807-13.	Excluded; no clinical study
930	Chen J, Xu Y, Guo W. Combined orthopedic-orthodontic treatments of adolescent skeletal open-bite with severe molar-incisor hypomineralization: a case report and literature review. Journal of Clinical Pediatric Dentistry. 2023;47(1):91-9.	Excluded; no clinical study
931	Hajdarevic A, Cirgic E, Robertson A, Sabel N, Jälevik B. Treatment choice for first permanent molars affected with molar-incisor hypomineralization, in patients 7-8 years of age: a questionnaire study among Swedish general dentists, orthodontists, and pediatric dentists. European Archives of Paediatric Dentistry. 2024.	Excluded; no clinical study

932	Lakhani S, Noble F, Rodd H, Cobourne MT. Management of children with poor prognosis first permanent molars: an interdisciplinary approach is the key. British Dental Journal. 2023;234(10):731-6.	Excluded; no clinical study
933	Legris S. [MIH and compromised permanent first molars. When and which teeth to extract: guidelines]. Orthod Fr. 2023;94(3-4):485-511.	Excluded; no clinical study
934	Hom BM, Turley PK. The effects of space closure of the mandibular first molar area in adults. American Journal of Orthodontics. 1984;85(6):457-69.	Excluded; study on adults
935	Freitas MTDd, Tiveron ARF, Pastori CM, Ferreira JPR. Exodontia de primeiro molar superior permanente em criança: relato de caso. Rev Odontol Araçatuba (Impr). 2023;44(2):13-7.	Excluded; case report
936	Murphy I, Noar JH. Early Extraction of First Permanent Molars: Applying the Evidence. Dental Update. 2022;49(7):562-6.	Excluded; case report
937	Zhang D, Shi Q, Fang L, Jiang W, Han J, Wu Z. Management Of Ectopic Eruption Of Bilateral Mandibular First Permanent Molars: A Case Report And Literature Review. J Pak Med Assoc. 2023;73(9):1897-9.	Excluded; case report
938	Cardoso PC, Mecenas P, Normando D. The impact of the loss of first permanent molars on the duration of treatment in patients treated with orthodontic space closure and without skeletal anchorage. Prog Orthod. 2022;23(1):32.	Excluded; orthodontic treatment
939	Jacobs C, Jacobs-Müller C, Luley C, Erbe C, Wehrbein H. Orthodontic space closure after first molar extraction without skeletal anchorage. J Orofac Orthop. 2011;72(1):51-60.	Excluded; orthodontic treatment
940	Bakkal M, Yilmaz B, Kaya MS, Unver T, Taran PK, Ozdemir S. Timing for extraction of permanent first molars in school aged children: a pilot study. Journal of Clinical Pediatric Dentistry. 2024;48(1):78-84.	Excluded; other outcome
941	Cağlaroğlu M, Kilic N, Erdem A. Effects of early unilateral first molar extraction on skeletal asymmetry. Am J Orthod Dentofacial Orthop. 2008;134(2):270-5.	Excluded; other outcome
942	Langer LJ, Pandis N, Mang de la Rosa MR, Jost-Brinkmann PG, Bartzela TN. Eruption Pattern of Third Molars in Orthodontic Patients Treated with First Permanent Molar Extraction: A Longitudinal Retrospective Evaluation. Journal of Clinical Medicine. 2023;12(3).	Excluded; other outcome
943	Murphy I, Noar J, Parekh S, Ashley P. The effect of extraction of the lower first permanent molar on the developing third molar in children. J Orthod. 2022;49(4):480-7.	Excluded; other outcome
944	Sanghvi R, Cant A, de Almeida Neves A, Hosey MT, Banerjee A, Pennington M. Should compromised first permanent molar teeth in children be routinely removed? A health economics analysis. Community Dent Oral Epidemiol. 2023;51(5):755-66.	Excluded; other outcome
945	Surme K, Akman H, Cimen T, Akin M. Effects of Early Unilateral Mandibular First Molar Extraction on Condylar and Ramal Asymmetry. International Journal of Morphology. 2023;41(2):395-400.	Excluded; other outcome
946	Teo TK, Ashley PF, Derrick D. Lower first permanent molars: developing better predictors of spontaneous space closure. Eur J Orthod. 2016;38(1):90-5.	Excluded; other outcome
947	Yavuz I, Baydaş B, Ikbal A, Dağsuyu IM, Ceylan I. Effects of early loss of permanent first molars on the development of third molars. Am J Orthod Dentofacial Orthop. 2006;130(5):634-8.	Excluded; other outcome
948	Aldahool Y, Sonesson M, Dimberg L. Spontaneous space closure in patients treated with early extraction of the first permanent molar: a retrospective cohort study using radiographs. Angle Orthod 2024 Mar 1;94(2):180-186.	Included
949	Brusevold IJ, Kleivene K, Grimsøen B, Skaare AB. Extraction of first permanent molars severely affected by molar incisor hypomineralisation: a retrospective audit. Eur Arch Paediatr Dent. 2022;23(1):89-95.	Included
950	Canpolat MK, Demirel A, Aydınbelge M, Sarı Ş. The Effects of Uncontrolled Extractions of the Permanent Lower First Molars on the Prognosis of Spontaneous Space Closure and Occlusion. Cumhuriyet Dent J 2020;23:3:181-190.	Included
951	Ciftci V, Guney AU, Deveci C, Sanri IY, Salimow F, Tuncer AH. Spontaneous space closure following the extraction of the first permanent mandibular molar. Niger J Clin Pract. 2021;24(10):1450-6.	Included
952	Ertuğrul C, Ozbey H, Gün AI. Early extraction of the first permanent molars: a five-year follow-up study. Eur J Paediatr Dent. 2022;23(2):111-5.	Included
953	Gaudreau G, Rofaeel M, Dabbagh B, dos Santos BF. Clinical and radiographic factors influencing space closure following the extraction of permanent first molar. Journées dentaires internationales du Québec 2022.	Included
954	Jälevik B, Möller M. Evaluation of spontaneous space closure and development of permanent dentition after extraction of hypomineralized permanent first molars. International Journal of Paediatric Dentistry. 2007;17(5):328-35.	Included
955	Lenaker D, Grünheid T. Radiographic Evaluation of Predictors for Spontaneous Space Closure and Supereruption After the Loss of Permanent First Molars in Yup'ik Children. Pediatr Dent. 2023 May 15;45(3):252-258.	Included
956	Lenaker DC. Evaluation of Predictors of Spontaneous Space Closure and Supereruption after the Loss of First Permanent Molars. MSc Thesis, University of Minesota, 2022.	Included
957	Mouroutsou EK. Clinical and radiographic evaluation of spontaneous space closure after extraction of first permanent molars in young patients. MSc Thesis, Ghent University, 2018.	Included
958	Nordeen KA, Kharouf JG, Mabry TR, Dahlke WO, Beiraghi S, Tasca AW. Radiographic Evaluation of Permanent Second Molar Substitution After Extraction of Permanent First Molar: Identifying Predictors for Spontaneous Space Closure. Pediatr Dent. 2022;44(2):123-30.	Included
959	Patel S, Ashley P, Noar J. Radiographic prognostic factors determining spontaneous space closure after loss of the permanent first molar. Am J Orthod Dentofacial Orthop. 2017;151(4):718-26.	Included
960	Rahhal AA. Extraction Timing of Heavily Destructed Upper First Permanent Molars. Open J Stomatol 2014;4:161-168.	Included
961	Serindere G, Bolgul B, Parlar T, Cosgun A. Effects of first permanent molar extractıon on space changes observed in the dental arch using data mining method. Niger J Clin Pract. 2019;22(7):936-42.	Included
962	Teo TK, Ashley PF, Parekh S, Noar J. The evaluation of spontaneous space closure after the extraction of first permanent molars. Eur Arch Paediatr Dent. 2013;14(4):207-12.	Included
963	Thilander B, Skagius S. Orthodontic sequelae of extraction of permanent first molars. A longitudinal study. Rep Congr Eur Orthod Soc. 1970:429-42.	Included

Appendix 5. Re-analysis of Aldahool 2024 data: Identification of confounders for the effect of jaw on the eruption of the pM2 with the CIE method.

**Adjusted for**	**Coefficient**	**% CIE**	**Selected**
Crude (none)	-1.7228	-	-
Side	-1.7343	0.67%	-
Sex	-1.7146	-0.48%	-
age	-2.8856	67.50%	Selected
pM2 inclination	-1.5107	-12.31%	-
pM2 vertical position	-1.9223	11.58%	-
pM3 presence	-1.7639	2.39%	-
pM2 developmental stage	-3.1836	84.79%	Selected
pM2 developmental stage (categorized)	-2.729659	58.44%	Selected

CIE, change in estimate; pM2/3, permanent 2nd/3rd molar.

Appendix 6. Re-analysis of Aldahool 2024 data: Effect of various factors on the on the eruption of the pM2, stratified by jaw.

		**Maxilla**			**Mandible**	
	**Category**	**OR (95% CI)**	**P**		**OR (95% CI)**	**P**
Sex	Female	Reference			Reference	
	Male	0.24 (0.02, 2.28)	0.21		1.75 (0.59, 5.18)	0.31
Age	Per year	**0.18 (0.07, 0.44)**	**<0.001**		**0.51 (0.34, 0.76)**	**0.001**
Age category #1	< 12 years	Reference			Reference	
	≥ 12 years	**<0.01 (<0.01, <0.01)**	**<0.001**		**0.09 (0.03, 0.33)**	**<0.001**
Age category #2	8.00 - 8.99	Reference			Reference	
	9.00 - 9.99	1.00 (0.57, 1.76)	1.00		**<0.01 (<0.01, <0.01)**	**<0.001**
	10.00 - 10.99	1.00 (0.44, 2.26)	1.00		**<0.01 (<0.01, <0.01)**	**<0.001**
	11.00 - 11.99	1.00 (0.53, 1.89)	1.00		**<0.01 (<0.01, <0.01)**	**<0.001**
	12.00 - 12.99	1.00 (0.51, 1.97)	1.00		**<0.01 (<0.01, <0.01)**	**<0.001**
	13.00 - 13.99	1.00 (0.45, 2.20)	1.00		**<0.01 (<0.01, <0.01)**	**<0.001**
	≥14.00	**<0.01 (<0.01, <0.01)**	**<0.001**		**<0.01 (<0.01, <0.01)**	**<0.001**
Side	Left	Reference			Reference	
	Right	1.23 (0.19, 7.75)	0.83		1.18 (0.45, 3.05)	0.74
pM2 inclination	Per degree	1.05 (0.96, 1.16)	0.30		1.02 (0.94, 1.10)	0.71
pM2 vertical position	Per stage	3.71 (0.92, 15.32)	0.06		**2.92 (1.07, 7.98)**	**0.03**
pM2 development #1	A-D	**>100.00 (>100.00, >100.00)**	**<0.001**		**9.33 (1.25, 69.44)**	**0.02**
	E-F	8.00 (0.83, 77.12)	0.07		**42.00 (9.36, >100.00)**	**<0.001**
	G-H	Reference			Reference	
pM2 development #2	D	**>100.00 (>100.00, >100.00)**	**<0.001**		0.21 (0.02, 1.82)	0.16
	E	Reference			Reference	
	F	**>100.00 (>100.00, >100.00)**	**<0.001**		0.85 (0.11, 6.27)	0.87
	G	**>100.00 (>100.00, >100.00)**	**<0.001**		**0.05 (0.01, 0.35)**	**0.003**
	H	0.13 (0.01, 1.32)	0.08		**0.01 (<0.01, 0.11)**	**<0.001**
pM3 presence	No	Reference			Reference	
	Yes	3.50 (0.54, 22.77)	0.19		1.69 (0.55, 5.20)	0.36

CI, confidence interval; OR, odds ratio; pM2/3, permanent 2nd/3rd molar.

Appendix 7. Re-analysis of Ertugrul 2022 data: Identification of confounders for the effect of jaw on the eruption of the pM2 with the CIE method.

**Adjusted for**	**Coefficient**	**% CIE**	**Selected**
Crude (none)	0.3101549	-	-
Side	0.3364111	8.47%	-
age	0.5101195	64.47%	**Yes**
pM3 presence	0.6908852	122.75%	**Yes**
pM2 developmental stage	0.2226719	-28.21%	**No**
pM2 developmental stage (categorized)	0.8813619	184.17%	**Yes**
pM2 developmental stage (E vs others)	0.1140953	-63.21%	**No**

CIE, change in estimate; pM2/3, permanent 2nd/3rd molar.

Appendix 8. Outcomes analyzed by single studies that could, therefore, not be meta-analyzed.

Nr	Outcome	Experimental group	Control group	OR (95% CI)	P	SS	Clinically relevant*
1	SSC (overall)	Class II malocclusion	Class I malocclusion	2.34 (0.98, 5.59)	0.057	-	-
2	SSC (overall)	Class III malocclusion	Class I malocclusion	0.52 (0.20, 1.37)	0.186	-	-
3	SSC (overall)	Crowding	Spacing	5.88 (1.25, 27.67)	0.025	Yes	Yes
4	SSC (mandible)	M2 NS 6-7	M2 NS 8-9	55.09 (3.02, >100.0)	0.007	Yes	Yes
5	M2 rotation (mandible)	M2 NS 6-7	M2 NS 8-9	0.22 (0.04, 1.21)	0.082	-	-
6	PM1 rotation (mandible)	M2 NS 6-7	M2 NS 8-9	0.32 (0.10, 1.00)	0.051	-	-
7	PM2 rotation (mandible)	M2 NS 6-7	M2 NS 8-9	0.42 (0.13, 1.36)	0.149	-	-
8	ML deviation (mandible)	Unilateral	Bilateral	11.58 (1.91, 70.26)	0.008	Yes	Yes
9	SSC (mandible)	Follow-up (per year)		1.38 (1.13, 1.69)	0.002	Yes	No

CI, confidence interval; DS, Demirjian tooth development stage; ML, midline; NS, Nolla tooth development stage; OR, odds ratio; PM, premolar; SS, statistically significant; SSC, spontaneous space closure; M2, second permanent molar.
* arbitrarily judged as OR > 1.50 or OR < 0.67.

Appendix 9. Results of meta-regressions for indirect meta-analyses of spontaneous space closure.

**Outcome**	**Factor**	**Level**	**Studies**	**Coefficient**	**95% CI**	**P**
Spontaneous space closure (maxilla)	Age	Per year	9	-0.50	-1.14, 0.15	0.13
	% male	Per 10%	6	-0.53	-1.63, 0.56	0.34
Spontaneous space closure (mandible)	Age	Per year	11	-0.08	-0.65, 0.49	0.78
	% male	Per 10%	9	-0.25	-1.02, 0.52	0.53

CI, confidence interval.

Appendix 10. Contour-enhanced funnel plot for the overall prevalence of spontaneous space closure in the mandible (n=11).

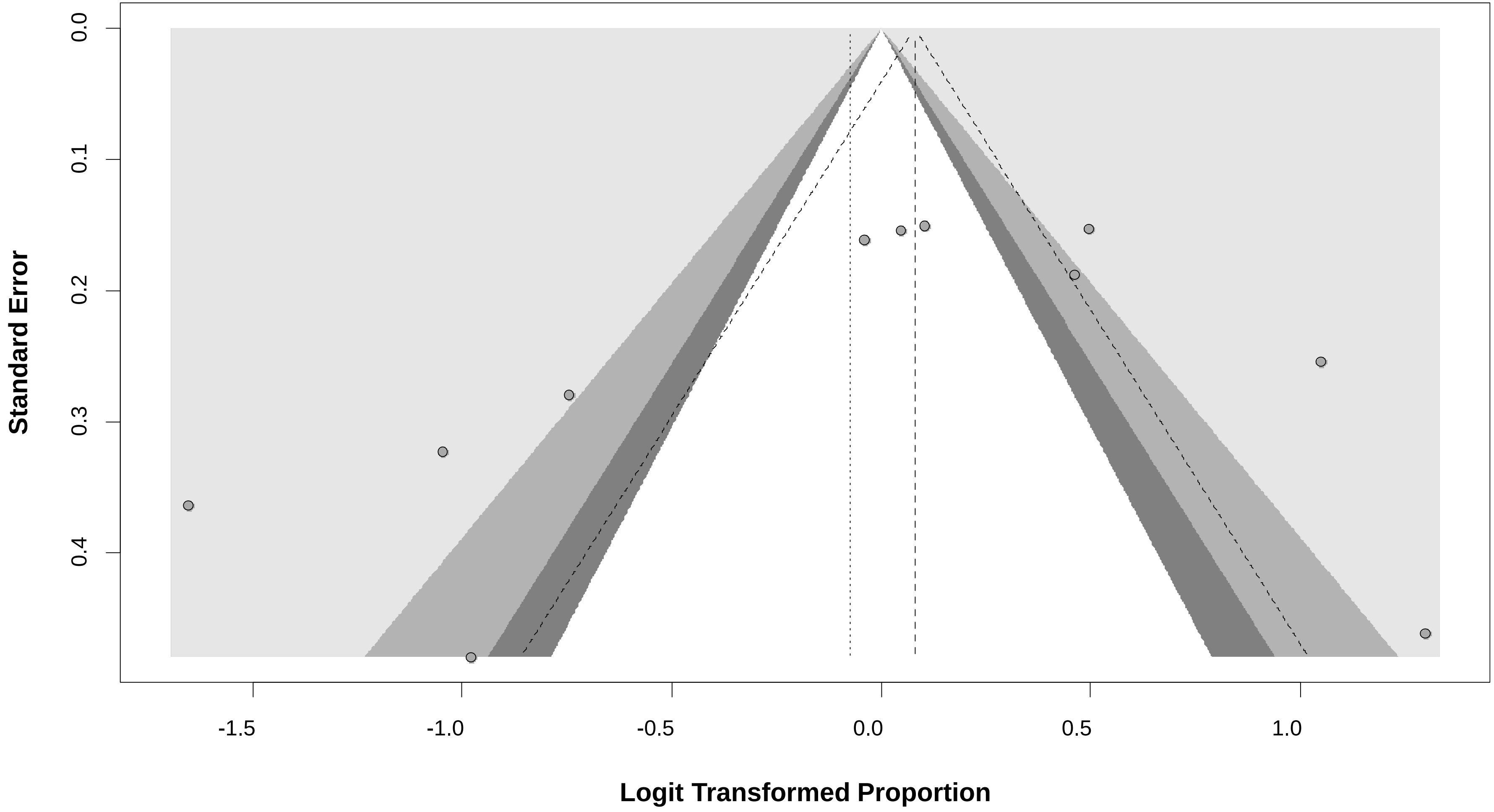

Appendix 11. Sensitivity analysis according to publication type of included studies.

	**Gray literature**		**Journal papers**		
**Outcome**	**Studies**	**Effect (95% CI)**	**Studies**	**Effect (95% CI)**	P_{SG}
Spontaneous space closure (overall)	1	PrV 60.0% (46.5%, 65.0%)	12	PrV 62.1% (49.6%, 73.1%)	0.40
Spontaneous space closure (maxilla)	1	PrV 84.0% (71.1%, 91.8%)	8	PrV 85.5% (71.7%, 93.2%)	0.83
Spontaneous space closure (mandible)	1	PrV 32.2% (21.6%, 45.1%)	10	PrV 49.8% (35.0%, 64.6%)	0.06
Spontaneous space closure in the maxilla versus the mandible	2	OR 9.06 (0.41, >100.00)	7	OR 7.68 (4.18, 14.09)	0.63

CI, confidence interval; OR, odds ratio; PrV, prevalence; PSG, p value for differences between subgroups.
